# Quantitative biokinetics over a 28 day period of freshly generated, pristine, 20 nm titanium dioxide nanoparticle aerosols in healthy adult rats after a single two-hour inhalation exposure

**DOI:** 10.1186/s12989-019-0303-7

**Published:** 2019-07-09

**Authors:** Wolfgang G. Kreyling, Uwe Holzwarth, Carsten Schleh, Stephanie Hirn, Alexander Wenk, Martin Schäffler, Nadine Haberl, Manuela Semmler-Behnke, Neil Gibson

**Affiliations:** 1 Comprehensive Pneumology Center, Institute of Lung Biology and Disease, Helmholtz Zentrum München – German Research Center for Environmental Health, Ingolstaedter Landstrasse 1, D-85764 Munich, Neuherberg Germany; 2 Institute of Epidemiology, Helmholtz Center Munich – German Research Center for Environmental Health, Ingolstaedter Landstrasse 1, D-85764 Munich, Neuherberg Germany; 3 European Commission, Joint Research Centre (JRC), Ispra, Italy; 4 Current address: Abteilung Gesundheit, Berufsgenossenschaft Holz und Metall, Am Knie 8, D-81241 München, Germany; 5 Current address: Dept. Infrastructure, Safety, Occupational Protection, Helmholtz Center München – German Research Center for Environmental Health, Ingolstaedter Landstrasse 1, D-85764 Munich, Neuherberg Germany

**Keywords:** Spark ignition generated titanium dioxide nanoparticle aerosols, Characterization of physicochemical particle properties, Intratracheal inhalation of freshly generated aerosols, Translocation across air-blood-barrier, Accumulation in secondary organs and tissues, Long-term alveolar macrophage-mediated nanoparticle clearance, Nanoparticle relocation into the interstitium, Re-entrainment from interstitium back to lung epithelium for clearance towards the larynx

## Abstract

**Background:**

Industrially produced quantities of TiO_2_ nanoparticles are steadily rising, leading to an increasing risk of inhalation exposure for both professionals and consumers. Particle inhalation can result in inflammatory and allergic responses, and there are concerns about other negative health effects from either acute or chronic low-dose exposure.

**Results:**

To study the fate of inhaled TiO_2_-NP, adult rats were exposed to 2-h intra-tracheal inhalations of ^48^V-radiolabeled, 20 nm TiO_2_-NP aerosols (deposited NP-mass 1.4 ± 0.5 μg). At five time points (1 h, 4 h, 24 h, 7d, 28d) post-exposure, a complete balance of the [^48^V]TiO_2_-NP fate was quantified in organs, tissues, carcass, lavage and body fluids, including excretions.

After fast mucociliary airway clearance (fractional range 0.16–0.31), long-term macrophage-mediated clearance (LT-MC) from the alveolar region is 2.6-fold higher after 28d (integral fraction 0.40 ± 0.04) than translocation across the air-blood-barrier (integral fraction 0.15 ± 0.01). A high NP fraction remains in the alveoli (0.44 ± 0.05 after 28d), half of these on the alveolar epithelium and half in interstitial spaces. There is clearance from both retention sites at fractional rates (0.02–0.03 d^− 1^) by LT-MC. Prior to LT-MC, [^48^V]TiO_2_-NP are re-entrained to the epithelium as reported earlier for 20 nm inhaled gold-NP (AuNP) and iridium-NP (IrNP).

**Conclusion:**

Comparing the 28-day biokinetics patterns of three different inhaled NP materials TiO_2_-NP, AuNP and IrNP, the long-term kinetics of interstitial relocation and subsequent re-entrainment onto the lung-epithelium is similar for AuNP and Ir-NP but slower than for TiO_2_-NP. We discuss mechanisms and pathways of NP relocation and re-entrainment versus translocation. Additionally, after 28 days the integral translocated fractions of TiO_2_-NP and IrNP across the air-blood-barrier (ABB) are similar and become 0.15 while the translocated AuNP fraction is only 0.04. While NP dissolution proved negligible, translocated TiO_2_-NP and IrNP are predominantly excreted in urine (~ 0.1) while the urinary AuNP excretion amounts to a fraction of only 0.01. Urinary AuNP excretion is below 0.0001 during the first week but rises tenfold thereafter suggesting delayed disagglomeration. Of note, all three NP dissolve minimally, since no ionic radio-label release was detectable. These biokinetics data of inhaled, same-sized NP suggest significant time-dependent differences of the ABB translocation and subsequent fate in the organism.

**Electronic supplementary material:**

The online version of this article (10.1186/s12989-019-0303-7) contains supplementary material, which is available to authorized users.

## Background

The quantities of TiO_2_ nanoparticles with different characteristics produced by industry are steadily increasing due to their versatile and broadening, application spectrum. Over 50.000 tons per year was the estimate in 2010 [1]. It has been suggested that by 2026 the majority of bulk TiO_2_ production may be in the form of nanoparticles following an exponential growth in TiO_2_ nanoparticle use, reaching up to 2.5 million tons per year [1]. Even without such exponential growth, several market analyses (published in 2013) indicate an increase in the annual production of TiO_2_ nanoparticles by an order of magnitude up to 2020 (e.g. [2]). This clearly entails an increasing risk of inhalation exposure particularly from products such as pigments, as well as an increased risk of professional exposure during manufacturing and packaging [3]. Following exposure, particle translocation from lungs to systemic vasculature [4, 5], where previously inert particles may adopt a new biological identity after formation of a protein corona [4, 6–8], could lead to negative health outcomes. In this context, the allergic potential of nanoparticles and their potential to trigger asthma effects are under scrutiny [9, 10], and TiO_2_ nanoparticles have even been classified as “possibly carcinogenic to humans” [11, 12] supported by a recommendation of ECHA for classification of TiO_2_ [13]. On the other hand, the application of TiO_2_ nanoparticles as pulmonary drug delivery systems via inhalation is being considered for the treatment of respiratory diseases, as these may allow more direct access to diseased tissue thereby avoiding risks related with systemic drug administration [14–18].

In a pioneering inhalation study, Ferin, and co-workers [19] demonstrated that nano-sized TiO_2_-particles are able to cross the air-blood-barrier (ABB) of rats to a greater extent than submicron TiO_2_-particles, thereby causing more inflammation than the same airborne mass concentration of larger particles. This report provided the first evidence that inhaled TiO_2_-material, with low toxicity in the form of submicron particles, may exhibit increased toxicity when the particle size is reduced to the nanometer range [19]. It has also been demonstrated that an equivalent dose of TiO_2_-NP applied by IT-instillation and whole body inhalation causes a significantly greater acute respiratory tract inflammation when the dose-rate of TiO_2_-NP delivery is increased [20].

In a review of inhalation studies, Shi et al. [21] showed that occupational TiO_2_-particle concentrations may reach currently established limits of 5 mg•m^− 3^ while non-professional environmental exposure levels may reach up to 50 μg•m^− 3^ [21]. Assuming a typical daily inhalation volume of 20 m^3^ air and a deposited fraction of 30% in the lungs [22] the daily deposited lung dose can, therefore, vary between 0.3 mg•d^− 1^ for a member of the public and up to 10 mg•d^− 1^ for exposed workers (8 h of exposure). When normalized to body weight (BW, assuming 70 kg for an adult person) this translates into exposure doses in the range between 4.3 μg•kg^− 1^ to 143 μg•kg^− 1^, levels that should be used as an indication of approximate upper limits in meaningful in vivo inhalation studies (at least at current exposure levels). Studies should also be designed to take into account the accumulation dynamics of nanoparticles: from previous biokinetics studies of inhaled, 20 nm gold nanoparticles [23], or of inhaled, 20 nm iridium nanoparticles [24–26], or of 20 nm elemental carbon nanoparticles [27], it is known that nanoparticle accumulation occurs rather rapidly during the first 24-h.

Therefore, after a two-hour inhalation of 20 nm TiO_2_-NP radio-labeled with ^48^V, the present investigation covers early accumulation with three time-points of 1 h, 4 h, and 24 h, followed by two time-points after 7 days and 28 days to investigate possibly slower processes of accumulation, redistribution, and clearance of nanoparticles. For this purpose, retained [^48^V]TiO_2_-NP doses in different organs of interest, selected tissues and body fluids were determined, including the carcass and the entire fecal and urinary excretions of each animal to provide a complete balance of the fate of the applied [^48^V]TiO_2_-NP in the entire organism.

This fourth biokinetics study on inhaled 20 nm [^48^V]TiO_2_-NP applying the same inhalation methodology to the same strain of rats and the same biokinetics analysis of radio-labeled NP like the previous studies on gold-NP [23], iridium-NP [24–26] and elemental carbon NP [27] allows the unique option of comparing the in vivo fate of different inhaled NP materials and their according physicochemical characteristics. Furthermore, the present inhalation/biokinetics study will be compared with our previous series of 28-day biokinetics studies obtained after intratracheal instillation or intravenous injection or oral gavage of 70 nm [^48^V]TiO_2_-NP in the same strain of rats [28–30].

## Results

### Aims and rationale

The present study was designed to investigate the quantitative biokinetics of TiO_2_-NP after a single two-hours intratracheal inhalation exposure of ^48^V radio-labeled, 20 nm TiO_2_-NP ([^48^V]TiO_2_-NP) over a period of up to 28 days. Female, adult Wistar-Kyoto rats inhaled a [^48^V]TiO_2_-NP aerosol freshly generated by spark ignition between two proton-irradiated pure Ti electrodes. The study was designed as part of a series of studies to compare the biokinetics of three different inhaled 20-nm NP-materials – TiO_2_-NP, AuNP and IrNP [23–26] including their translocation kinetics across the air-blood-barrier (ABB) and their subsequent accumulation (up to 28 days; due to gradual NP uptake from the blood) in secondary organs and tissues in the same strain of rats and using the same inhalation technology. The added value of these studies is derived from the adjusted and agglomeration-controlled small size of the inhaled nanoparticles since other inhalation studies applied NP agglomerates which were much larger than 20 nm. Moreover, the use of radiolabeled NP provides the required precision to study translocation kinetics across the ABB and also to quantify minor NP accumulations in secondary organs and tissues.

Since we have previously observed relocation of NP after intratracheal inhalation of 20 nm [^195^Au] AuNP [23] and [^192^Ir] IrNP [24–26] into the alveolar interstitium and subsequent re-entrainment back onto the lung epithelial surface we hypothesized that the same kinetics may be detectable for inhaled [^48^V]TiO_2_-NP of the same size.

Furthermore, the present inhalation study will be compared with our previous biokinetics data obtained after intratracheal instillation or intravenous injection or oral gavage of 70 nm [^48^V]TiO_2_-NP in the same strain of rats [28–30].

### [^48^V]TiO_2_-NP aerosol exposure and deposition

Table 1 compiles the key characterization parameters of the aerosols used for each group of rats in the biokinetics study. These were derived from in situ measurements during inhalation exposure using a Scanning Mobility Particle Sizer (SMPS) Spectrometer and a Condensation Particle Counter (CPC) synchronized with the flight time required until the inhalation by the rats (assuring the correct size distribution at inhalation), as well as γ-spectrometry results on a filter collecting a precise fraction of the aerosol in a bypass line. As described in detail in the Additional file 1 the count medium diameter (CMD) and its geometric standard deviation (GSD) are obtained from the as-measured particle size spectra. Additionally, the experimentally determined particle size spectra were averaged and then fitted to a log-normal distribution, applying a least squares method. The fitted log-normal distribution was extrapolated down to a particle size of 1 nm in order to overcome the operative threshold of the SMPS of 10 nm and to estimate the contributions of smaller particles.

**Table 1:** Aerosol parameters determined over the entire intratracheal inhalation period of 2 h for each exposure

Group of rats	Instrument	1 h	4 h	24 h	7d	28d
CMD (nm), mean ± SD of 40 spectra*	SMPS	23.56 ± 0.68	23.66 ± 1.01	21.61 ± 0.77	20.18 ± 1.25	23.57 ± 0.94
CMD (nm) of averaged + extrapolated spectrum^&^	SMPS	23.00	23.00	20.00	20.00	20.00
Geom. Std. Dev. (GSD) mean ± SD of 40 spectra*	SMPS	1.48 ± 0.03	1.46 ± 0.02	1.44 ± 0.03	1.45 ± 0.09	1.48 ± 0.03
GSD of averaged + extrapolated spectrum^&^	SMPS	1.45	1.45	1.45	1.45	1.45
TiO_2_-NP number concen-tration (10^6^ × #/cm^3^) ^&^	SMPS	2.23 ± 0.12	2.76 ± 0.18	2.23 ± 0.15	2.6 ± 2.08	2.65 ± 0.21
TiO_2_-NP number concen-tration (10^6^ × #/cm^3^)	CPC	2.47 ± 0.86	3.37 ± 0.415	2.51 ± 0.099	2.41 ± 0.168	2.89 ± 0.091
Median diameter (nm) of volume concentration	SMPS	36.03 ± 2.51	34.56 ± 1.42	31.89 ± 1.33	29.73 ± 0.82	35.45 ± 0.79
TiO_2_-NP volume concen-tration ^+^ (10^−5^ cm^3^/m^3^)	SMPS	2.76 ± 0.42	3.29 ± 0.34	2.1 ± 0.29	1.53 ± 0.08	3.32 ± 0.28
Specific ^48^V aerosol activity on filter (kBq/L)^§^	filter γ-spec-trometer	1.86	2.37	1.31	1.08	2.03
Mean spec. ^48^V act. of TiO_2_-NP (kBq/μg; filter Act / (Vol * dens)	filter, SMPS, γ.spectrom.	12.41	18.49	14.51	15.34	15.72
Mean TiO_2_-NP aerosol mass conc (filter) (μg/L) ^#^	filter, SMPS, γ-spectrom.	0.15	0.13	0.09	0.07	0.13
derived porous TiO_2_-NP density (g/cm^3^)	filter, SMPS, γ-spectrom.	1.83	2.73	2.14	2.27	2.32

Aerosol parameters (mean ± SD; n = 4 of each group)

^1^ SMPS data were analyzed by AIM - Instrument Manager Software of TSI Inc. version 7.2.5.0

* Mean of count median diameters (CMD) and geometric standard deviations (GSD) of all 40 SMPS spectra measured in the size range 10 nm - 420 nm

^&^ CMD and GSD of the number size spectrum averaged over all 40 SMPS spectra and mean-square fitted and extrapolated to 1-nm size; see Additional file 1

^+^ Integral particle volume concentration (PVC) of the aerosol distribution (cm^3^/m^3^) SMPS derived

^§^ Specific [^48^V]TiO_2_-NP aerosol activity determined from an aerosol filter sample continuously collected during each 2-h exposure at 0.3 L/min according to eq. 1 of Additional file 1

^#^ Derived [^48^V]TiO_2_-NP aerosol mass concentration (μg/L) dividing the specific ^48^V aerosol activity (filter, kBq/L) by the specific [^48^V]TiO_2_-NP activity concentration 12.4 kBq/μg

Intratracheal inhalation exposure allowed deep breath ventilation and avoided head airway deposition, thus leading to enhanced intrathoracic conducting airway deposition as well as alveolar deposition, with long-term alveolar retention being the dominating outcome. Parameters of aerosol inhalation and deposition are compiled in Table 2 for each group of rats. In addition, the activity fractions cleared from the lungs that can be attributed to fast mucociliary clearance from the conducting airways (MCC) and long-term macrophage-mediated [^48^V]TiO_2_-NP clearance (LT-MC) are indicated for each group of rats. With a mean rat body weight of 277 ± 17 g, the mean deposited TiO_2_-NP mass of 4.7 μg•kg^− 1^ matches well with the approximate maximum daily exposure dose of 4.3 μg•kg^− 1^ for members of the public mentioned in the introduction.

**Table 2:** Summary of the inhalation parameters and basic results of the biokinetics study

Time point after inhalation	1 h	4 h	24 h	7d	28d
Inhaled aerosol volume (L) ^1^	39.37 ± 1.12	42.94 ± 2.04	38.13 ± 0.96	38.97 ± 1.01	42.77 ± 1.66
Inhaled [^48^V]TiO_2_-NP activity (kBq) ^1^	34.54 ± 0.98	44.76 ± 2.12	27.57 ± 0.7	24.56 ± 0.63	51.84 ± 2.01
Deposited ^[48^V]TiO_2_-NP activity (kBq) ^1^	20.37 ± 2.21	27.94 ± 9.79	15.64 ± 5.69	12.26 ± 2.85	21.55 ± 6.19
Deposited TiO_2_-NP mass (μg) ^1^	1.64 ± 0.18	1.51 ± 0.53	1.08 ± 0.39	0.8 ± 0.19	1.37 ± 0.39
Deposited fraction per inhaled aerosol ^1^	0.59 ± 0.08	0.39 ± 0.12	0.57 ± 0.21	0.50 ± 0.13	0.42 ± 0.13
Deposited alveolar [^48^V]TiO_2_-NP fraction per IPLD ^1^	0.95 ± 0.03	0.75 ± 0.12	0.72 ± 0.04	0.69 ± 0.02	0.74 ± 0.02
Mucociliary cleared fraction per ILD ^1^	0.04 ± 0.03	0.20 ± 0.12	0.21 ± 0.03	0.26 ± 0.04	0.26 ± 0.04
Long-term macrophage- mediated cleared fraction per ILPD				0.15 ± 0.03	0.31 ± 0.04

^1^according to Eq. (4 through 14) of the Additional file 1

Parameters of the intratracheal [^48^V]TiO_2_-NP aerosol inhalation and deposition are: inhaled aerosol volume and ^48^V activity, deposited ^48^V activity and corresponding [^48^V]TiO_2_-NP mass, deposited ^48^V activity as a fraction of the inhaled ^48^V activity and the alveolar deposited fraction relative to the total lung deposit. Additionally, the fast mucociliary [^48^V]TiO_2_-NP clearance (MCC) during the first two days and the long-term macrophage-mediated clearance (LT-MC) of [^48^V]TiO_2_-NP from day 3–28 after inhalation are presented, calculated based on fecal excretion and retention in the gastro-intestinal-tract (GIT). MCC fractions are normalized to the initial lung dose (ILD) i.e. the sum of all organ and tissue ^48^V radioactivities

The estimated deposited [^48^V]TiO_2_-NP fraction relative to the inhaled aerosol shows considerable inter-subject variability indicating that the tidal volume calculated from Eq. (8) of the Additional file 1 is only a rough estimate. In addition, these data are lower than the deposited fraction of 0.75 as calculated by the MPPD software 3.04 (see Additional file 1: Figure S3 of the Supplementary Information). On the other hand, our experimental MCC data estimated from tracheobronchial deposition (TB) determined 24 h, 7d, and 28d p.e. are about twice as high as the value of 0.12 predicted by the MPPD model (see Additional file 1: Figure S3, of the Supplementary Information). Both differences might be attributed to the exposure conditions during intratracheal low-pressure ventilation in the plethysmograph chamber [31] (for details see the Additional file 1).

The increasing MCC data from 1 h to 4 h p.e. clearly show that MCC is very fast and only a small further increase occurs up to 24 h. Up to this point, the MCC is calculated from the fractions found in the gastro-intestinal tract (GIT) and in feces. The MCC data for the groups of 7 days and 28 days p.e. are derived from fecal excretion measurements during the first 3 days p.e. After the third day, fecal excretion is attributed to LT-MC (see footnote^1^).

### [^48^V]TiO_2_-NP retention in the lungs and BAL

**Fig. 1:**
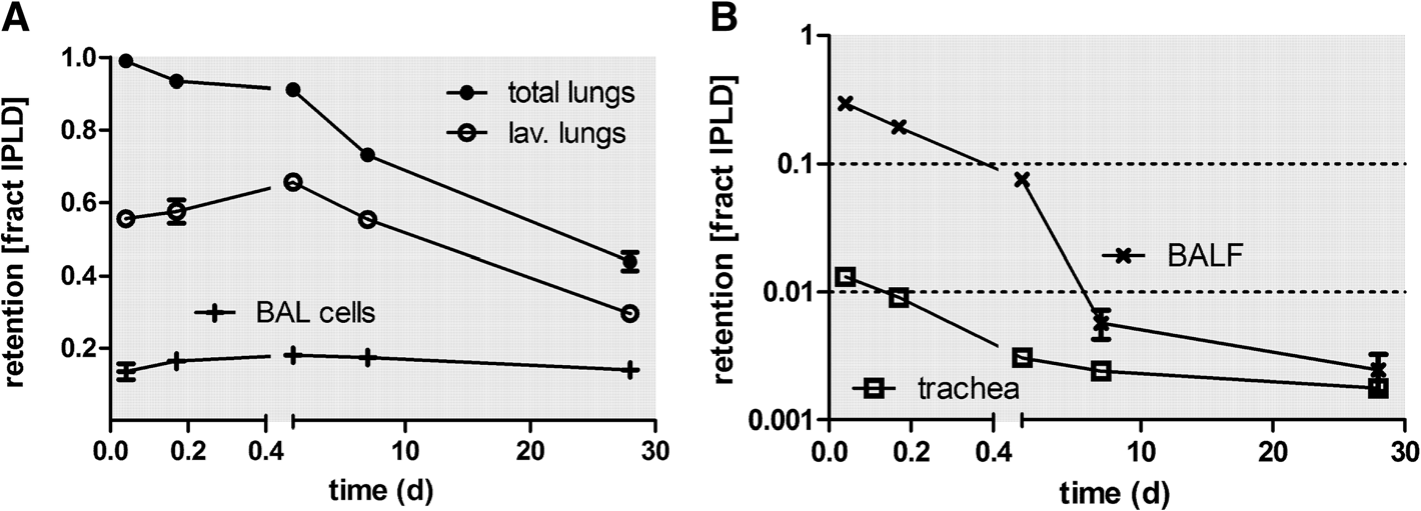
Fractional retention of intratracheally inhaled [^48^V]TiO_2_-NP in the total lungs and lavaged lungs between 1 h and 28 days p.e. Besides lung retention data, recoveries in BALC, BALF and in the trachea sample (trachea with main bronchi) are also presented. Panel **a**: linear y-axis for lung retention and BALC; panel **b**: logarithmic y-axis for BALF and in the trachea sample. The data are given as fractions of IPLD; i.e. corrected for fast [^48^V]TiO_2_-NP clearance from airways (MCC); the mean IPLD in mass (number) of [^48^V]TiO_2_-NP of all five retention time points is 1.01 ± 0.55 μg (2.67 ± 1.16•10^10^ #). Fractional data are given as mean ± SEM, n = 4 rats/time point. Data in both panels are corrected for [^48^V]TiO_2_-NP retained in the residual blood volume of the lungs. Statistical one-way ANOVA analysis with the post-hoc Bonferoni test in between all time points are given in the table below

Fractional lung retention and BAL data are shown in Fig. 1a & b and in Table 3. Most [^48^V]TiO_2_-NP (fractions per IPLD) are retained in the lungs and a rather constant fraction of about 0.16 was found in the cells of BAL throughout the 28 days observation period. Free [^48^V]TiO_2_-NP in BAL fluid diminished rather quickly during the first 24 h to an IPLD fraction below 0.01. Each BAL procedure revealed alveolar macrophages (AM) as the dominating BAL cell fraction; the neutrophilic cell fraction was always below 2% (see Additional file 1: Figure S5 of the Supplementary Information).

**Table 3:** [^48^V]TiO_2_-NP retention in lungs and BAL, and in secondary organs and tissues over time p.e

	retention time (d)	1 h	4 h	24 h	7d	28d
organ or tissue	fraction of IPLD	mean ± STD	mean ± STD	mean ± STD	mean ± STD	mean ± STD
total lungs	Corr. fast clear.	0.989 ± 0.004	0.936 ± 0.023	0.918 ± 0.007	0.735 ± 0.032	0.439 ± 0.051
total lungs	Corr. resid. Blood	0.989 ± 0.004	0.935 ± 0.023	0.918 ± 0.007	0.735 ± 0.032	0.439 ± 0.051
total lungs	all corr.	0.988 ± 0.004	0.934 ± 0.023	0.91 ± 0.009	0.732 ± 0.032	0.439 ± 0.051
total lungs	fract. Conc. [1/g]	0.583 ± 0.017	0.508 ± 0.022	0.553 ± 0.013	0.372 ± 0.013	0.254 ± 0.02
total lungs (*10^1^)	mass conc. [ng/g]	6.624 ± 0.938	8.445 ± 2.779	5.281 ± 1.995	3.286 ± 0.936	3.073 ± 0.706
total lungs(*10^10^)	numb. Conc. (#/g)	1.63 ± 0.24	1.73 ± 0.73	1.43 ± 0.62	0.95 ± 0.28	0.57 ± 0.15
lav. Lungs	Corr. fast clear.	0.556 ± 0.021	0.577 ± 0.064	0.662 ± 0.033	0.555 ± 0.031	0.296 ± 0.034
lav. Lungs	Corr. resid. Blood	0.556 ± 0.021	0.576 ± 0.064	0.661 ± 0.033	0.554 ± 0.031	0.296 ± 0.153
lav. Lungs	all corr.	0.556 ± 0.021	0.556 ± 0.021	0.576 ± 0.064	0.554 ± 0.031	0.296 ± 0.034
lav. Lungs	fract. Conc. [1/g]	0.35 ± 0.012	0.333 ± 0.03	0.427 ± 0.017	0.349 ± 0.025	0.171 ± 0.014
lav. Lungs (*10^1^)	mass conc. [ng/g]	0.04 ± 0.01	0.05 ± 0.02	0.04 ± 0.01	0.02 ± 0.01	0.02 ± 0.01
lav. Lungs (*10^9^)	numb. Conc. (#/g)	9.13 ± 1.22	10.95 ± 5.97	10.17 ± 3.81	7.2 ± 2.24	3.9 ± 1.35
BALC	Corr. fast clear.	0.136 ± 0.044	0.165 ± 0.029	0.181 ± 0.027	0.175 ± 0.014	0.14 ± 0.035
BALC	Corr. resid. Blood	n.a.	n.a.	n.a.	n.a.	n.a.
BALC	all corr.	0.136 ± 0.044	0.165 ± 0.029	0.181 ± 0.027	0.174 ± 0.014	0.14 ± 0.035
BALC	fract. Conc. [1/g]	13.609 ± 4.385	16.515 ± 2.896	18.109 ± 2.743	17.437 ± 1.361	14.032 ± 3.531
BALC (*10^1^)	mass conc. [ng/g]	1.53 ± 0.51	2.51 ± 0.55	1.64 ± 0.84	1.21 ± 0.25	1.64 ± 0.3
BAL cells (*10^8^)	numb. Conc. (#/g)	3507 ± 1145.71	5065. ± 1427.	4459. ± 2527.	3518 ± 784.	3031. ± 587.
BALF	Corr. fast clear.	0.297 ± 0.035	0.194 ± 0.038	0.075 ± 0.016	0.006 ± 0.003	0.002 ± 0.002
BALF	Corr. resid. Blood	n.a.	n.a.	n.a.	n.a.	n.a.
BALF	all corr.	0.297 ± 0.035	0.194 ± 0.038	0.075 ± 0.016	0.006 ± 0.003	0.002 ± 0.002
BALF	fract. Conc. [1/g]	2.968 ± 0.348	1.937 ± 0.384	0.751 ± 0.155	0.057 ± 0.029	0.025 ± 0.016
BALF (*10^1^)	mass conc. [ng/g]	0.34 ± 0.07	0.29 ± 0.05	0.07 ± 0.04	0 ± 0	0 ± 0
BALF (*10^8^)	numb. Conc. (#/g)	779.8 ± 170.4	591. ± 159.	187. ± 109.	12.16 ± 8.82	6.37 ± 6.25
trachea (*10^− 3^)	Corr. fast clear.	13.16 ± 9.74	9.02 ± 3.48	3.06 ± 0.75	2.4 ± 1.31	1.77 ± 0.62
trachea (*10^− 3^)	Corr. resid. Blood	n.a.	n.a.	n.a.	n.a.	n.a.
trachea (*10^− 3^)	all corr.	13.16 ± 9.74	9.02 ± 3.48	3.06 ± 0.75	2.4 ± 1.31	1.77 ± 0.62
trachea (*10^− 3^)	fract. Conc. [1/g]	48.74 ± 28.69	31.22 ± 11.93	14.79 ± 6.3	5.89 ± 2.32	7.54 ± 0.98
trachea (*10^− 3^)	mass conc. [ng/g]	53.97 ± 30.95	44.47 ± 7.26	14.43 ± 10.66	3.99 ± 1.32	9.01 ± 1.55
trachea (*10^9^)	numb. Conc. (#/g)	1.23 ± 0.7	0.88 ± 0.07	0.4 ± 0.31	0.12 ± 0.04	0.25 ± 0.07
liver (*10^− 3^)	Corr. fast clear.	0.356 ± 0.173	6.032 ± 0.814	5.048 ± 0.798	5.786 ± 0.751	1.495 ± 0.225
liver (*10^− 3^)	Corr. resid. Blood	0.303 ± 0.179	5.108 ± 0.788	4.538 ± 0.753	5.675 ± 0.732	1.451 ± 0.221
liver (*10^− 3^)	all corr.	0.303 ± 0.179	5.106 ± 0.787	4.525 ± 0.75	5.617 ± 0.741	1.451 ± 0.221
liver (*10^− 4^)	fract. Conc. [1/g]	0.344 ± 0.208	5.641 ± 1.094	4.791 ± 0.855	5.835 ± 0.743	1.469 ± 0.315
liver (*10^− 2^)	mass conc. [ng/g]	3.93 ± 2.63	86.46 ± 24.29	41.08 ± 10.77	41.3 ± 11.51	17.87 ± 5.83
liver (*10^6^)	numb. Conc. (#/g)	0.9 ± 0.61	17.4 ± 5.9	11.06 ± 3.34	11.96 ± 3.46	3.29 ± 1.06
liver (*10^− 3^)	transloc. NP fract.	28.29 ± 15.2	105.01 ± 26.32	64.75 ± 6.31	44.23 ± 5.56	9.99 ± 1.12
spleen (*10^− 3^)	Corr. fast clear.	0.016 ± 0.016	0.186 ± 0.012	0.337 ± 0.085	0.581 ± 0.093	0.44 ± 0.063
spleen (*10^− 3^)	Corr. resid. Blood	0.010 ± 0.016	0.047 ± 0.022	0.251 ± 0.093	0.565 ± 0.094	0.437 ± 0.064
spleen (*10^− 3^)	all corr.	0.010 ± 0.016	0.047 ± 0.022	0.25 ± 0.093	0.565 ± 0.094	0.437 ± 0.064
spleen (*10^− 4^)	fract. Conc. [1/g]	0.099 ± 0.159	0.357 ± 0.126	2 ± 1.148	4.389 ± 0.749	7.109 ± 3.1
spleen (*10^− 2^)	mass conc. [ng/g]	0.97 ± 1.46	6.12 ± 4.24	17.02 ± 9.64	30.21 ± 5.7	83.99 ± 38.19
spleen (*10^7^)	numb. Conc. (#/g)	0.02 ± 0.03	0.13 ± 0.10	0.46 ± 0.27	0.87 ± 0.15	1.53 ± 0.65
spleen (*10^− 3^)	transloc. NP fract.	0.66 ± 0.96	1.01 ± 0.59	3.58 ± 1.22	4.44 ± 0.69	3.01 ± 0.28
kidneys (*10^− 3^)	Corr. fast clear.	0.12 ± 0.018	3.048 ± 0.949	3.642 ± 0.477	3.914 ± 0.26	1.316 ± 0.201
kidneys (*10^− 3^)	Corr. resid. Blood	0.095 ± 0.02	2.542 ± 0.91	3.426 ± 0.456	3.858 ± 0.255	1.297 ± 0.196
kidneys (*10^− 3^)	all corr.	0.095 ± 0.02	2.541 ± 0.909	3.415 ± 0.453	3.83 ± 0.255	1.297 ± 0.196
kidneys (*10^− 4^)	fract. Conc. [1/g]	0.375 ± 0.141	7.901 ± 2.109	14.376 ± 4.544	12.52 ± 1.308	4.87 ± 0.862
kidneys (*10^− 1^)	mass conc. [ng/g]	0.428 ± 0.179	12.33 ± 4.6	12.08 ± 3.34	8.78 ± 2.1	5.97 ± 2.01
kidneys (*10^6^)	numb. Conc. (#/g)	0.98 ± 0.42	24.66 ± 10.26	32.39 ± 9.36	25.39 ± 6.12	11.03 ± 3.99
kidneys (*10^− 3^)	transloc. NP fract.	9.76 ± 4.62	49.56 ± 6.3	48.97 ± 3.43	30.21 ± 2.79	8.98 ± 1.46
heart (*10^− 6^)	Corr. fast clear.	14.61 ± 3.84	239.67 ± 44.03	215.05 ± 80.53	138.94 ± 20.52	26.61 ± 12.72
heart (*10^− 6^)	Corr. resid. Blood	8.84 ± 3.8	132.79 ± 28.73	165.81 ± 79.5	127.29 ± 21.72	22.57 ± 13.15
heart (*10^− 6^)	all corr.	8.84 ± 3.8	132.78 ± 28.73	165.79 ± 79.48	127.26 ± 21.72	22.57 ± 13.15
heart (*10^− 6^)	fract. Conc. [1/g]	9.65 ± 4.43	130.84 ± 22.55	192.2 ± 104.5	132.8 ± 31.34	25.1 ± 14.84
heart (*10^− 2^)	mass conc. [ng/g]	1.13 ± 0.57	20.64 ± 7.77	15.14 ± 4.85	9.37 ± 3.41	3.28 ± 2.36
heart (*10^6^)	numb. Conc. (#/g)	0.26 ± 0.13	4.21 ± 1.88	4.03 ± 1.23	2.7 ± 0.95	0.6 ± 0.45
heart (*10^− 3^)	transloc. NP fract.	0.95 ± 0.59	2.82 ± 1.03	2.34 ± 0.98	1.01 ± 0.2	0.16 ± 0.09
brain (*10^− 6^)	Corr. fast clear.	17.28 ± 5.86	44.97 ± 11.42	49.2 ± 31.11	133.55 ± 34.6	44.6 ± 36.07
brain (*10^− 6^)	Corr. resid. Blood	15.78 ± 6.06	17.95 ± 6.31	35.88 ± 31.41	130.58 ± 34.94	43.59 ± 35.85
brain (*10^− 6^)	all corr.	15.78 ± 6.06	17.95 ± 6.31	35.88 ± 31.41	130.57 ± 34.94	43.59 ± 35.85
brain (*10^− 6^)	fract. Conc. [1/g]	8.67 ± 3.89	9.03 ± 3	19.22 ± 16.7	67.05 ± 16.98	24.56 ± 19.85
brain (*10^− 2^)	mass conc. [ng/g]	0.97 ± 0.46	1.33 ± 0.28	2.02 ± 2.5	4.5 ± 0.63	2.8 ± 1.69
brain (*10^6^)	numb. Conc. (#/g)	0.22 ± 0.11	0.26 ± 0.03	0.56 ± 0.72	1.3 ± 0.12	0.51 ± 0.29
brain (*10^− 3^)	transloc. NP fract.	1.45 ± 0.34	0.36 ± 0.1	0.53 ± 0.5	1.03 ± 0.26	0.3 ± 0.25
uterus (*10^− 3^)	Corr. fast clear.	0.011 ± 0.009	0.415 ± 0.077	0.293 ± 0.056	0.233 ± 0.027	0.136 ± 0.02
uterus (*10^− 3^)	Corr. resid. Blood	0.009 ± 0.009	0.38 ± 0.076	0.277 ± 0.055	0.229 ± 0.028	0.135 ± 0.02
uterus (*10^− 3^)	all corr.	0.009 ± 0.009	0.378 ± 0.076	0.267 ± 0.057	0.229 ± 0.028	0.135 ± 0.02
uterus (*10^− 4^)	fract. Conc. [1/g]	0.025 ± 0.027	1.012 ± 0.199	0.802 ± 0.155	0.678 ± 0.095	0.363 ± 0.061
uterus (*10^− 2^)	mass conc. [ng/g]	0.295 ± 0.336	15.968 ± 6.147	7.122 ± 1.734	4.766 ± 1.492	4.411 ± 1.246
uterus (*10^6^)	numb. Conc. (#/g)	0.088 ± 0.066	4.17 ± 1.99	1.92 ± 0.53	1.38 ± 0.43	0.81 ± 0.24
uterus (*10^− 3^)	transloc. NP fract.	0.78 ± 0.84	8.39 ± 3.8	3.83 ± 0.75	1.81 ± 0.26	0.93 ± 0.13
blood (*10^− 3^)	Corr. fast clear.	0.73 ± 0.08	13.36 ± 2.7	6.33 ± 0.66	1.38 ± 0.35	0.56 ± 0.23
blood (*10^− 3^)	Corr. resid. Blood	n.a.	n.a.	n.a.	n.a.	n.a.
blood (*10^− 3^)	all corr.	0.73 ± 0.08	13.35 ± 2.7	6.3 ± 0.65	1.38 ± 0.35	0.56 ± 0.23
blood (*10^− 4^)	fract. Conc. [1/g]	0.429 ± 0.046	7.271 ± 1.606	3.828 ± 0.406	0.818 ± 0.188	0.305 ± 0.117
blood (*10^− 1^)	mass conc. [ng/g]	0.441 ± 0.211	10.785 ± 1.137	3.347 ± 1.058	0.571 ± 0.178	0.344 ± 0.042
blood (*10^6^)	numb. Conc. (#/g)	1.11 ± 0.15	27.47 ± 5.12	9.04 ± 3.29	1.66 ± 0.56	0.63 ± 0.08
blood (*10^− 2^)	transloc. NP fract.	7.23 ± 2.4	28.41 ± 11.91	9.05 ± 0.38	1.08 ± 0.25	0.39 ± 0.16
carcass (*10^− 3^)	Corr. fast clear.	10.46 ± 3.55	43.42 ± 18.42	37.33 ± 4.83	44.03 ± 4.17	30.91 ± 2.14
carcass (*10^− 3^)	Corr. resid. Blood	10.1 ± 3.54	37.6 ± 18.45	35.45 ± 4.42	43.51 ± 4.23	30.67 ± 2.15
carcass (*10^− 3^)	all corr.	10.1 ± 3.54	37.37 ± 18.27	34.17 ± 4.14	43.39 ± 4.22	30.61 ± 2.15
carcass (*10^− 4^)	fract. Conc. [1/g]	0.49 ± 0.15	1.59 ± 0.83	1.67 ± 0.18	1.81 ± 0.26	1.39 ± 0.12
carcass (*10^− 2^)	mass conc. [ng/g]	5.38 ± 1.04	24.41 ± 11.14	14.94 ± 4.27	14.09 ± 2.25	16.95 ± 4.75
carcass (*10^6^)	numb. Conc. (#/g)	1.81 ± 0.36	6.87 ± 2.74	5.87 ± 2	5.9 ± 1.06	4.61 ± 1.49
carcass (*10^− 1^)	transloc. NP fract.	9.26 ± 0.21	7.00 ± 0.57	4.90 ± 0.24	3.03 ± 0.37	2.11 ± 0.07
skeleton (*10^− 3^)	Corr. fast clear.	0.64 ± 0.4	11.7 ± 5.53	20.84 ± 1.34	22.05 ± 3.45	22.95 ± 4.07
skeleton (*10^− 3^)	Corr. resid. Blood	0.61 ± 0.4	11.25 ± 5.54	20.71 ± 1.36	22.01 ± 3.45	22.93 ± 4.07
skeleton (*10^− 3^)	all corr.	0.61 ± 0.4	11.19 ± 5.47	20.21 ± 1.23	20.37 ± 3.51	22.9 ± 4.06
skeleton (*10^− 4^)	fract. Conc. [1/g]	0.23 ± 0.15	3.83 ± 1.99	7.73 ± 0.39	7.64 ± 1.42	7.78 ± 1.07
skeleton (*10^− 1^)	mass conc. [ng/g]	0.25 ± 0.17	5.77 ± 2.53	6.97 ± 2.47	5.81 ± 1.9	9.47 ± 2.58
skeleton (*10^6^)	numb. Conc. (#/g)	0.58 ± 0.4	11.25 ± 3.99	18.84 ± 7.67	16.75 ± 5.36	17.62 ± 5.53
skeleton (*10^− 1^)	transloc. NP fract.	0.47 ± 0.24	2.11 ± 0.17	2.92 ± 0.33	1.61 ± 0.3	1.59 ± 0.32
soft tissue (*10^− 3^)	Corr. fast clear.	9.82 ± 3.63	31.72 ± 13	16.49 ± 5.45	21.98 ± 4.14	7.96 ± 5.67
soft tissue (*10^− 3^)	Corr. resid. Blood	9.49 ± 3.63	26.24 ± 13	14.7 ± 5.07	21.48 ± 4.13	7.73 ± 5.69
soft tissue (*10^− 3^)	all corr.	9.49 ± 3.63	26.17 ± 12.94	14.48 ± 4.98	20.61 ± 3.83	7.73 ± 5.69
soft tissue (*10^− 5^)	fract. Conc. [1/g]	3.88 ± 1.37	9.92 ± 5.16	6.16 ± 2.16	8.55 ± 1.5	2.99 ± 2.23
soft tissue (*10^− 2^)	mass conc. [ng/g]	4.29 ± 1.09	15.2 ± 7.03	5.17 ± 1.38	6.03 ± 0.3	3.74 ± 2.88
soft tissue (*10^6^)	numb. Conc. (#/g)	0.98 ± 0.25	2.96 ± 1.21	1.38 ± 0.39	1.74 ± 0.09	0.68 ± 0.52
soft tissue (*10^− 1^)	transloc. NP fract.	8.79 ± 0.41	4.89 ± 0.59	2.05 ± 0.57	1.62 ± 0.26	0.53 ± 0.37
2nd organs (*10^− 3^)	Corr. fast clear.	0.53 ± 0.17	9.97 ± 1.65	9.58 ± 1.37	10.79 ± 0.93	3.46 ± 0.43
2nd organs (*10^− 3^)	Corr. resid. Blood	0.44 ± 0.17	8.26 ± 1.59	8.71 ± 1.31	10.59 ± 0.91	3.39 ± 0.42
2nd organs (*10^− 3^)	all corr.	0.44 ± 0.17	8.24 ± 1.58	8.58 ± 1.27	10.25 ± 0.98	3.39 ± 0.42
2nd organs (*10^− 4^)	fract. Conc. [1/g]	0.24 ± 0.09	4.07 ± 0.81	4.39 ± 0.78	5.22 ± 0.50	1.73 ± 0.31
2nd organs (*10^− 1^)	mass conc. [ng/g]	0.269 ± 0.117	6.294 ± 1.972	3.798 ± 0.926	3.656 ± 0.969	2.101 ± 0.617
2nd organs (*10^6^)	numb. Conc. (#/g)	0.62 ± 0.27	12.65 ± 4.67	10.22 ± 2.9	10.58 ± 2.88	3.87 ± 1.15
2nd organs (*10^− 1^)	transloc. NP fract.	0.42 ± 0.15	1.68 ± 0.35	1.23 ± 0.01	0.834 ± 0.08	0.23 ± 0.02
skin (*10^−3^)	Corr. fast clear.	1.38 ± 0.96	14.18 ± 5.83	7.97 ± 1.65	6.52 ± 0.48	3.44 ± 0.18
skin (*10^− 3^)	Corr. resid. Blood	1.29 ± 0.95	12.25 ± 5.78	7.32 ± 1.62	6.35 ± 0.44	3.35 ± 0.17
skin (*10^− 3^)	all corr.	1.29 ± 0.95	12.24 ± 5.77	7.26 ± 1.6	6.24 ± 0.46	3.35 ± 0.17
skin (*10^−5^)	fract. Conc. [1/g]	2.57 ± 1.78	22.67 ± 10.78	14.8 ± 3.6	13.32 ± 1.8	6.22 ± 0.57
skin (*10^−2^)	mass conc. [ng/g]	2.76 ± 1.69	34.83 ± 15.1	12.51 ± 2.62	9.11 ± 1.05	7.57 ± 2.01
skin (*10^6^)	numb. Conc. (#/g)	0.63 ± 0.39	8.59 ± 3.44	3.36 ± 0.86	2.63 ± 0.31	1.4 ± 0.39
skin (*1E-1)	transloc. NP fract.	1.21 ± 0.8	2.32 ± 0.62	1.04 ± 0.17	0.49 ± 0.03	0.23 ± 0.02
transloc. (*10^− 2^)	Corr. fast clear.	1.14 ± 0.36	6.44 ± 2.28	8.21 ± 0.72	12.92 ± 0.46	14.6 ± 0.74
transloc. (*10^− 2^)	Corr. resid. Blood	1.09 ± 0.36	5.32 ± 2.26	7.68 ± 0.66	12.8 ± 0.45	14.56 ± 0.75
transloc. (*10^−2^)	all corr.	1.09 ± 0.36	5.23 ± 2.19	6.95 ± 0.51	12.7 ± 0.44	14.47 ± 0.74
transloc. (*10^−4^)	fract. Conc. [1/g]	0.5 ± 0.14	2.14 ± 0.96	3.18 ± 0.23	5.69 ± 0.24	6.22 ± 0.47
transloc. (*10^− 1^)	mass conc. [ng/g]	0.006 ± 0.001	0.033 ± 0.014	0.031 ± 0.01	0.04 ± 0.007	0.077 ± 0.022
transloc. (*10^6^)	numb. Conc. (#/g)	1.28 ± 0.23	6.52 ± 2.44	8.26 ± 3.04	11.5 ± 2.25	14.2 ± 4.42

n.a. “Correction for residual blood is not applicable”

The data are presented as retained [^48^V]TiO_2_-NP fractions of IPLD (applied correction for fast clearance by MCC (corr. Fast clear.)) in each first line of a given organ. In each second line the data were corrected for the [^48^V]TiO_2_-NP content in residual blood after exsanguination (corr. Resid. blood) and in each third line the data were further corrected for ^48^V-activity which can be attributed to free, ionic ^48^V that was leaching out of the [^48^V]TiO_2_-NP (all corr.) following the procedure in the Additional file 1, section 14, and described previously in [29]. After these corrections, the ^48^V-activity data were converted into [^48^V]TiO_2_-NP concentrations per mass of organ or tissue, given either as fractions per gram (g^− 1^) or as mass concentrations (in ng•g^− 1^) or as number concentrations (in #•g^− 1^). In the seventh line of each secondary organ or tissue, the fractions are normalized to the total fraction of those [^48^V]TiO_2_-NP which had crossed the ABB up to the given dissection time point (transloc. NP fract., see Eq. (24) of the Additional file 1). Mean ± SEM of *n* = 4 rats/time point. Note the varying exponents in brackets of the left column are used to ensure an adequate number of valid digits. Graphical displays of data of lines 3, 4 and 7 of each organ or tissue are shown in Figs. 1, 4, 5 and Additional file 1: Figure S6 of Supplementary Material, respectively

The ranking order for all secondary organs, blood, skeleton, and soft tissues at 28d p.e. according to each third line “all corr.” and each seventh line “transloc. NP fract.” is:

skeleton > soft tissue > liver > kidneys > blood > spleen > uterus > brain > heart

However, this ranking order changes for the lines “fract. Conc.” for “mass conc.” and “numb. Conc.” per mass of organ or tissue to:

skeleton > spleen > kidneys > liver > uterus > soft tissue > blood > heart > brain

A least-squares fit of the mean alveolar [^48^V]TiO_2_-NP retention of each group of rats over 28 days p.e. (taken from Table 3, total lungs) to a mono-exponential decrease yields a retention half-life of 25 days. For comparison, when fitting the body retention data derived from excretion data between 3 and 28 days to a bi-exponential fit, retention half-lives of 6 and 38 days were obtained, respectively (see Additional file 1: Figure S1 in the Supplementary Information). These values are much shorter than the 60–70 days half-life frequently reported in the literature. [26, 32, 33] The difference can be attributed to three factors [32, 34]. Firstly, the lung fits are based on only three lung retention data points at 24 h, 7 days and 28 days p.e. Secondly, the studied retention period is too short to correctly determine the long-term retention half-life because the mono-exponential fit is only a first-order attempt for a retention pattern which continuously declines over time resulting in an increasing half-life. We made a similar observation in our analysis of lung retention data up to 168 days after the intratracheal inhalation of 20 nm IrNP when compared to the first 30 days; for 168 days we obtained half-lives of 6 and 160 days versus 3 and 25 days for the first 30 days p.e. (see Additional file 1). Thirdly, the body retention data in the present study, represent predominantly lung retention and minor contributions of the translocated fractions retained in all secondary organs, tissues, and blood (see Table 3 and Additional file 1: Figure S1 of the Supplementary Material). The excretion data collected over a period of 28 days according to Eq. (1b) of the Additional file 1 confirm continuously decreasing excretion rates, giving rise to an increased body retention half-life of 38 days as detailed in the Additional file 1. The use of radiolabeled [^48^V]TiO_2_-NP allows us to precisely control the nanoparticle dose which is effectively deposited in the lungs and its redistribution in the whole body of the animals, including fecal and urinary excretions.

### Long-term macrophage-mediated [^48^V]TiO_2_-NP clearance

**Fig. 2:**
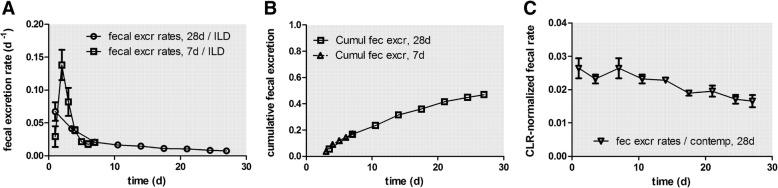
Daily fecal excretion as ^48^V fractions of intratracheally inhaled [^48^V]TiO_2_-NP. Panel **a**: Daily fecal excretion of intratracheally inhaled [^48^V]TiO_2_-NP normalized to the total deposited [^48^V]TiO_2_-NP lung dose (ILD) present in the lungs 2 h p.e., representing MCC from the conducting thoracic airways during the first two days after inhalation and thereafter LT-MC from the alveolar region. The mean ILD in mass (number) of [^48^V]TiO_2_-NP of all five retention time points is 1.36 ± 0.45 μg (3.59 ± 1.08•10^10^ #). Data of the groups of rats analyzed 7 days and 28 days p.e. are presented. For the 28 days group, fecal excretions sampled over 3–4 days are divided by the number of sampling days and associated with the mean day of the sampling period. Panel **b**: Cumulative fecal excretion without MCC of the 7 days and 28 days group. Panel **c**: The excretion rates are normalized to contemporary lung retention (CLR) at each dissection time point. The CLR was obtained from an exponential fit to the lung fractions (sum of lavaged lungs, BALF and BALC) at each dissection time point (see Eq. (1a) and (1c) of the Additional file 1). Mean ± SEM, n = 4 rats per time point

Nano-sized and micron-sized particles retained in the peripheral lungs are continuously cleared by LT-MC towards ciliated airways and the larynx and are subsequently swallowed into the GIT. Since only, a very small percentage (see the end of Discussion) of the NP will be absorbed across the gut walls, and taking into account the chemical stability of TiO_2_-NP that prevents their efficient dissolution, nearly all the swallowed [^48^V]TiO_2_-NP will be excreted in feces [29]. Therefore, fecal [^48^V]TiO_2_-NP excretion after day 3 p.e. corresponds to NP cleared from the alveolar region, since the hepato-biliary particle clearance (HBC) pathway to fecal excretion can be neglected in the present case because (a) only a minor fraction of particles accumulate in the liver (see Table 3) and (b) an even much lower fraction of the particles retained in the liver is cleared via HBC [28, 29]. However, immediately after particle deposition in the thoracic region of the respiratory tract, LT-MC is superimposed by MCC of [^48^V]TiO_2_-NP from the ciliated, conducting airways as shown in Table 3 and Fig. 2. The fecal excretion of the MCC contribution is concluded after 1–3 days p.e., which corresponds to the time required for the GIT passage of the [^48^V]TiO_2_-NP cleared by MCC. After this period the daily fecal excretion is dominated by LT-MC and the clearing rate from the peripheral lungs declines from 0.06 to 0.01 d^− 1^ of IPLD. Figure 2a shows that the daily fecal excretion including MCC exhibits a delayed maximum at 2 days p.e. When the fractional fecal [^48^V]TiO_2_-NP excretion rates are normalized to the contemporary [^48^V]TiO_2_-NP lung retention in Fig. 2b, these rates (down triangles), agree rather well with the predicted rates of 0.02–0.03/d obtained for micron-sized particles [35] as well as those obtained for 20 nm [^192^Ir] IrNP [26] and 20 nm [^195^Au] AuNP [23].

**Fig. 3:**
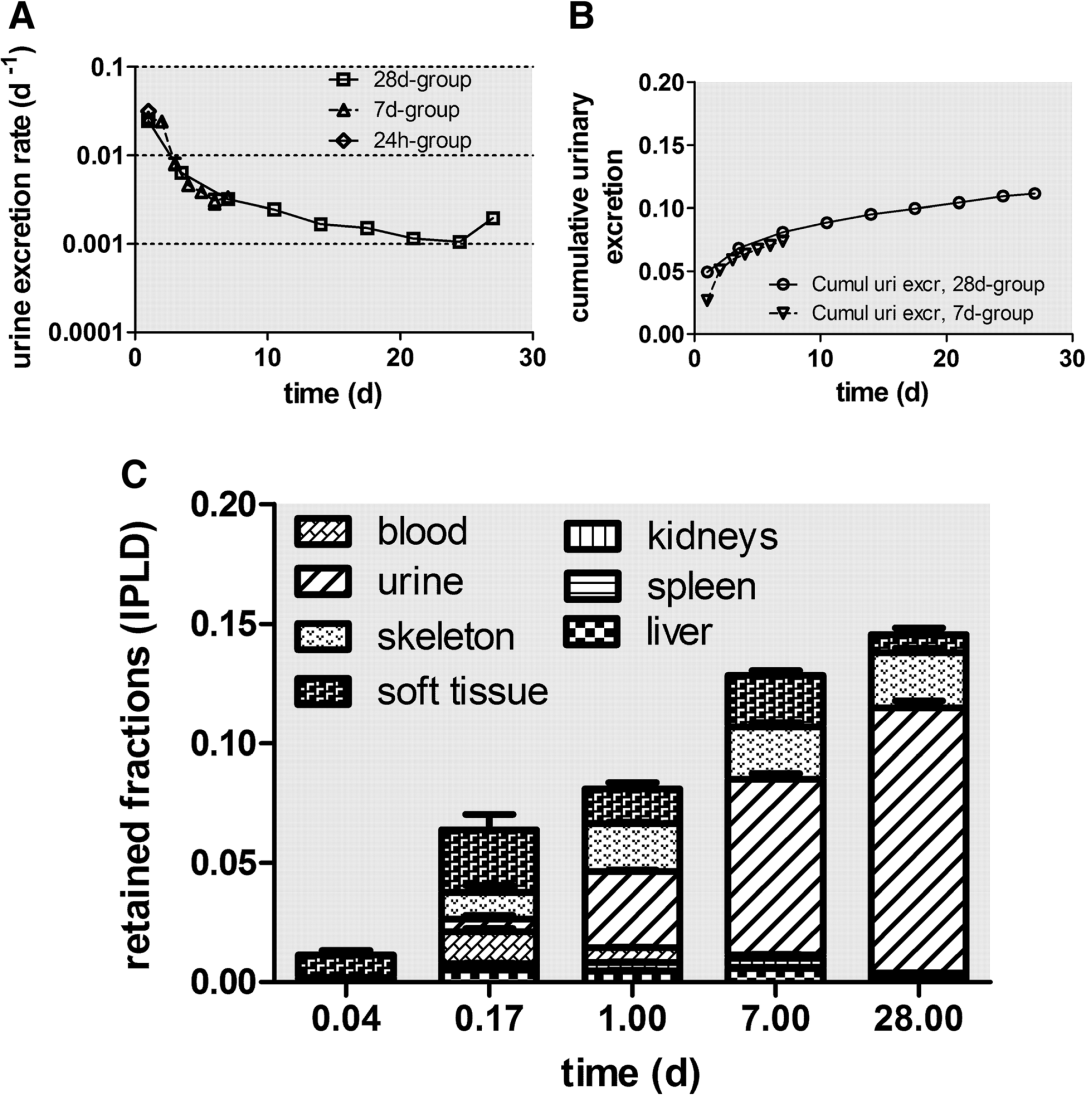
Daily urinary excretion as 48V fractions of intratracheally inhaled [48V]TiO2-NP. Panels **a** + **b**: Daily urinary excretion as 48V fractions of intratracheally inhaled [48V]TiO2-NP of the 7 days and 28 days group. Data are given as fractions of IPLD. The mean IPLD in mass (number) of [48V]TiO2-NP of all five retention time points is 1.01 ± 0.55 μg (2.67 ± 1.16•1010 #). For the 28 days group, the urine sampled over 3–4 days are divided by the number of sampling days and associated with the mean day of the sampling period. Mean ± SEM, n = 4 rats per time point. Panel **c**: Translocated [48V]TiO2-NP across the ABB presented as stacked columns with accumulation in major secondary organs, soft tissue, skeleton, and cumulative urine. While translocated [48V]TiO2-NP accumulated predominantly in soft tissue up to 24 h the fraction released in urine increases rapidly from day 7 to day 28 p.e. Mean ± SEM, n = 4 rats per time point

In Fig. 3a+b the daily urinary excretion is plotted, based on the data retrieved from the rats of the 7 days and the 28 days group. These agree very well with each other. During the first 3 days, the urinary excretion rates amount to 0.03 d^− 1^ but drop sharply below 0.01 d^− 1^ and then decline gradually to 0.001 d^− 1^ at day 28 (Fig. 3a). Figure 3b shows clearly that the cumulative urinary excretion is much smaller than the cumulative fecal excretion in Fig. 2b. After 28 days about 1 μg of the initially deposited lung dose of (1.37 ± 0.39) μg were excreted via feces while less than 0.15 μg were excreted via urine, which emphasizes the prominent role of the initial MCC and of LT-MC after day 3.

### Biokinetics of translocated [^48^V]TiO_2_-NP into secondary organs and tissues

In Table 3 the retention of [^48^V]TiO_2_-NP in the lungs and in all secondary organs and tissues is presented as mean ± SEM for all dissection time points 1 h, 4 h, 24 h, 7d, and 28d p.e. The data are given as fractions and mass concentrations of the IPLD, which is the total deposited lung dose corrected for the [^48^V]TiO_2_-NP fraction which is rapidly cleared from the conducting lung airways by MCC applying the mathematical procedure described in the Additional file 1. Since the ^48^V-radiolabel is extrinsic to the titania NP matrix we corrected for the release of non-particulate (ionic) ^48^V from the TiO_2_-NP, using as a basis the biokinetics of ^48^V ions after IT instillation of soluble ^48^V nitrate as described previously in [29]. The effect of ionic ^48^V-release is the difference between line 2 (corr. Resid. blood) and line 3 (all corr) for each organ or tissue; in most of the data, the difference is extremely small, indicating that the ^48^V radiotracer is firmly integrated into the [^48^V]TiO_2_-NP matrix.

**Fig. 4:**
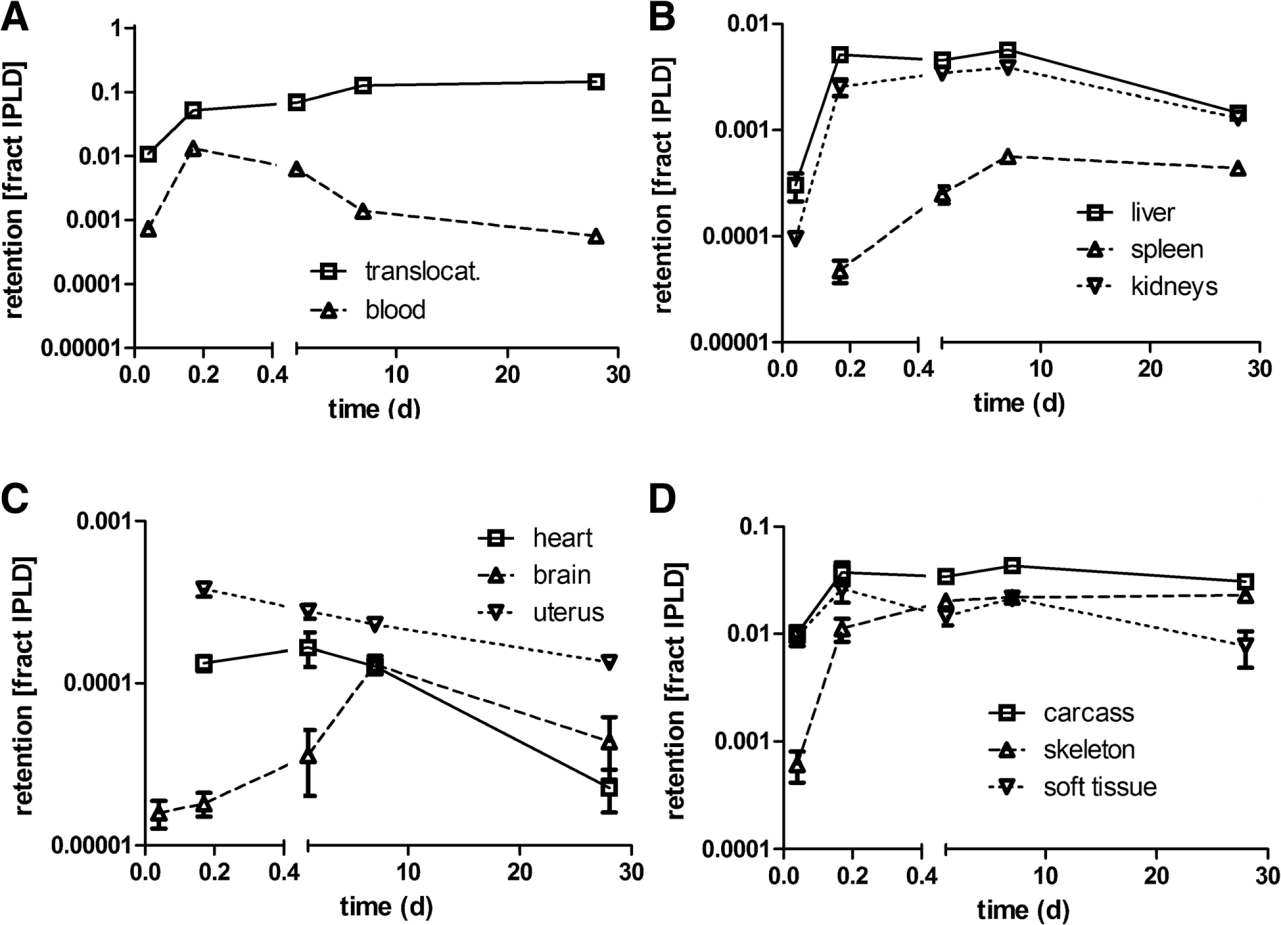
Translocated and retained fractions of intratracheally inhaled [^48^V]TiO_2_-NP in the organism. Retained fractions of intratracheally inhaled [^48^V]TiO_2_-NP investigated up to 28 days p.e. **a**: total translocation and blood; **b**: liver, spleen, and kidneys; **c**: heart, brain, and uterus (at 1 h p.e. heart and uterus fractions were < 0.00001); **d**: carcass, soft tissue, and skeleton. The mean IPLD in mass (number) of [^48^V]TiO_2_-NP of all five retention time points is 1.01 ± 0.55 μg (2.67 ± 1.16•10^10^ #). [^48^V]TiO_2_-NP retention is given as fractions of the initial peripheral lung dose (IPLD). Data in all panels correspond to the third line (all corr.) of each organ in Table 3. Mean ± SEM, n = 4 rats per time point. Statistical one-way ANOVA analysis with the post-hoc Bonferoni test in between all time points are given in the table below:

Figure 4 displays the fully corrected fractions per IPLD of Table 3. Immediately after inhalation a rapid translocation into blood is observed followed by fast uptake in soft tissue, which saturates thereafter. The increasing [^48^V]TiO_2_-NP content in blood (Fig. 4a) leads to steep increases in liver, kidneys, heart, uterus, and skeleton during the first 4 hours p.e. (Fig. 4b-d). However, while [^48^V]TiO_2_-NP content in the blood drops down to the initial value at 1 h p.e. the [^48^V]TiO_2_-NP content in those organs and skeleton remain rather constant over the entire observation period. The [^48^V]TiO_2_-NP contents in liver and kidneys reach almost 1% of IPLD while those in heart and uterus are smaller than 0.1% of IPLD. The [^48^V]TiO_2_-NP contents in soft tissue and skeleton reach even higher values of 2–3% of IPLD. While the accumulation of [^48^V]TiO_2_-NP in soft tissue proceeds rapidly within 4 h p.e. the accumulation in the skeleton is slower but retention is more persistent until the end of the study. The declining data of soft tissue between day 7–28 p.e indicates clearance processes. The total fraction of [^48^V]TiO_2_-NP translocated across the ABB reaches nearly 15% of IPLD 28 days p.e. (Fig. 4a) which is tenfold more than the ABB-translocation of same-sized [^195^Au] AuNP after their intratracheal inhalation [23].

### [^48^V]TiO_2_-NP concentration per mass of organ or tissues (1/g) as fractions of the initial peripheral lung dose (IPLD)

**Fig. 5:**
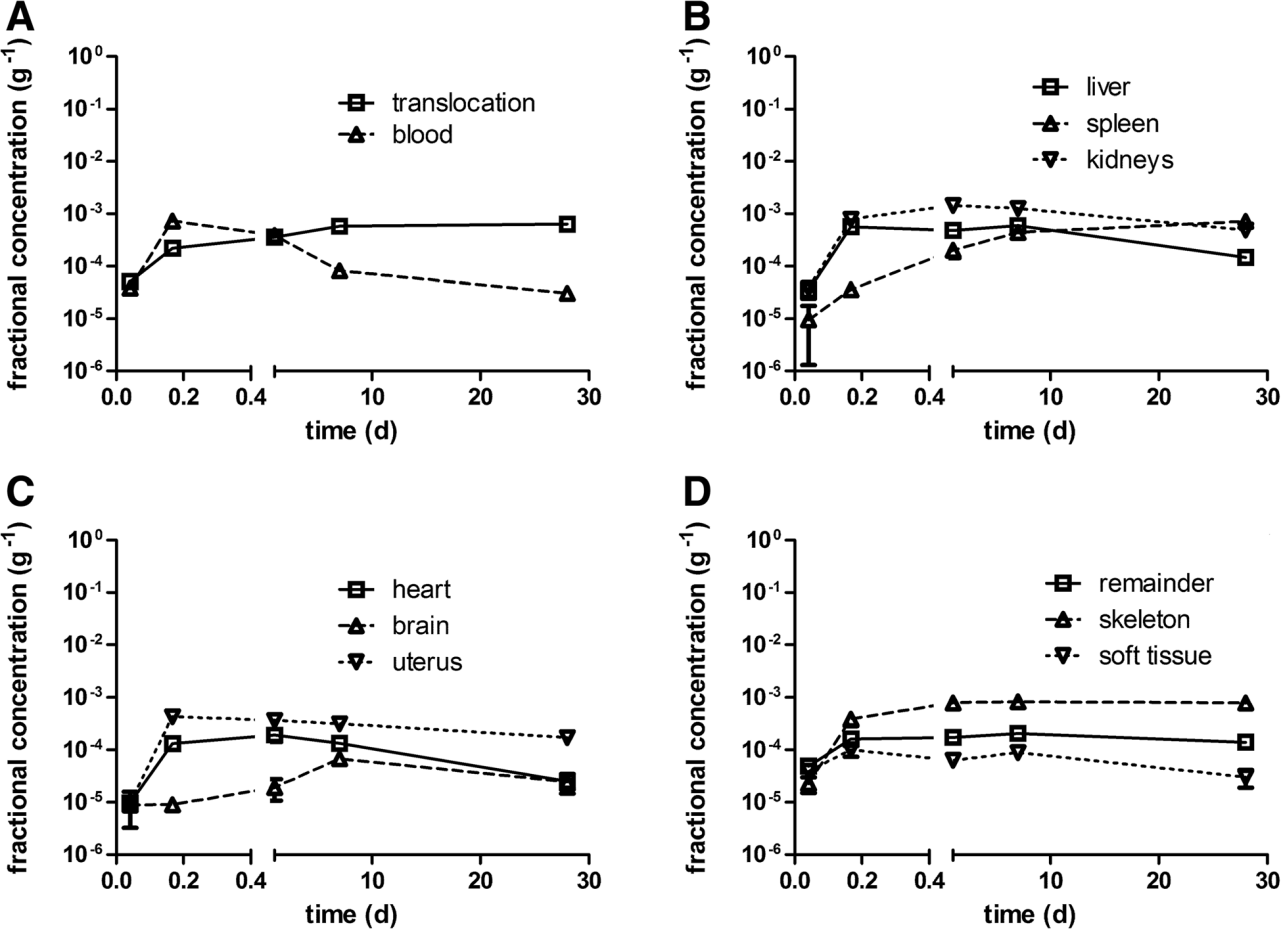
Kinetics of [^48^V]TiO_2_-NP concentrations per weight of organ or tissue: (**a**): total translocation and blood, (**b**): liver, spleen, and kidneys, (**c**): heart, uterus, and brain, (**d**): carcass, skeleton, and soft tissue. The mean IPLD in mass (number) of [^48^V]TiO_2_-NP of all five retention time points is 1.01 ± 0.55 μg (2.67 ± 1.16•10^10^ #). Data are corrected for [^48^V]TiO_2_-NP retained in the residual blood volume of organs and tissues; data are presented as mean ± SEM; n = 4 rats per time point. Statistical one-way ANOVA analysis with the post-hoc Bonferoni test in between all time points are given in the table below:

In Fig. 5 we present the [^48^V]TiO_2_-NP fractions per weight of organ or tissue for toxicological considerations. 1 h p.e. the [^48^V]TiO_2_-NP concentration fractions (per organ weight) in blood and in all secondary organs start rather low at about 1 × 10^− 5^ g^− 1^ (Fig. 5a-c and Table 3). Fig. 5a shows a tenfold increase of concentration in blood during the next 3 hours followed by a gradual decrease to a constant value from day 7 to day 28 which is close to the initial concentration determined at 1 h p.e. The initially steep increase was also found in liver, kidneys, heart, and uterus indicating rapid uptake by the MPS cells of these secondary organs when the blood concentration is still high. While the blood concentration starts to decrease 4 h p.e. and reaches a value close to that immediately after exposure, the concentrations in the aforementioned organs decreased only very little between 7 and 28 days p.e. The spleen shows a different behavior characterized by a slower but persistent increase by almost two orders of magnitude over 28 days suggesting continuous uptake by its MPS cells independent of blood concentrations.

[^48^V]TiO_2_-NP concentrations in soft tissue and skeleton (Fig. 5d and Table 3) start similarly low as in blood and secondary organs. Due to the large mass of soft tissue the increase is modest during the next 3 hours and follows the declining [^48^V]TiO_2_-NP concentration pattern in blood. In contrast, the concentration in the skeleton rises tenfold during the next 3 hours and stays rather constant during the whole follow-up period up to 28 days p.e., similar to the MPS mediated biokinetic patterns observed in liver, kidneys, heart, and uterus.

### Corrections for residual blood in secondary organs and tissues

In order to obtain the true value of ^48^V activity in the organs and tissues of interest, the radioactivity of the residual blood retained after exsanguination had to be subtracted. The derivation of this correction (i.e. the ratio RB of ^48^V-activity in the residual blood normalized to the totally measured ^48^V-activity retained in a given organ) is given in the Additional file 1 (see Eq. 23). The ratios RB presented in Additional file 1: Figure S5 of the Supplementary Information are larger than 0.1 during the first 24-h p.e. for most of the secondary organs and somewhat smaller in soft tissue and skeleton. This may have resulted from either early ionic release of the ^48^V radiolabel (which will be discussed later) or from initially rather high amounts of [^48^V]TiO_2_-NP translocated through the ABB or both. The latter would cause high concentrations of [^48^V]TiO_2_-NP circulating in the blood during the first 24 h p.e. and retarded accumulations in secondary organs, soft tissue, and skeleton. However, the corrections are very small in the (lavaged) lungs since the dominant part of the [^48^V]TiO_2_-NP was retained in the lungs and the ^48^V activity concentration in blood was very low.

## Discussion

Due to the design of the present study, we could not focus on the TiO_2_-NP biokinetics at a microscopic level. However, using the same spark ignition TiO_2_-NP generation technology and similar inhalation methods, we have previously studied the microscopic fate of 20 nm-sized TiO_2_-NP retained in the lungs of rats during the first 24 h p.e. using transmission electron microscopy (TEM). [36–38]. TiO_2_-NP were found on the lung epithelium either as free luminal TiO_2_-NP (mostly immediately p.e.), as well as in alveolar macrophages (AM) and in alveolar epithelial Type I cells (Ep1) and Type 2 cells (Ep2). Much smaller numbers of TiO_2_-NP were found in vascular endothelia. Most intracellular TiO_2_-NP were retained in vesicles of all three cell types. Initially individual and small agglomerates of TiO_2_-NP were observed in the cells but with increasing time larger TiO_2_-NP agglomerates were observed indicating ongoing agglomeration inside the cells.

**Fig. 6:**
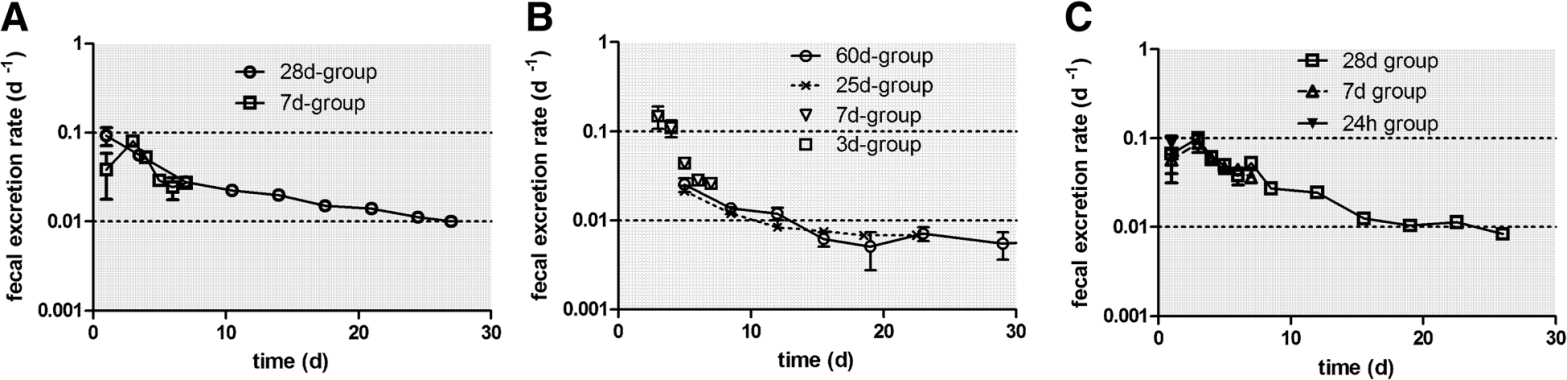
Comparison of daily fecal excretion for three types of inhaled, 20-nm sized nanoparticles. Mean daily fecal excretion (± SEM) for n = 4 rats of each group analyzed at different retention days. **a**: [^48^V]TiO_2_-NP; **b**: [^192^Ir]IrNP; **c**: [^195^Au]AuNP

The long-term [^48^V]TiO_2_-NP lung retention is very similar to that after the inhalation of 20-nm sized [^192^Ir] IrNP and [^195^Au] AuNP [23–26] independent of the NP material. It is mainly determined by long-term macrophage-mediated clearance (LT-MC) between 3 and 28 days p.e., as can be concluded from Fig. 6, which shows the daily fecal excretion rates normalized to IPLD.

**Fig. 7:**
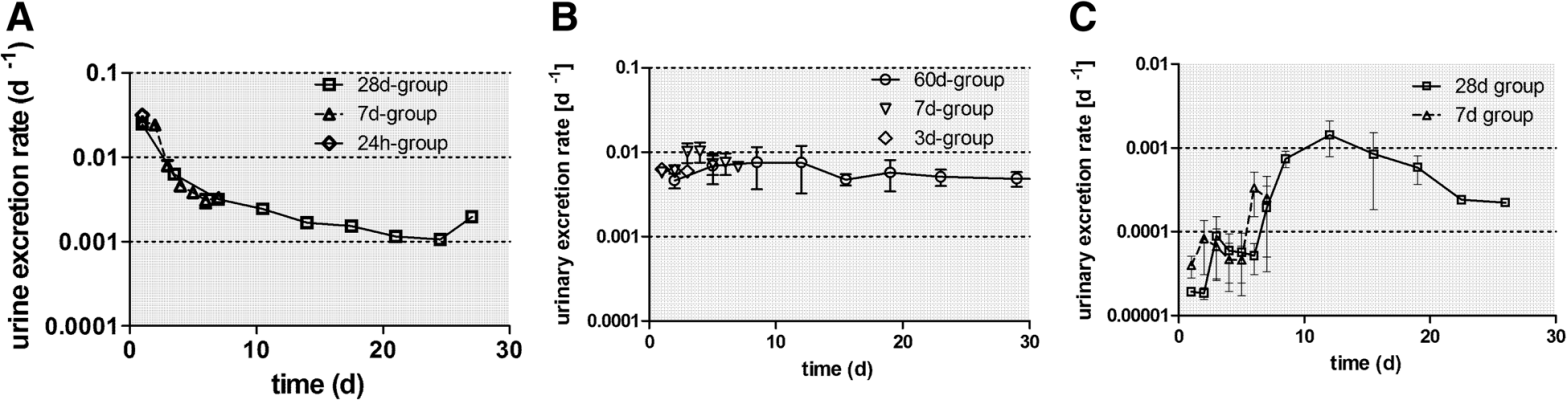
Comparison of the daily urinary excretion for three types of inhaled, 20-nm sized nanoparticles. Mean daily urinary excretion (± SEM) for n = 4 rats of each group analyzed at different retention days. **a**: [^48^V]TiO_2_-NP; **b**: [^192^Ir]IrNP; **c**: [^195^Au]AuNP

However, the urinary excretion differs consistently between the three NP as shown in Fig. 7. While [^192^Ir] IrNP are excreted in urine with a constant rate of 0.01 d^− 1^ per IPLD throughout the entire observation period up to 28 days p.e., [^48^V]TiO_2_-NP excretion starts with a rate of 0.03 d^− 1^ during the first two days p.e. and declines tenfold to 0.003 d^− 1^ at day 5 p.e. Thereafter, the rates drop to 0.001 d^− 1^ up to 28 days p.e. In contrast, the excretions rates of [^195^Au] AuNP are < 0.0001 d^− 1^ during the first week p.e., but increase tenfold thereafter with a maximum at 12 days p.e. In order to be excreted via urine, the NP must have entered the circulation and have been filtered from the blood by the renal glomeruli of the kidneys into the urine. Urinary NP excretion has been shown by several publications to occur only for NP smaller than 6–8 nm [39–42]. This would imply disagglomeration of the originally inhaled 20 nm NP before, during or after passage across the ABB. Alternatively; the ^48^V, ^192^Ir or ^195^Au radiolabels were released from their respective NP matrix into ionic form. Both options will be discussed below.

**Fig. 8:**
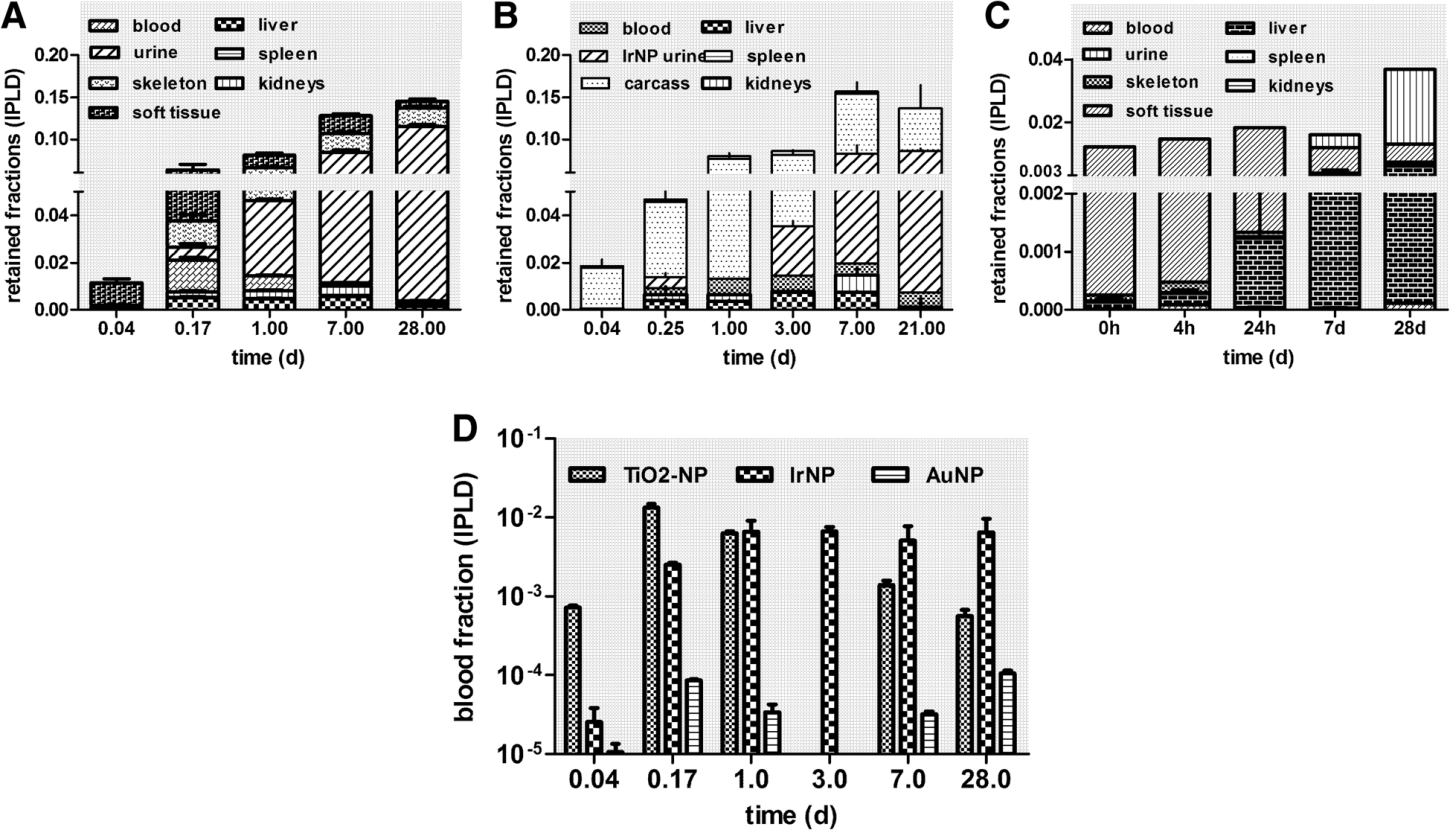
Stacked retention fractions for three types of inhaled, 20-nm sized nanoparticles at each time point. Stacked fractions of three different NP in various secondary organs, tissues, blood, and cumulative excretion in urine over time p.e., respectively. **a**: [^48^V]TiO_2_-NP; **b**: [^192^Ir]Ir-NP; **c**: [^195^Au]AuNP. Panel **d**: blood concentrations of all three NP types over time p.e

To further analyze the translocation across the ABB, which results either in urinary excretion or in NP accumulation in secondary organs and tissues, Fig. 8a-c shows these fractions as stacked columns for each of the three NP types at each dissection time point. Notably, 28 days p.e. the integral translocation for [^195^Au] AuNP is only a quarter of those of [^48^V]TiO_2_-NP or [^192^Ir]IrNP. Immediately after inhalation (1 h), accumulation in soft tissue is dominant for all three NP types and remains dominant for [^192^Ir] IrNP and [^195^Au] AuNP during the first 24 h; thereafter soft tissue fractions decline due to effective NP clearance. Fig. 8d shows blood fractions of all three NP types; [^195^Au] AuNP fractions are at least tenfold lower than those of the other two NP types. Notably, blood fractions for all NP types steeply increase during the first 4 h p.e. illustrating the increase of NP translocation across the ABB to blood. After 4 h p.e. blood fractions for both [^192^Ir] IrNP and [^195^Au] AuNP remain rather constant over the entire period; of note is the fact that the latter is 100-fold lower than the former. These constant patterns indicate a stable balance between NP eliminated from blood and released into blood. In contrast, [^48^V]TiO_2_-NP blood fractions show a maximum at 4 h p.e. and decline thereafter more than tenfold. This decline is accompanied by increasing urinary excretion over time (Fig. 8a) eliminating most of the translocated [^48^V]TiO_2_-NP from the organism. [^192^Ir] IrNP urinary excretion starts later at day 3 p.e. and eliminates similarly most of the [^192^Ir] IrNP from the organism (Fig. 8b). Urinary [^195^Au] AuNP excretion starts only at day 7 p.e. and is less prominent than those of the other NP. Instead, the liver MPS removes efficiently [^195^Au] AuNP from blood starting from 4 h p.e. resulting in liver fractions that are larger than all other fractions at days 7 until 28 p.e. (Fig. 8c). In contrast, relative to the total translocated NP types, liver fractions of the other two NP types remain very low.

### The release of ^48^V-ions from [^48^V]TiO_2_-NP

The ^48^V-radiolabel appearance in urine during the first 24 h and its increase thereafter suggests ionic release from the chemically different [^48^V]TiO_2_-NP matrix. However, our biokinetics control study after IT-instillation of soluble ^48^V-nitrate (see Additional file 1: Figure S7 of the Supplementary Information which was presented initially in the Additional file 1 of our recent report [29]) do not support the hypothesis of ionic release, since 7 days p.e. neither skeletal fractions nor those of liver and kidneys are as high as expected for ^48^V-ions. One would expect three or even ten times higher ^48^V activity fractions in urinary excretions, respectively, at the corresponding time points p.e. (see Additional file 1: Figure S7 of the Supplementary Information). Also, our biokinetics studies after the inhalation or instillation of soluble nitrates of ^192^Ir and ^195^Au do not support the dissolution hypothesis since the biokinetics patterns of the control studies with soluble nitrates are not comparable with those after the inhalation of the two NP types. The fact that urinary excretion is minimal during the first 24 h p.e. - while translocated ^48^V, ^192^Ir or ^195^Au starts accumulating in soft tissue - is not consistent with the rapid urinary excretion of soluble forms of ^48^V, ^192^Ir or ^195^Au after release from the respective NP matrix observed in biokinetics control studies using soluble ^48^V [29], ^192^Ir [24] or ^195^Au [23]. Since any ionic release from the NP matrix might be expected to occur directly after inhalation and deposition of the NP in the lungs, this finding strongly opposes the occurrence of soluble forms of ^48^V, ^192^Ir or ^195^Au in our studies. It is noteworthy that in the case of no detectable ^48^V-ions release from the [^48^V]TiO_2_-NP, the assumption – the entire ^48^V activity in urine is ionic – used for corrections in lines 3 of each organ (Table 3) is too conservative and the corrections for ionic release diminish completely.

In contrast, disagglomeration of the retained NP in the lungs into primary particles and/or smaller sized agglomerates/aggregates is likely since the morphologic structure of [^48^V]TiO_2_-NP and of [^192^Ir] IrNP shows delicate chain agglomerates/aggregates of primary particles of 2–5 nm caused by the evaporation/condensation during spark ignition aerosol generation [24, 43]. As we have discussed recently in our report on the biokinetics of [^195^Au] AuNP [23], disagglomeration may also take place, at least to some extent, for the spark ignition generated AuNP aerosol. If the disagglomerated primary particles and/or smaller sized agglomerates/aggregates are below 6–8 nm their translocation probability across the ABB into blood increases rapidly and there is sufficient evidence in the literature that these disagglomerates can pass renal glomerular filtration into the urine [39–42].

Since we may rule out significant urinary excretion of ionic ^48^V, ^192^Ir or ^195^Au, the question arises of how disagglomeration may have occurred? Physiologically it is unlikely that disagglomeration occurs in the epithelial lining fluid, but it occurs inside the involved cells, i.e. epithelial type 1 and type 2 cells, AM and macrophages in the interstitium maturing from arriving blood monocytes. Fig. 8 shows already a fast translocated [^48^V]TiO_2_-NP fraction of 0.009 immediately after the 2 h inhalation; this fast translocation is more likely to occur for disagglomerated primary particles and/or smaller sized agglomerates/aggregates compared to the inhaled 20 nm [^48^V]TiO_2_-NP. The translocated fraction is predominantly retained in soft tissue – most likely in tissue phagocytes. The translocated fraction in soft tissue increases threefold during the next 4 h p.e. to 0.026, in addition to translocated fractions appearing in the skeleton (0.011), blood (0.013), liver (0.005), and also in urine (0.004). However, 24 h after inhalation the fraction in soft tissues decreases twofold to 0.014 indicating rapid elimination. Due to this rapid elimination, it appears plausible that additional disagglomeration may occur in soft tissue adding to the increased blood concentration of disagglomerated particles (DP) while disagglomeration likely continues in the lungs leading to continued ABB translocation. However, from these biokinetics data, we cannot exclude further disagglomeration and release to blood from other secondary organs. The increasing urinary fraction from 4 h to 24 h (0.032) suggests that an increasing part of DP is below 6–8 nm. In this regard urinary excretion and hence urinary DP excretion after [^48^V]TiO_2_-NP inhalation becomes already detectable 24 h p.e. and continues to increase to 0.073 (7d) and to 0.11 (28d) (see Fig. 8). It is noteworthy that this disagglomeration depends on the specific physico-chemistry of primary particle formation in the electrical discharge, since urinary DP excretion after [^192^Ir] IrNP inhalation shows different kinetics and becomes visible only 3 days p.e. (Fig. 8) and urinary DP excretion after [^195^Au] AuNP inhalation is delayed even longer and starts at 7 days p.e. The primary particles (PP) of [^48^V]TiO_2_-NP and of [^192^Ir] IrNP are in the range between 2 and 5 nm [24, 43] and those of the [^195^Au] AuNP are 6–8 nm due to the generation process after spark ignition. While the former two materials condense instantaneously and form solid clusters of [^48^V]TiO_2_-NP and of [^192^Ir] IrNP which aggregate and agglomerate to the final aerosol-dilution controlled size of 20 nm chain aggregated particles [24, 43], [^195^Au] AuNP remain liquid initially in the spark area and coalesce to tiny droplets of 6–8 nm in the hot zone around the spark before they become cold enough to solidify [44]. With regard to the different primary particle sizes of the three types of NP, it appears reasonable that (assuming disagglomeration) cumulative urinary DP excretion of [^48^V]TiO_2_-NP and of [^192^Ir] IrNP would start faster p.e. and their excreted fractions of about 0.1 per IPLD would be larger than the excreted 0.02 DP fraction (per IPLD) of [^195^Au]AuNP.

### NP relocation into the interstitium and re-entrainment back onto the epithelial surface

At the low dose of NP inhaled over 2 hours in our experiments we emphasize that there were no signs of inflammation, which could be revealed by inflammatory cells in BAL (see Additional file 1: Figure S4).

**Fig. 9:**
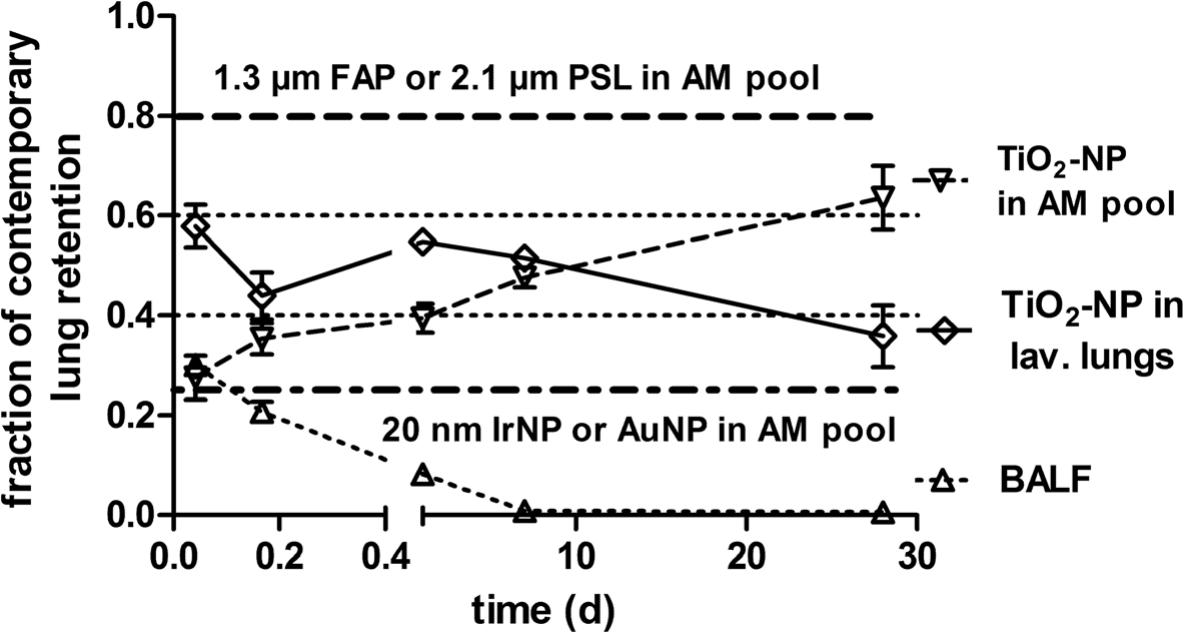
Comparison of interstitial relocation and epithelial re-entrainment for nano- and micron-sized particles. Estimated [^48^V]TiO_2_-NP retention fractions for the total lung surface macrophage pool [26] (for the definition of the AM pool, see the Additional file 1) derived from the experimentally determined kinetics of lavageable [^48^V]TiO_2_-NP fractions associated with BALC (downward triangles). Additionally, the [^48^V]TiO_2_-NP fractions found in the interstitium, estimated from [^48^V]TiO_2_-NP fractions of lavaged lungs (diamonds) are shown. Fractions are normalized to the contemporary lung burden at 1 h to 28 days after intratracheal inhalation. Additionally, the fractions of free [^48^V]TiO_2_-NP in BALF are presented, which rapidly decline p.e. (upward triangles). These data are compared with averaged retention fractions for 20 nm [^192^Ir] IrNP and [^195^Au] AuNP in the total AM pool [23, 25, 26] and with those of 1.3 μm fused aluminum-silicate particles (FAP) particles and 2.1 μm polystyrene (PSL) particles in the total AM pool [45, 46]. Data points are mean ± SEM; n = 4 rats per time point

According to Fig. 1, we observed that only a small fraction (0.2) of BAL-cell associated [^48^V]TiO_2_-NP is lavageable which gradually increases throughout the entire study period; this finding contrasts with that observed for inhaled micron-sized particles (μP) as shown by [45–47] where μP fractions of 0.8 (relative to the contemporary lung burden, CLR) were BAL-cell associated and constantly lavageable during 6 months of retention p.e. as shown by the dashed line in Fig. 9.

Immediately after inhalation (i.e. on average 1-h p.e.) and at 4 h p.e., a major fraction of [^48^V]TiO_2_-NP may not be lavageable since they either may have adhered to the luminal membranes of epithelial cells of type 1 (Ep1) or type 2 (Ep2) or the NP may have already been endocytosed by these cells.

Yet, as long as there were free [^48^V]TiO_2_-NP on the epithelium, we proved their lavagebility in BALF immediately after inhalation and 4 h p.e., (Fig. 1 and 9). After this, from 24 h p.e. until the end of the study free [^48^V]TiO_2_-NP had disappeared from the epithelium and hence, the lavaged fractions of free [^48^V]TiO_2_-NP became negligible (< 0.01). This observation agrees well with similar fractions of free 20-nm-sized AuNP and IrNP in BAL (0.2) directly after inhalation and 4 h p.e. showing that same-sized NP of other materials can also be lavaged from the lung surface as free NP [23, 25, 26] Since a few hours after deposition continued adherence on the luminal membranes of epithelial cells is unlikely, the NP are assumed to be endocytosed by Ep1 and Ep2 thereafter. This is consistent with morphometric lung analyses (electron-microscopy including elemental mapping at the Anatomical Institute in Bern, Switzerland) performed on the same strain of rats which had inhaled the physico-chemically equal, but not radio-labeled 20-nm-sized TiO_2_-NP aerosol for which we had used our aerosol generation and inhalation equipment [36, 37, 48]. Our data are consistent with observations on Ep1 endocytosis already reported more than two decades ago by Lehnert [49] and by Adamson and Bowden [50–52].

In fact, directly after inhalation and 24 h p.e. TiO_2_-NP, AuNP, and IrNP were not only found in Ep1 and Ep2 but also in the interstitial connective tissue and interstitial fibroblasts and across the basal membrane in endothelial vascular cells of rats and mice [53, 54].

**Fig. 10:**
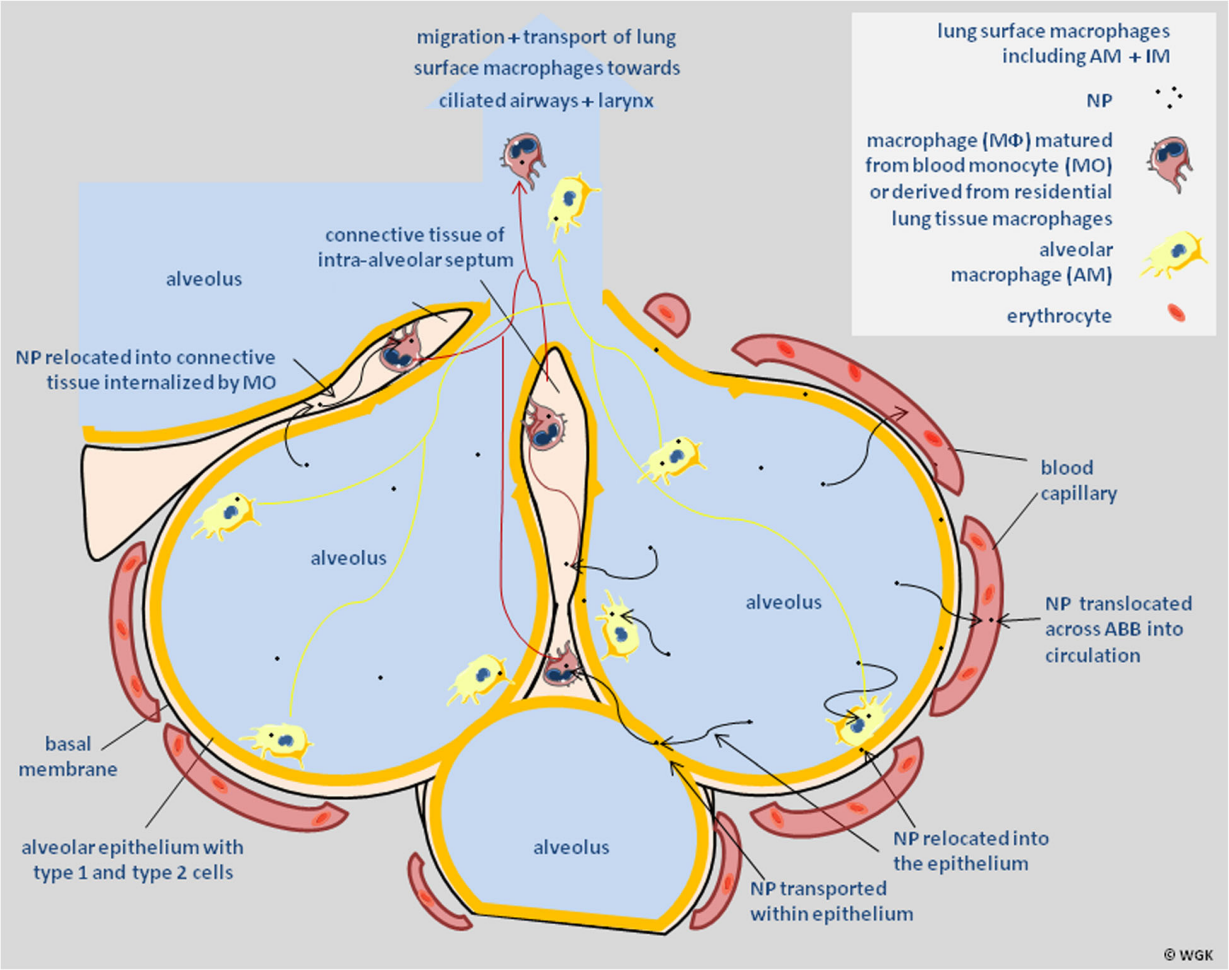
Mechanisms and pathways of NP relocation, NP re-entrainment, and translocation across the ABB. Graphical sketch of mechanisms and pathways of NP relocation into the epithelium and the interstitium, subsequent re-entrainment back onto the alveolar epithelium and translocation across the ABB into blood circulation for distribution throughout the entire organism

The graphical sketch in Fig. 10 illustrates the possible kinetics of NP relocation from the lung epithelium to interstitial spaces and re-entrainment back to the epithelium for LT-MC towards the larynx and GIT as recently reviewed by Stone and co-workers [55].

More recently, using in vitro cell culture studies, Thorley and co-workers [56] provided additional evidence for NP exocytosis out of Ep1 as was already reported by [49–52]. NP exocytosis allows for retention and transport within the interstitial connective tissue. Increasing [^48^V]TiO_2_-NP fractions may then cross the basal membrane and enter blood vessels for distribution in the body and subsequently retention in secondary organs and tissues as shown in Fig. 4b; indeed, cumulative translocated fractions rise from 0.01 directly after the 2 h inhalation to 0.15 after 28d p.e.

However, higher [^48^V]TiO_2_-NP fractions are eliminated via the tracheobronchial tree towards the larynx into the GIT for fecal excretion - called LT-MC. According to our BAL analyses in Fig. 1a after 24 h p.e. virtually all [^48^V]TiO_2_-NP are found in lung surface macrophages (including not only AM but eventually also airway macrophages and other phagocytes). As shown in Fig. 2c the daily cleared LT-MC fractions from the lung periphery starts at 0.026 d^− 1^ and declines gradually thereafter; hence, cumulative fractions in Fig. 2b rise to 0.40 within 28d p.e. which is almost threefold more than the cumulative translocated fraction (0.15) scross the ABB to blood in this period. Given the daily cleared fractions ranging within 0.03–0.02 d^− 1^, the BAL fractions over 28d p.e. would be expected to decrease rapidly between 24 h and 7d p.e. but they remain above 0.02 d^− 1^ over time (Fig. 1).

Therefore, the question arises as to how this [^48^V]TiO_2_-NP pool is replenished on the alveolar surface? Recent data on the origin and the replenishment of AM provide evidence for two sources (a) monocytes arriving from blood which mature to phagocytic macrophages in the interstitium while on their way to the alveolar surface and (b) phagocytic macrophages derived from lung-resident mononuclear phagocytes (including monocytes, dentritic cells and AM) of embryogeneic origin and distinct from hematopoietic monocytes [57–59]. In contrast to earlier notions the recent papers note that interstitial macrophages (IM) are rarely found in the alveolar-capillary lung interstitium but are instead found in bronchiolar tissues showing low phagocytic capacity when compared to AM; instead, IM respond to pathogenic and inflammatory stimuli. Nevertheless, both sources of AM replenishment are phagocytes arriving from interstitial sites towards the lung surface. Therefore, we hypothesize that these phagocytes take up interstitially retained [^48^V]TiO_2_-NP replenishing the pool of AM and thereby also refilling the pool of [^48^V]TiO_2_-NP on the alveolar epithelium for subsequent LT-MC to the larynx and GIT. In addition to AM replenishment, [^48^V]TiO_2_-NP retained in epithelial cells may gradually be released by apoptotic Ep1 [60] or exocytosed onto the alveolar surface for uptake by AM.

We cannot exclude NP passage through bronchus-associated lymphoid tissue (BALT) at bronchiolar−alveolar duct junctions back onto the bronchiolar epithelium. [51, 61, 62] However, BALT plays an important immunogenic role for fluid absorbed from the alveolar surface, but the reverse flow onto the epithelial surface was postulated in the literature but not proven. [63, 64] Furthermore, there are only about 30–50 BALT sites in the rat lungs, [49, 65] which are far too few than would be required for NP re-entrainment because LT-MC clearance is the most prominent clearance mechanism eliminating most of the longterm retained NP.

In our previous studies [66, 67] the AuNP or IrNP fractions in BAL-cells 24 h p.e. were about half of that found for [^48^V]TiO_2_-NP and remained at fractions of about 0.1 until 28d p.e. Hence, when estimating AuNP or IrNP fractions associated with the AM-pool (taking the incomplete AM lavagebility of our BAL methodology into account and normalizing to the contemporary lung burden (CLR)), these fractions remained constant at about 0.2. This is in contrast to the increasing [^48^V]TiO_2_-NP fractions associated with the AM-pool in Fig. 9 which continuously rise up to 0.6 at 28d p.e. This results from the higher rate of [^48^V]TiO_2_-NP arriving from interstitial and epithelial sites at the alveolar surface compared to elimination rate towards ciliated airways and larynx. In contrats, it seems that for AuNP or IrNP the rate of arrival on the alveolar surface is balanced by the LT-MC rate. It does not appear plausible that the kinetics of re-entrainment would be modified by the modest load – both in terms of NP mass and number – of any of the three NP types within the migrating cells. This NP-material dependent arrival rate on the alveolar surface requires further investigation.

### Inhaled or IT-instilled [^48^V]TiO_2_-NP fractions in secondary organs and tissues relative to those [^48^V]TiO_2_-NP which had crossed the ABB – a comparison to the fate of IV-injected [^48^V]TiO_2_-NP

The data sets obtained after inhalation in the current study or after intratracheal instillation [29] agree very well with each other for each organ or tissue, but they are strikingly different from those after IV-injection of [^48^V]TiO_2_-NP [28]; a detailed analysis is given in the Additional file 1 and the data are shown in Additional file 1: Figure S6 of the Supplementary Information.

Retained NP crossing the lung-ABB into circulation will interact with the mononucleated phagocyte systems (MPS) and are eventually phagocytized by cells of the MPS *such as* macrophages, neutrophils, and monocytes which exist in most organs (e.g. liver, spleen, kidneys), in skeletal bone marrow and in other tissues (e.g. connective tissue, muscle). Hence, NP will be cleared from the blood. However, as reviewed by Hume [68] the locations of MPS cells, their status of differentiation and their surface markers differ between organs. In fact, the rates of phagocytosis depend not only on NP properties like size, shape and various surface parameters but also on the different functions of MPS cells in different organs. As a result, the distribution, retention, and clearance of NP in these organs are complex and not yet fully understood [28, 29, 69, 70]. During the first hours post-injection, free IV-injected [^48^V]TiO_2_-NP will most likely have bound to high affinity blood proteins/biomolecules such as fibrinogen but to a lesser extent to albumin, etc. [71] depending on the surface chemistry and structure of the [^48^V]TiO_2_-NP. This may modulate the interactions between [^48^V]TiO_2_-NP and membrane receptors of the MPS cells of any given organ or tissue. The MPS cells of blood – dominated by monocytes – are floating and differ consistently from the resident MPS cells in secondary organs [68]. Therefore, only a small [^48^V]TiO_2_-NP fraction may have been taken up in blood MPS cells. Instead, [^48^V]TiO_2_-NP uptake from circulating blood is governed by the cells of the MPS in each of the various organs and tissues [29]. More surprising is that inhaled, as well as IT-instilled [^48^V]TiO_2_-NP, which only gradually translocated across the ABB into blood (resulting in low blood concentrations), show only low liver accumulation, and the highest accumulation in soft tissue in terms of the total amount of [^48^V]TiO_2_-NP per organ or tissue^2^ (see Additional file 1: Figure S6 in the Supplementary Information). This indicates that the scavenging MPS cells of the liver, that recognize IV-injected [^48^V]TiO_2_-NP with high efficiency, almost fail to recognize the [^48^V]TiO_2_-NP that reached the blood after crossing the ABB. In contrast, the MPS cells and maybe other cells of the soft tissue (which includes the vasculature of blood and lymphatic drainage) do recognize the lung-applied and translocated [^48^V]TiO_2_-NP. In other words, the lung-applied [^48^V]TiO_2_-NP which translocated across the ABB were either (a) surface-modified by biomolecules immediately in the lungs and/or (b) in the blood after passage through the ABB, and/or else (c) the much lower concentration of translocated [^48^V]TiO_2_-NP in blood after application in the lungs than after IV injection promotes uptake by MPS cells of soft tissue in contrast to those of liver MPS cells as mentioned above. It could be that all factors are at work, and additionally, it cannot be excluded that (d) the TiO_2_-NP were transported by blood cells e.g. phagocytes, monocytes, lymphocytes, thrombocytes after endocytosis, which would further complicate the situation. More literature from recent years provides better insights into TiO_2_-NP interactions with these cell types, like the lipid peroxidation of plasma membranes and its modulation by corona-forming serum proteins [72, 73]. Indeed, recent progress in the characterization and modes of NP-corona interactions affecting the translocation across organ membranes have been made, e.g. focusing on stable binding, high affinity proteins - so-called “core” proteins, etc., [8, 71, 74–76]. Yet, the complexity of interactions and the plethora of plasma proteins and biomolecules in the various body fluids potentially involved in corona formation as well as the diversity of NP-cell-receptor interactions hampers a better understanding, which however would be of great value for NP application in nanomedicine and drug delivery. The data presented in Additional file 1: Figure S6 in the Supplementary Information substantiate the conclusions drawn from our previous biokinetics studies after IT-instillation versus IV-injection of 70 nm sized TiO_2_-NP [28, 29], and for 20 nm sized AuNP after itratracheal inhalation [23], IT instillation [66] and IV-injection [77]: “*All these biokinetics data clearly demonstrate the invalidity and impracticability of IV-injection studies using suspended NP as surrogate approaches to study the biokinetics and toxic responses of inhaled or IT-instilled NP*”.

### [^48^V]TiO_2_-NP retention in the trachea and main bronchi

Three processes may contribute to the accumulation of [^48^V]TiO_2_-NP in the trachea, the first bifurcation, and main bronchi. Firstly, [^48^V]TiO_2_-NP may be retained in cells of the airway epithelium, secondly, they may be retained and accumulated in tracheobronchial and hilar lymph nodes, or thirdly they may be in transit as there is a continuous transport towards the larynx of [^48^V]TiO_2_-NP deposited on distal lung epithelia. The latter transport is initially caused by fast MCC of [^48^V]TiO_2_-NP deposited on the epithelium of conducting airways followed by LT-MC from the peripheral lungs at a rate of r_LT-MC_ ≈ 0.02–0.03 /d of the contemporary lung burden [25, 26, 35]. Making use of literature data for the tracheal mucus velocity v_TMV_ and the length of the trachea and the first bronchi which are reported for Wistar/Kyoto rat as v_TMV_ ≈ 1.9 mm/min [78], lengths of trachea and main bronchi l_tr_ ≈ 28 mm and l_br_ ≈ 4 mm, respectively, the fraction f_tr_ of [^48^V]TiO_2_-NP in transition during LT-MC can be estimated according to

**Table:** 

f_tr_ = r_LT − MC_ × t_res_/1440 min	with t_res_=(l_tr_ + l_br_)/v_TMV_	(1)

assuming a continuous LT-MC transport at a constant rate r_LT-MC_ of 0.02–0.03 d^− 1^ taken from Fig. 2c. The fractional amount f_tr_ corresponds to a [^48^V]TiO_2_-NP residence time t_res_ within the trachea and bronchi divided by 1440 min of one day. This yields t_res_ = 17 min and fractional [^48^V]TiO_2_-NP amounts f_tr_ in the range of 0.00024 to 0.00036 for the dissection time points 7 days and 28 days p.e. Thus, the estimated amounts of [^48^V]TiO_2_-NP in transition in the tracheal sample are about one tenth of the experimental data compiled in Table 3 for these dissection time points, which implies that [^48^V]TiO_2_-NP in transition can account for a maximum of 10% of the [^48^V]TiO_2_-NP determined in the tracheal samples.

For the dissection time points 1 h, 4 h and 24 h p.e. the fraction of [^48^V]TiO_2_-NP in the tracheal samples cannot be estimated with sufficient precision because fast MCC is strongly time dependent. The MCC data compiled in Table 2 show that immediately after the 2-h inhalation only about 4% of the initially deposited [^48^V]TiO_2_-NP had been swallowed and had arrived in the GIT. At 4 h p.e. this fraction increased rapidly to 20% and only a small further increase to about 25% of the initially deposited [^48^V]TiO_2_-NP occurs between 24 h and 28d p.e. Thus, fast MCC is essentially finished within 24 h p.e. Therefore, the [^48^V]TiO_2_-NP fractions in transit in the tracheal samples after 24 h and most likely already after 4 h are dominated by the LT-MC rate, r_LT-MC_ which yields similar values f_tr_ as those estimated above for the period between 7 days and 28 days p.e. Compared with the experimental data for the tracheal samples determined after 4 h and 24 h presented in Table 3 these transitional fractions of [^48^V]TiO_2_-NP in the tracheal sample are about 40 times lower, confirming that [^48^V]TiO_2_-NP in transit can only contribute a tiny part to the [^48^V]TiO_2_-NP observed. Hence, the observed amounts of [^48^V]TiO_2_-NP in the tracheal samples should be explained either by retention in hilar lymph nodes and/or by retention in the epithelium of the trachea and bronchi.

### The contribution of [^48^V]TiO_2_-NP absorbed in the GIT to the retention in secondary organs and tissues

Strictly speaking, the measured fractions in each secondary organ and tissue resulted not only from ABB-translocation into circulation but also from GIT absorption into the circulation of those [^48^V]TiO_2_-NP which were cleared initially by MCC and later by LT-MC into the GIT. Based on the results of GIT-absorption and accumulation in secondary organs and tissues after oral administration of 70 nm [^48^V]TiO_2_-NP reported in our previous biokinetics study after oral application (gavage) [30], we can roughly estimate this contribution. The previous, orally applied [^48^V]TiO_2_-NP were somewhat larger than the inhaled [^48^V]TiO_2_-NP of the current inhalation study which would probably lead to lower GIT-absorption; furthermore, only data at 24 h and 7 days of the previous gavage study are available; these GIT-absorbed fractions are presented in the first two columns of Table 4. Calculating the fractions of inhaled [^48^V]TiO_2_-NP cleared after 24 h by MCC into the GIT and the sum of all [^48^V]TiO_2_-NP which had entered the GIT within the first 7 days p.e. (Table 2), and multiplying these fractions with the absorbed organ fractions of the previous oral study (first two columns of Table 4) will provide the contribution of GIT-absorbed [^48^V]TiO_2_-NP shown in the 3rd and 4th column of Table 4. In the last two columns of Table 4, ratios of the GIT-absorbed fraction over the measured fraction of each organ and tissue are provided. These ratios are always smaller than 0.01 for each of the organs indicating a minute contribution of the GIT-absorbed [^48^V]TiO_2_-NP. Ratios are a bit larger for carcass and its two constituents, skeleton and soft tissue, but are still below 0.05. We estimated similar minor contributions for GIT-absorption after the inhalation of 20 nm [^195^Au] AuNP [23]. Hence, we conclude that ABB-translocation into circulation is the dominant post-inhalation absorption mechanism that leads to subsequent accumul ation in secondary organs and tissues.

**Table 4:** Fractional contribution of [^48^V]TiO_2_-NP absorbed in the GIT to the retention in secondary organs and tissues

	GIT-absorbed NP fraction after NP gavage [23]		Estimated NP fraction of GIT-absorption cleared from lungs		Ratio: GIT-absorb. NP over measured fractions after NP inhalation	
2nd organs	24 h	7d	24 h	7d	24 h	7d
liver	1.27E-04	2.26E-05	1.21E-07	4.62E-08	4.53E-03	5.67E-03
spleen	3.19E-05	1.16E-05	1.68E-09	2.37E-09	2.50E-04	5.65E-04
kidneys	3.82E-05	2.30E-05	2.74E-08	3.19E-08	3.42E-03	3.86E-03
heart	7.96E-05	4.89E-06	2.78E-09	2.24E-10	1.66E-04	1.27E-04
brain	1.01E-05	2.38E-05	7.66E-11	1.12E-09	3.59E-05	1.31E-04
uterus	2.44E-05	4.37E-05	1.43E-09	3.61E-09	2.77E-04	2.29E-04
carcass	8.07E-04	2.88E-04	5.82E-06	4.51E-06	3.42E-02	4.34E-02
skeleton	4.39E-04	8.96E-04	1.87E-06	7.09E-06	2.02E-02	2.20E-02
soft tissue	4.58E-04	5.00E-06	1.40E-06	3.86E-08	1.45E-02	2.15E-02

Mean values of GIT-absorbed fractions after [^48^V]TiO_2_-NP gavage in the left two columns at 24 h or 7 days were taken from [23]. Estimated contribution of GIT-absorption of [^48^V]TiO_2_-NP cleared from lungs were calculated by multiplying the mean values of [^48^V]TiO_2_-NP cleared after 24 h by MCC into the GIT and the sum of all [^48^V]TiO_2_-NP which had entered the GIT within the first 7 days p.e. (Table 2) and the fraction in the according left column of the Table 4. These are shown in the middle columns of Table 4. The ratios of GIT-absorbed [^48^V]TiO_2_-NP at 24 h or 7 days divided by the mean values of the measured organ or tissue fractions of Table 3 after [^48^V]TiO_2_-NP inhalation are presented in the right columns of Table 4. These ratios confirm the rather low contribution of GIT-absorbed [^48^V]TiO_2_-NP to the measured accumulation of each secondary organ or tissue in Table 3

## Conclusion

Intratracheal inhalation of freshly generated [^48^V]TiO_2_-NP aerosols for two hours allows low dose deposition in the lungs of adult healthy rats. The sensitivity obtained with radiolabeled NP is sufficiently high to study quantitative [^48^V]TiO_2_-NP biodistributions in the rat organism, as well as clearance out of each rat after five different retention time intervals up to 28 days p.e. Highly sensitive gamma-ray-spectrometry, allows a dynamic, analytical range over five orders of magnitude across the different specimens.

Regarding the biokinetics results, the following can be concluded:

- Fast mucociliary airway clearance (fractional range 0.16 to 0.31) varies substantially. However, when analyzing only the biokinetics of [^48^V]TiO_2_-NP deposited in the peripheral lungs (alveolar region) the biological variability is considerably lower allowing for a more predictive statistical analysis in the peripheral lungs.
- Long-term macrophage-mediated clearance (LT-MC) from the alveolar region (0.40 ± 0.04, integral fraction at 28 days p.e.) is almost three times as high as the cumulative translocation across the air-blood-barrier (0.15 ± 0.01 at 28 days p.e.). Hence, ABB-translocation of [^48^V]TiO_2_-NP and [^192^Ir] IrNP (0.14 ± 0.07) are similar but significantly higher than those of [^195^Au] AuNP (0.04 ± 0.02) after 28 days. Most [^48^V]TiO_2_-NP remain in the alveoli (0.44 ± 0.05 at 28 days p.e.) but only half of these remain on the alveolar epithelium.
- [^48^V]TiO_2_-NP are initially relocated from the epithelium into the interstitial spaces, leaving only a fraction of 0.2 in the AM pool on the epithelium, which is similar to earlier findings for 20 nm inhaled AuNP and IrNP. The latter two NP types gradually re-entrained back to the epithelium, slowly enough that the NP fractions in the AM pool remained balanced at 0.2 due to the counter-balancing LT-MC action. However, [^48^V]TiO_2_-NP re-entrainment back to the epithelium occurred faster while the LT-MC rate to the larynx and GIT remained constant at fractional rates of 0.02–0.03 d^− 1^ such that the [^48^V]TiO_2_-NP fraction in the AM pool on the epithelium built up to almost 0.6. Underlying mechanisms and pathways of cell-mediated NP relocation and re-entrainment versus translocation are discussed and a graphical sketch is provided.
- Using results of previous [^48^V]TiO_2_-NP intratracheal instillation studies, where also auxiliary biokinetics studies were performed after intratracheal instillation of soluble [^48^V] Ti (NO_3_) salt solutions we showed that ionic ^48^V release from the inhaled [^48^V]TiO_2_-NP was negligible in the present study. As a result, we hypothesize that the small but detectable urinary fractions throughout the study period are particulate and may have passed renal filtration from blood as disagglomerated primary particles and/or smaller sized agglomerates/aggregates of [^48^V]TiO_2_-NP. The lack of ionic ^48^V release indicates also that the [^48^V] activity found in the blood is dominated by circulating [^48^V]TiO_2_-NP, including those determined in the residual blood volume of secondary organs.

## Materials and methods

### Study design

Twenty healthy, adult, female Wistar Kyoto rats were randomly assigned to five groups of four rats and subjected to intratracheal inhalation of a ^48^V-radiolabelled TiO_2_-NP aerosol for 2 hours via an endotracheal tube [79]. The biodistribution was analysed 1 h, 4 h, 24 h, 7d and 28d after exposure. The first group of rats, which was exsanguinated and dissected immediately after the two-hour intratracheal inhalation exposure, was assigned to the retention time point 1 h since the nanoparticles brought into the rats’ lungs during 2 hours had an average time of only 1 hour for deposition, uptake, and distribution.

### Animals and maintenance

The Wistar-Kyoto rats (WKY/Kyo@Rj rats, Janvier, Le Genest Saint Isle, France) were housed in relative-humidity and temperature controlled ventilated cages (VentiRack Bioscreen TM, Biozone, Margate, UK) on a 12-h day/night cycle. Rodent diet and water were provided ad libitum. The rats were adapted for at least 2 weeks after purchase and then randomly attributed to the experimental groups of the biokinetics study. When starting the study the rats were 8–10 weeks old and exhibited a mean body weight of 277 ± 17 g. Some physiological parameters of the rats are given in Table 2.

All experiments were conducted under German federal guidelines for the use and care of laboratory animals in accordance with EU Directive 2010/63/EU for animal experiments. They were approved by the Regierung von Oberbayern (Government of District of Upper Bavaria, Approval No. 211–2531-94/04) and by the Institutional Animal Care and Use Committee of the Helmholtz Centre Munich.

### Synthesis and characterization of the [^48^V]TiO_2_-NP aerosol

Radiolabeled TiO_2_-NP aerosols were produced continuously during the experiments by spark ignition between two pure titanium cylindrical electrodes (diameter 3 mm, length 5 mm) which had been proton irradiated in the compact cyclotron at the Zyklotron AG (Karlsruhe, Germany). The protons impinging on one of the flat ends of each electrode had an energy of about 13.5–14 MeV in order to achieve the highest possible ^48^V-activity near the surface. This activity was distributed nearly homogeneously throughout a layer thickness that would theoretically be consumed by the spark ignition process during the experiments. At the time the inhalation experiments were performed, the specific ^48^V radioactivity was 17.6 MBq/mg in a surface layer of about 30 to 40 μm thickness, similar to what had been described earlier in detail [43]. The radioactive ^48^V decays back to ^48^Ti via electron capture or positron emission, with a half-life of 15.97 days, thereby emitting γ-rays with an energy of 0.99 MeV and 1.3 MeV, as well as γ-rays of 511 keV that result from electron-positron annihilation. The latter was used for the γ-spectrometrical analyses.

For each group of rats, the [^48^V]TiO_2_-NP aerosol was freshly generated in the spark ignition aerosol generator (GFG100, Palas, Karlsruhe, Germany) at 50 Hz spark frequency in an argon (Ar) gas stream of 3 L/min. The electrically charged aerosol of initially 2–5 nm primary particles was immediately quasi-neutralized by an inline radioactive ^85^Kr source and the highly concentrated and continuously agglomerating [^48^V]TiO_2_-NP passed through a 30 cm long tubular furnace that was kept at a temperature of 950 °C to form single, more compact and more homogeneous, spherical 20 nm-sized [^48^V]TiO_2_-NP. Downstream of the furnace the aerosol was cooled and diluted in a copper tube (inner diameter 8 mm) by mixing with humidified oxygen and nitrogen to obtain an oxygen concentration of 20–25%. After dilution to concentrations of 1–3 · 10^6^ NP/cm^3^ further agglomeration was negligible within the few seconds prior to inhalation by the rats. As shown previously by TEM and HRTEM in Fig. 6 of [43] the 20 nm sized [^48^V]TiO_2_-NP still have a chain agglomerated/aggregated structure even after 950 °C heat-treatment albeit more compact than the non-heat-treated NP. In that report [43] we provide further comprehensive information of the physico-chemical aerosol analysis. The flow rate was typically 10 L/min and the relative humidity of the aerosol was set to about 70% before entering the inhalation apparatus, as described earlier [43] and schematically displayed in Additional file 1: Figure S2 of the Supplementary Information. The aerosol particle concentration and size distribution were continuously sampled and controlled by a condensation particle counter (CPC 3022A, TSI, Aachen, Germany) and a scanning mobility particle spectrometer (SMPS; consisting of a model 3071 differential mobility analyzer and a model 3010 CPC, TSI, Aachen, Germany), respectively. Averages of the count median diameters (CMD), volume median diameters (VMD) and geometric standard deviations (GSD) as well as number concentrations and volume concentrations are given as mean ± SD in Table 1. Since the SMPS instrument exhibited a lower particle size detection limit of 10 nm, the averaged spectra were fitted to a lognormal size distribution using the least squares method and the fits were extrapolated to a size of 1 nm (for details see Additional file 1). These corrections led to slightly lower CMDs while the GSD changed only negligibly (see Table 1). The characteristic parameters of the freshly generated [^48^V]TiO_2_-NP aerosol were similar to those previously reported [43] which included ^48^V-radio-labeling and excluded the radio-label using non-irradiated, pure titanium electrodes; TEM and HRTEM images were analyzed from the latter. Similarly, the chemical composition was determined by XEDS and Electron Energy Loss Spectrometry (EELS). Also from the non-radio-labeled aerosol, XRD analysis showed the particles had the crystalline TiO_2_ anatase structure. After the online heat treatment of the aerosol, the specific surface area was determined to be 270.7 m^2^•g^− 1^ by BET measurements. The specific ^48^V activity of the aerosol particles was determined by γ-spectrometric analysis of absolute filters onto which [^48^V]TiO_2_-NP had been collected at an aerosol flow (0.3 L/min) throughout each 2-h exposure period. From the activity deposited on the filter, an activity concentration of the [^48^V]TiO_2_-NP aerosol of 12.4 kBq•μg^− 1^ was derived. This value agrees well with the 17.6 kBq•μg^− 1^ for the pure titanium electrodes considering that 40% of the [^48^V]TiO_2_-NP mass is oxygen. At this activity concentration the atomic ratio of ^48^V:Ti in the nanoparticles is about 1.6 × 10^− 7^. Hence, statistically, from about 40 TiO_2_-NP only one will contain a ^48^V-radiolabel. Therefore, the ^48^V-radiolabeling involves a minimal impurity which would be highly unlikely to affect the stability and the physico-chemical characteristics of the [^48^V]TiO_2_-NP.

### Intratracheal inhalation exposure

Four slightly anesthetized adult rats were ventilated individually via a flexible endotracheal tube and placed on their left lateral side in an air-tight plethysmograph box of our tailor-made inhalation apparatus and connected to the aerosol system, (see Additional file 1: Figure S2 of Supplementary Information). They were exposed to the freshly generated aerosol for 2 hours. Details of the aerosol generation and inhalation setup have been described earlier [43]. In the remainder of this report, this exposure method will be called “intratracheal inhalation” (see reference [79]).

### Treatment of the rats after inhalation

Post exposure (p.e.) anesthesia of each rat was antagonized as described in the Additional file 1 and previously in [23, 29]. Each rat was kept individually in a metabolic cage and excreta were collected separately and quantitatively. For ethical reasons, the rats of the 28-day group were maintained individually in a normal cage on cotton cloths starting immediately after [^48^V]TiO_2_-NP inhalation. Each cloth was replaced with a new one every 3–4 days (2 cloths per week), and the fecal droppings were quantitatively separated from the previous one. After separation, the cloth contained only ^48^V originating from urine which had soaked and dried. In Additional file 1: Table S2 the list of collected organs, tissues, body fluids, and excretion are given. Since the cages of the four rats of each group were located next to each other, the rats had continuous sensory perception to each other.

### [^48^V]TiO_2_-NP relocation into the epithelium and interstitial spaces

The broncho-alveolar lavage (BAL) procedure used was able to distinguish between [^48^V]TiO_2_-NP associated with BAL cells (BALC) and free [^48^V]TiO_2_-NP in the supernatant BAL fluid (BALF) as described in detail in the Additional file 1. In order to compare the [^48^V]TiO_2_-NP fractions in BALC and BALF with those retained in the lavaged lungs, the [^48^V]TiO_2_-NP fractions were normalized to the fraction of contemporarily retained [^48^V]TiO_2_-NP in the total lungs at each retention time point, providing lavageable [^48^V]TiO_2_-NP fractions versus the intra-epithelial and interstitial retained non-lavagable [^48^V]TiO_2_-NP fraction. This allows a direct comparison of the values at each time retention point.

### [^48^V]TiO_2_-NP in the trachea and main bronchi

In many earlier inhalation studies performed with different species, it was reported that particles can be retained and accumulated efficiently in the hilar lymph nodes at the first bifurcation and along the trachea. Therefore, at each retention time point, the present study comprises an analysis of the content of [^48^V]TiO_2_-NP in the trachea including the first bifurcation, both main bronchi, and the hilar lymph nodes, in analogy to the attempt reported earlier after exposure to 20 nm [^195^Au] AuNP [23]).

### Evaluation and statistical analysis of [^48^V]TiO_2_-NP biokinetics

At 1 h, 4 h, 24 h, 7d, and 28d p.e., rats were anesthetized (by 5% isoflurane inhalation) and euthanized by exsanguination via the abdominal aorta. Blood, all organs, tissues, and excretions were collected, and the radioactivity of the ^48^V was determined by γ-spectrometry without any further physico-chemical processing of the samples, as described in the Additional file 1 and in earlier works [23, 28–30]. Throughout this report nanoparticle quantities are calculated from the ^48^V activity determined with γ-scintillation detectors, properly calibrated in γ-ray energy and detection efficiency for ^48^V, and corrected for background and radioactive decay during the experiments (see Additional file 1). Samples yielding background-corrected counts in the 511 keV region-of-interest of the ^48^V γ-spectrum were defined to be below the detection limit (<DL; 0.2 Bq) when the number of counts was less than three standard deviations of the background counts collected without any sample in the γ-scintillation detector.

Up to 24 h p.e. mucociliary tracheobronchial clearance (MCC) was measured in the GIT and feces. The MCC data of the 7d- and 28d-group were derived from fecal excretion measurements during the first 3 days p.e. and GIT content at dissection. After 3 days p.e. fecal excretion was considered as long-term macrophage-mediated clearance (LT-MC).

About 70% of the blood volume has been recovered by exsanguination. Thus, organs and tissues contain residual blood whose radioactivity needs to be subtracted to obtain the true nanoparticle content. For this purpose, the residual blood contents of organs and tissues after exsanguination were calculated by making use of the findings of Oeff & König [80], and the true radioactivities of the organs and tissues were obtained by subtracting the blood-bound ^48^V-radioactivity values. The procedure is outlined (see Eq. (17) to (23)) in the Additional file 1.

In the first step of the data evaluation, the measured ^48^V-activity values were expressed as fractions of the initial lung dose (ILD) i.e. the [^48^V]TiO_2_-NP radioactivity deposited in the lungs. Fractions were normalized to the sum of all sampled ^48^V radioactivities of a given rat (see Additional file 1). In a second step, fractions were normalized to the initial peripheral lung dose (IPLD) calculated as the ILD minus the fast mucociliary clearance (MCC) of [^48^V]TiO_2_-NP from the conducting airways in each animal. The mathematical procedure is derived in Eq. (12 + 13) of the Additional file 1. The reason for this additional normalization was simply to reduce the data scatter caused by the rather variable [^48^V]TiO_2_-NP deposition in the conducting airways to focus on the biokinetics data for the peripheral lungs. The fractions for each organ or tissue were averaged over the group of rats and were presented with the standard error of the mean (SEM). All calculated significances are based on One-Way-ANOVA analyses with the post-hoc Bonferoni test. In the case of direct comparisons of two groups, the unpaired t-test was used. Significance was considered at *p* ≤ 0.05.

The [^48^V]TiO_2_-NP biokinetics after intratracheal inhalation determined in the present study was compared with three previous studies that applied an aqueous suspension of [^48^V]TiO_2_-NP with a median size of 70 nm by intratracheal instillation or gavage, or intravenous injection [28–30]. The biokinetics data of lung-applied [^48^V]TiO_2_-NP were further normalized to the [^48^V]TiO_2_-NP fraction which had translocated across the ABB (see Eq. (24 + 25) in the Additional file 1). It should be noted that the suspended [^48^V]TiO_2_-NP were surface-modified with sodium pyrophosphate in contrast to the freshly spark-ignition generated pristine [^48^V]TiO_2_-NP.

### Biokinetics of soluble ^48^V after intratracheal instillation

Since the ^48^V-radiolabel is not intrinsic to the titania NP matrix we have to consider that a certain fraction of the ^48^V may be released from the TiO_2_-NP and could be distributed in organs and tissues as free, ionic ^48^V, thus affecting the TiO_2_-NP biokinetics analysis. In order to estimate such a release and to quantify its effect, control experiments with ionic ^48^V were carried out. As reported previously [29], an auxiliary study (AUX) was performed at 24 h and 7 d after intratracheal instillation (IT) with the purpose of correcting the biodistributions of [^48^V]TiO_2_-NP for ^48^V-ion release. In order to mimic ^48^V released by [^48^V]TiO_2_-NP, 0.33 μg•μL^− 1^ ionic Ti (NO_3_)_4_ was added to carrier-free ionic ^48^V. The pH value was adjusted to 5. For the experiments, 60 μL of a solution containing 27 kBq ionic ^48^V and 20 μg of ionic Ti were administered per rat. Based on the biodistribution of ^48^V-ions and the urinary excretion kinetics after IT of ^48^V-ions and of [^48^V]TiO_2_-NP, the biodistribution of [^48^V]TiO_2_-NP was corrected for contributions of ^48^V-ions according to the mathematical procedure derived in the Additional file 1. Basically, ignoring disagglomeration, it is assumed for conservative reasons, that the fractional urinary excretion of activity is entirely due to ^48^V-ions for both, the auxiliary ^48^V-ions biokinetics study and the [^48^V]TiO_2_-NP biokinetics study. Then the ratios of cumulated urinary ^48^V-ions excretion over ^48^V-ions retention in the total body are set equal for both, the auxiliary ^48^V-ions and the [^48^V]TiO_2_-NP study. From this equation, the retained ^48^V-ions fraction in the body can be estimated for the [^48^V]TiO_2_-NP study; in a next step also the retained ^48^V-ions fractions of each organ_i_ in the [^48^V]TiO_2_-NP study are estimated. These ^48^V-ions fractions are subtracted from the measured ^48^V fraction of each organ_i_ to determine the particulate fraction of this organ in the [^48^V]TiO_2_-NP study (the detailed derivation is provided in Additional file 1, section 14). Even under the rigid conservative assumption of exclusive ionic urinary excretion, we show in the Results section that the corrections of the organs for ionic ^48^V-release in Table 3 are minimal.

## Additional file

Additional file 1: The supplementary material associated with this paper is available free of charge on the BMC website. The following content is described: 1. Aerosol size distribution measurements using the SMPS and spectral fitting. 2. Lung and body retention fits. 3. Intratracheal inhalation exposure to the freshly generated [^48^V]TiO_2_-NP aerosol. 4. Preparation of biokinetics samples for radiometric analysis. 5. Radiometric and statistical analysis. 6. Bronchoalveolar lavage (BAL). 7. Parameters of inhalation and deposition. 8. Total [^48^V]TiO_2_-NP deposition in each rat determined by the balanced ^48^V activities of the entire dissected rat including its total excretion. 9. Long-term macrophage-mediated [^48^V]TiO_2_-NP clearance. 10. ^48^V activity determination of skeleton and soft tissue. 11. Blood correction and total blood volume. 12. [^48^V]TiO_2_-NP accumulation and retention in secondary organs and tissues relative to translocated [^48^V]TiO_2_-NP across the ABB. 13. Inhaled or IT-instilled [^48^V]TiO_2_-NP fractions in secondary organs and tissues relative to those [^48^V]TiO_2_-NP which had crossed the ABB – a comparison to the fate of IV-injected [^48^V]TiO_2_-NP. 14. Biokinetics of soluble, ionic ^48^V after intratracheal (IT) instillation. 15. [^48^V]TiO_2_-NP accumulation and retention in secondary organs and tissues: Data evaluation and correction for the release of ionic ^48^V from [^48^V]TiO_2_-NP. 16. References. (DOCX 990 kb)

## Footnotes

1 MCC and LT-MC can only be derived from the activity measured in fecal excretions, which exhibits a delay due to the time required for the passage through the GIT. This time delay causes a certain uncertainty when defining the time after which MCC is essentially concluded. Fecal excretions measured after 3 days are completely assigned to LT-MC which also starts immediately after inhalation and should therefore be extrapolated down to time zero. However, due to the inevitable uncertainties in determining MCC it appears exaggerated to further correct MCC for a minor contribution of early LT-AM-C to the fecal excretion data.

2 Note, based on [^48^V]TiO_2_-NP per gram of organ or tissue the concentration fractions of soft tissue are certainly much lower as shown in Table 3.

## References

[1] Robichaud CO, Uyar AE, Darby MR, Zucker LG, Wiesner MR (2009). Estimates of upper bounds and trends in Nano-TiO2 production as a basis for exposure assessment. Environmental Science & Technology.

[2] Industry TN (2013). Titanium Dioxide. vol. Sect. 3.30. pp. 181–186.

[3] Christensen FM, Johnston HJ, Stone V, Aitken RJ, Hankin S, Peters S, Aschberger K (2011). Nano-TiO(2) - feasibility and challenges for human health risk assessment based on open literature. Nanotoxicology.

[4] Maurer MM, Donohoe GC, Maleki H, Yi J, McBride C, Nurkiewicz TR, Valentine SJ (2016). Comparative plasma proteomic studies of pulmonary TiO2 nanoparticle exposure in rats using liquid chromatography tandem mass spectrometry. J Proteome.

[5] Stableton PA, Minarchick VC, McCawley M, Knuckles TL, Nurkiewicz TR (2012). Xenobiotic particle exposure and microvascular endpoints: a call to arms. Microcirculation.

[6] Gaté L, Disdier C, Cosnier F, Gagnaire F, Devoy J, Saba W, Brun E, Chalansonnet M, Mabondzo A (2017). Biopersistence and translocation to extrapulmonary organs of titanium dioxide nanoparticles after subacute inhalation exposure to aerosol in adult and elderly rats. Toxicol Lett.

[7] Pujalté I, Dieme D, Haddad S, Serventi AM, Bouchard M (2017). Toxicokinetics of titanium dioxide (TiO2) nanoparticles after inhalation in rats. Toxicol Lett.

[8] Whitwell H, Mackay RM, Elgy C, Morgan C, Griffiths M, Clark H, Skipp P, Madsen J (2016). Nanoparticles in the lung and their protein corona: the few proteins that count. Nanotoxicology.

[9] Jonasson S, Gustafsson Å, Koch B, Bucht A (2013). Inhalation exposure of nano-scaled titanium dioxide (TiO2) particles alters the inflammatory responses in asthmatic mice. Inhal Toxicol.

[10] Meldrum K, Guo C, Marczylo EL, Gant TW, Smith R, Leonard MO. Mechanistic insight into the impact of nanomaterials on asthma and allergic airway disease. Part Fibre Toxicol. 2017;14(45). 10.1186/s12989-017-0228-y. 10.1186/s12989-017-0228-y PMC5697410 29157272

[11] Baan RA (2007). Carcinogenic hazards from inhaled carbon black, titanium dioxide, and talc not containing Asbestos or asbestiform fibers: recent evaluations by an IARC monographs working group. Inhal Toxicol.

[12] NIOSH: Occupational exposure to titanium dioxide. NIOSH CURRENT INTELLIGENCE BULLETIN 63 2011, NIOSH Publication No.160:1–140.

[13] ECHA, Community rolling action plan (CoRAP) update covering years 2014, 2015 and 2016. https://echa.europa.eu/documents/10162/13628/corap_list_2014-2016_en.pdf/43cf5fd8-b9e6-4f0d-841c-6ba39ae4d6d4

[14] Ahmad J, Akhter S, Rizwanullah M, Amin S, Rahman M, Ahmad MZ, Rizvi MA, Kamal MA, Ahmad FJ. Nanotechnology-based inhalation treatments for lung cancer: state of the art. Nanotechnol Sci Appl. 2015;8:55–66. 10.2147/nsa.s49052. 10.2147/NSA.S49052 PMC4657804 26640374

[15] Di Cristo L, Maguire C, Mc Quillan K, Aleardi M, Volkov Y, Movia D, Prina-Mello A (2018). Towards the identification of an in vitro tool for assessing the biological behavior of aerosol supplied nanomaterials. Int J Environ Res Public Health.

[16] Fei Yin Z, Wu L, Gui Yang H, Hua Su Y (2013). Recent progress in biomedical applications of titanium dioxide. Phys Chem Chem Phys.

[17] Kuzmov A, Minko T (2015). Nanotechnology approaches for inhalation treatment of lung diseases. J Control Release.

[18] Loira-Pastoriza C, Todoroff J, Vanbever R (2014). Delivery strategies for sustained drug release in the lungs. Adv Drug Deliv Rev.

[19] Ferin J, Oberdorster G, Penney DP (1992). Pulmonary retention of ultrafine and fine particles in rats. Am J Respir Cell Mol Biol.

[20] Baisch BL, Corson NM, Wade-Mercer P, Gelein R, Kennell AJ, Oberdorster G, Elder A (2014). Equivalent titanium dioxide nanoparticle deposition by intratracheal instillation and whole body inhalation: the effect of dose rate on acute respiratory tract inflammation. Part Fibre Toxicol.

[21] Shi H, Magaye R, Castranova V, Zhao J (2013). Titanium dioxide nanoparticles: a review of current toxicological data. Part Fibre Toxicol.

[22] Geiser M, Kreyling W (2010). Deposition and biokinetics of inhaled nanoparticles. Particle and Fibre Toxicology.

[23] Kreyling WG, Möller W, Holzwarth U, Hirn S, Wenk A, Schleh C, Schäffler M, Haberl N, Gibson N, Schittny JC (2018). Age-dependent rat lung deposition patterns of inhaled 20 nanometer gold nanoparticles and their quantitative biokinetics in adult rats. ACS Nano.

[24] Kreyling WG, Semmler M, Erbe F, Mayer P, Takenaka S, Schulz H, Oberdörster G, Ziesenis A (2002). Translocation of ultrafine insoluble iridium particles from lung epithelium to extrapulmonary organs is size dependent but very low. Journal of Toxicology and Environmental Health-Part A.

[25] Semmler M, Seitz J, Erbe F, Mayer P, Heyder J, Oberdorster G, Kreyling WG (2004). Long-term clearance kinetics of inhaled ultrafine insoluble iridium particles from the rat lung, including transient translocation into secondary organs. Inhal Toxicol.

[26] Semmler-Behnke M, Takenaka S, Fertsch S, Wenk A, Seitz J, Mayer P, Oberdorster G, Kreyling WG (2007). Efficient elimination of inhaled nanoparticles from the alveolar region: evidence for interstitial uptake and subsequent reentrainment onto airways epithelium. Environ Health Perspect.

[27] Kreyling WG, Semmler-Behnke M, Seitz J, Scymczak W, Wenk A, Mayer P, Takenaka S, Oberdorster G (2009). Size dependence of the translocation of inhaled iridium and carbon nanoparticle aggregates from the lung of rats to the blood and secondary target organs. Inhal Toxicol.

[28] Kreyling WG, Holzwarth U, Haberl N, Kozempel J, Hirn S, Wenk A, Schleh C, Schäffler M, Lipka J, Semmler-Behnke M, Gibson N (2017). Quantitative biokinetics of titanium dioxide nanoparticles after intravenous injection in rats: part 1. Nanotoxicology.

[29] Kreyling WG, Holzwarth U, Haberl N, Kozempel J, Wenk A, Hirn S, Schleh C, Schäffler M, Lipka J, Semmler-Behnke M, Gibson N (2017). Quantitative biokinetics of titanium dioxide nanoparticles after intratracheal instillation in rats: part 3. Nanotoxicology.

[30] Kreyling WG, Holzwarth U, Schleh C, Kozempel J, Wenk A, Haberl N, Hirn S, Schäffler M, Lipka J, Semmler-Behnke M, Gibson N (2017). Quantitative biokinetics of titanium dioxide nanoparticles after oral application in rats: part 2. Nanotoxicology.

[31] (A.R.A.) ARA (2009). Multiple-path particle dosimetry model (MPPD version 3.0).

[32] ICRP-66 (1994). Human respiratory tract model for radiological protection. A report of a Task Group of the International Commission on Radiological Protection. Annals of the ICRP.

[33] Oberdörster G, Ferin J, Gelein R, Soderholm SC, Finkelstein J (1992). Role of the alveolar macrophage in lung injury: studies with ultrafine particles. Environ Health Perspect.

[34] Kreyling W, Scheuch G, Heyder J, Gehr P (2000). Clearance of particles deposited in the lungs. Particle Lung Interactions.

[35] Kreyling WG (1990). Interspecies comparison of lung clearance of “insoluble” particles. Journal of Aerosol Medicine.

[36] Geiser M, Casaulta M, Kupferschmid B, Schulz H, Semmler-Behnke M, Kreyling W (2008). The role of macrophages in the clearance of inhaled ultrafine titanium dioxide particles. Am J Respir Cell Mol Biol.

[37] Geiser M, Rothen-Rutishauser B, Kapp N, Schurch S, Kreyling W, Schulz H, Semmler M, Im Hof V, Heyder J, Gehr P (2005). Ultrafine particles cross cellular membranes by nonphagocytic mechanisms in lungs and in cultured cells. Environ Health Perspect.

[38] Mühlfeld C, Geiser M, Kapp N, Gehr P, Rothen-Rutishauser B (2007). Re-evaluation of pulmonary titanium dioxide nanoparticle distribution using the "relative deposition index": evidence for clearance through microvasculature. Particle and Fibre Toxicology.

[39] Hainfeld JF, Slatkin DN, Smilowitz HM (2004). The use of gold nanoparticles to enhance radiotherapy in mice. Phys Med Biol.

[40] Choi HS, Liu W, Misra P, Tanaka E, Zimmer JP, Ipe BI, Bawendi MG, Frangioni JV (2007). Renal clearance of quantum dots. Nat Biotechnol.

[41] Alric C, Miladi I, Kryza D, Taleb J, Lux F, Bazzi R, Billotey C, Janier M, Perriat P, Roux S, Tillement O (2013). The biodistribution of gold nanoparticles designed for renal clearance. Nanoscale.

[42] Chen F, Goel S, Hernandez R, Graves SA, Shi S, Nickles RJ, Cai W (2016). Dynamic positron emission tomography imaging of renal clearable gold nanoparticles. Small.

[43] Kreyling WG, Biswas P, Messing ME, Gibson N, Geiser M, Wenk A, Sahu M, Deppert K, Cydzik I, Wigge C (2011). Generation and characterization of stable, highly concentrated titanium dioxide nanoparticle aerosols for rodent inhalation studies. J Nanopart Res.

[44] Möller W, Gibson N, Geiser M, Pokhrel S, Wenk A, Takenaka S, Schmid O, Bulgheroni A, Simonelli F, Kozempel J (2013). Gold nanoparticle aerosols for rodent inhalation and translocation studies. J Nanopart Res.

[45] Ellender M, Hodgson A, Wood KL, Moody JC (1992). Effect of bronchopulmonary lavage on lung retention and clearance of particulate material in hamsters. Environ Health Perspect.

[46] Lehnert BE, Valdez YE, Tietjen GL (1989). Alveolar macrophage-particle relationships during lung clearance. Am J Respir Cell Mol Biol.

[47] Oberdörster G, Ferin J, Lehnert BE (1994). Correlation between particle size, in vivo particle persistence, and lung injury. Environ Health Perspect.

[48] Kapp N, Kreyling W, Schulz H, Im Hof V, Gehr P, Semmler M, Geiser M (2004). Electron energy loss spectroscopy for analysis of inhaled ultrafine particles in rat lungs. Microsc Res Tech.

[49] Lehnert BE (1992). Pulmonary and thoracic macrophage subpopulations and clearance of particles from the lung. Environ Health Perspect.

[50] Adamson IY, Bowden DH (1978). Adaptive responses of the pulmonary macrophagic system to carbon. II. Morphologic studies. Laboratory Investigation.

[51] Adamson IY, Bowden DH (1981). Dose response of the pulmonary macrophagic system to various particulates and its relationship to transepithelial passage of free particles. Exp Lung Res.

[52] Bowden DH, Adamson IY (1984). Response of pulmonary macrophages to unilateral instillation of carbon. Am J Pathol.

[53] Geiser M, Quaile O, Wenk A, Wigge C, Eigeldinger-Berthou S, Hirn S, Schaffler M, Schleh C, Moller W, Mall MA, Kreyling WG (2013). Cellular uptake and localization of inhaled gold nanoparticles in lungs of mice with chronic obstructive pulmonary disease. Part Fibre Toxicol.

[54] Geiser M, Stoeger T, Casaulta M, Chen S, Semmler-Behnke M, Bolle I, Takenaka S, Kreyling WG, Schulz H (2014). Biokinetics of nanoparticles and susceptibility to particulate exposure in a murine model of cystic fibrosis. Part Fibre Toxicol.

[55] Stone Vicki, Miller Mark R., Clift Martin J.D., Elder Alison, Mills Nicholas L., Møller Peter, Schins Roel P.F., Vogel Ulla, Kreyling Wolfgang G., Alstrup Jensen Keld, Kuhlbusch Thomas A.J., Schwarze Per E., Hoet Peter, Pietroiusti Antonio, De Vizcaya-Ruiz Andrea, Baeza-Squiban Armelle, Teixeira João Paulo, Tran C. Lang, Cassee Flemming R. (2017). Nanomaterials Versus Ambient Ultrafine Particles: An Opportunity to Exchange Toxicology Knowledge. Environmental Health Perspectives.

[56] Thorley AJ, Ruenraroengsak P, Potter TE, Tetley TD (2014). Critical determinants of uptake and translocation of nanoparticles by the human pulmonary alveolar epithelium. ACS Nano.

[57] Reddy SP, Mehta D (2017). Lung interstitial macrophages redefined: it is not that simple anymore. Am J Respir Cell Mol Biol.

[58] Gibbings SL, Thomas SM, Atif SM, McCubbrey AL, Desch AN, Danhorn T, Leach SM, Bratton DL, Henson PM, Janssen WJ, Jakubzick CV (2017). Three unique interstitial macrophages in the murine lung at steady state. Am J Respir Cell Mol Biol.

[59] Liegeois M, Legrand C, Desmet CJ, Marichal T, Bureau F (2018). The interstitial macrophage: a long-neglected piece in the puzzle of lung immunity. Cell Immunol.

[60] Maynard RL, Donaldson K, Tetley TD (2013). Type 1 pulmonary epithelial cells: a new compartment involved in the slow phase of particle clearance from alveoli. Nanotoxicology.

[61] Macklin CC (1955). Pulmonary sumps, dust accumulations, alveolar fluid and lymph vessels. Acta Anat.

[62] Oberdorster G, Oberdorster E, Oberdorster J (2005). Nanotoxicology: an emerging discipline evolving from studies of ultrafine particles. Environ Health Perspect.

[63] Nunn J.F. (1993). Functional anatomy of the respiratory tract. Nunn's Applied Respiratory Physiology.

[64] Weibel ER (2015). On the tricks alveolar epithelial cells play to make a good lung. Am J Respir Crit Care Med.

[65] Plesch BE (1982). Histology and immunohistochemistry of bronchus associated lymphoid tissue (BALT) in the rat. Adv Exp Med Biol.

[66] Kreyling WG, Hirn S, Moller W, Schleh C, Wenk A, Celik G, Lipka J, Schaffler M, Haberl N, Johnston BD (2014). Air-blood barrier translocation of tracheally instilled gold nanoparticles inversely depends on particle size. ACS Nano.

[67] Kreyling WG, Semmler-Behnke M, Takenaka S, Moller W (2013). Differences in the biokinetics of inhaled nano- versus micrometer-sized particles. Acc Chem Res.

[68] Hume DA (2008). Differentiation and heterogeneity in the mononuclear phagocyte system. Mucosal Immunol.

[69] Almeida JPM, Chen AL, Foster A, Drezek R (2011). In vivo biodistribution of nanoparticles. Nanomedicine.

[70] Zarschler K, Rocks L, Licciardello N, Boselli L, Polo E, Garcia KP, De Cola L, Stephan H, Dawson KA (2016). Ultrasmall inorganic nanoparticles: state-of-the-art and perspectives for biomedical pplications. Nanomedicine.

[71] Neagu M, Piperigkou Z, Karamanou K, Engin AB, Docea AO, Constantin C, Negrei C, Nikitovic D, Tsatsakis A (2017). Protein bio-corona: critical issue in immune nanotoxicology. Arch Toxicol.

[72] Runa S, Lakadamyali M, Kemp ML, Payne CK (2017). TiO2 nanoparticle-induced oxidation of the plasma membrane: importance of the protein Corona. J Phys Chem B.

[73] Yusoff R, Kathawala MH, Nguyen LTH, Setyawati MI, Chiew P, Wu Y, Ch'ng AL, Wang ZM, Ng KW (2018). Biomolecular interaction and kinematics differences between P25 and E171 TiO2 nanoparticles. NanoImpact.

[74] Docter D, Westmeier D, Markiewicz M, Stolte S, Knauer SK, Stauber RH (2015). The nanoparticle biomolecule corona: lessons learned – challenge accepted?. Chem Soc Rev.

[75] Jo M-R, Yu J, Kim H-J, Song JH, Kim K-M, Oh J-M, Choi S-J (2016). Titanium dioxide nanoparticle-biomolecule interactions influence Oral absorption. Nanomaterials (Basel, Switzerland).

[76] Treuel L, Docter D, Maskos M, Stauber RH (2015). Protein corona – from molecular adsorption to physiological complexity. Beilstein Journal of Nanotechnology.

[77] Hirn S, Semmler-Behnke M, Schleh C, Wenk A, Lipka J, Schaffler M, Takenaka S, Moller W, Schmid G, Simon U, Kreyling WG (2011). Particle size-dependent and surface charge-dependent biodistribution of gold nanoparticles after intravenous administration. Eur J Pharm Biopharm.

[78] Felicetti SA, Wolff RK, Muggenburg BA (1981). Comparison of tracheal mucous transport in rats, Guinea pigs, rabbits, and dogs. J Appl Physiol Respir Environ Exerc Physiol.

[79] Osier M, Baggs RB, Oberdorster G (1997). Intratracheal instillation versus intratracheal inhalation: influence of cytokines on inflammatory response. Environ Health Perspect.

[80] Oeff K, Konig A (1955). Blood volume of rat organs and residual amount of blood after blood letting or irrigation; determination with radiophosphorus-labeled erythrocytes. Naunyn Schmiedebergs Arch Exp Pathol Pharmakol.

