# Supplementary material for: Quantitative biokinetics over a 28 day period of freshly generated, pristine, 20 nm titanium dioxide nanoparticle aerosols in healthy adult rats after a single two-hour inhalation exposure
Source: Part Fibre Toxicol. 2019 Jul 9;16:29. doi: 10.1186/s12989-019-0303-7 (PMC6617842; doi:10.1186/s12989-019-0303-7)
Supplement: Supplementary file 1 — The supplementary material associated with this paper is available free of charge on the BMC website. The following content is described: 1. Aerosol size distribution measurements using the SMPS and spectral fitting. 2. Lung and body retention fits. 3. Intratracheal inhalation exposure to the freshly generated [48V]TiO2-NP aerosol. 4. Preparation of biokinetics samples for radiometric analysis. 5. Radiometric and statistical analysis. 6. Bronchoalveolar lavage (BAL). 7. Parameters of inhalation and deposition. 8. Total [48V]TiO2-NP deposition in each rat determined by the balanced 48V activities of the entire dissected rat including its total excretion. 9. Long-term macrophage-mediated [48V]TiO2-NP clearance. 10. 48V activity determination of skeleton and soft tissue. 11. Blood correction and total blood volume. 12. [48V]TiO2-NP accumulation and retention in secondary organs and tissues relative to translocated [48V]TiO2-NP across the ABB. 13. Inhaled or IT-instilled [48V]TiO2-NP fractions in secondary organs and tissues relative to those [48V]TiO2-NP which had crossed the ABB – a comparison to the fate of IV-injected [48V]TiO2-NP. 14. Biokinetics of soluble, ionic 48V after intratracheal (IT) instillation. 15. [48V]TiO2-NP accumulation and retention in secondary organs and tissues: Data evaluation and correction for the release of ionic 48V from [48V]TiO2-NP. 16. References. (DOCX 990 kb) [file 12989_2019_303_MOESM1_ESM.docx]

## Supplementary Information

**Quantitative biokinetics during 28 days after a two-hour inhalation of freshly generated, pristine, 20 nm titanium dioxide nanoparticle aerosols by healthy adult rats**

Wolfgang G. Kreyling*^1,2^ Uwe Holzwarth^3^, Carsten Schleh^1,4^, Stephanie Hirn^1^, Alexander Wenk^1,5^, Martin Schäffler^1^, Nadine Haberl^1^, Manuela Semmler-Behnke^1^, Neil Gibson^3^

^1^ Comprehensive Pneumology Center, Institute of Lung Biology and Disease, Helmholtz Zentrum München – German Research Center for Environmental Health, Ingolstaedter Landstrasse 1, D-85764 Neuherberg / Munich, Germany

^2^ Institute of Epidemiology, Helmholtz Center Munich – German Research Center for Environmental Health, Ingolstaedter Landstrasse 1, D-85764 Neuherberg / Munich, Germany

^3^ European Commission, Joint Research Centre (JRC), Ispra, Italy

^4^ Current address: Abteilung Gesundheitsschutz, Berufsgenossenschaft Holz und Metall, Am Knie 8, D-81241 München, Germany

^5^ Current address: Dept. Infrastructure, Safety, Occupational Protection, German Research Center for Environmental Health, Ingolstaedter Landstrasse 1, D-85764 Neuherberg / Munich, Germany

***Corresponding author**Dr. Wolfgang G. Kreyling
Institute of Epidemiology
Helmholtz Centre Munich, German Research Center for Environmental Health
Ingolstaedter Landstrasse 1,

85764 Neuherberg / Munich, Germany
Email: [kreyling@helmholtz-muenchen.de](mailto:kreyling@helmholtz-muenchen.de),

Phone: +49-89-2351-4817

**Email addresses of the authors:**

[kreyling@helmholtz-muenchen.de](mailto:kreyling@helmholtz-muenchen.de)

[Uwe.HOLZWARTH@ec.europa.eu](mailto:Uwe.HOLZWARTH@ec.europa.eu)

[Carsten.Schleh@bghm.de](mailto:Carsten.Schleh@bghm.de)

[stephanie.hirn@med.uni-muenchen.de](mailto:stephanie.hirn@med.uni-muenchen.de)

[alexander.wenk@helmholtz-muenchen.de](mailto:alexander.wenk@helmholtz-muenchen.de)

[MSBiology@gmx.de](mailto:MSBiology@gmx.de)

[haberl.nadine10@googlemail.com](mailto:haberl.nadine10@googlemail.com)

[Manuela.Behnke@lgl.bayern.de](mailto:Manuela.Behnke@lgl.bayern.de)

[neilgibson15@gmail.com](mailto:neilgibson15@gmail.com)

The supporting information is available free of charge on the BMC website.

**Content**

1. Aerosol size distribution measurements using the SMPS and spectral fitting
2. Lung and body retention fits
3. Intratracheal inhalation exposure to the freshly generated [^48^V]TiO_2_-NP aerosol
4. Preparation of biokinetics samples for radiometric analysis
5. Radiometric and statistical analysis
6. Bronchoalveolar lavage (BAL)
7. Parameters of inhalation and deposition
8. Total [^48^V]TiO_2_-NP deposition in each rat determined by the balanced ^48^V activities of the entire dissected rat including its total excretion
9. Long-term macrophage-mediated [^48^V]TiO_2_-NP clearance
10. ^48^V activity determination of skeleton and soft tissue

## Blood correction and total blood volume

## [^48^V]TiO_2_-NP accumulation and retention in secondary organs and tissues relative to translocated [^48^V]TiO_2_-NP across the ABB

1. Inhaled or IT-instilled [^48^V]TiO_2_-NP fractions in secondary organs and tissues relative to those [^48^V]TiO_2_-NP which had crossed the ABB – a comparison to the fate of IV-injected [^48^V]TiO_2_-NP
2. Biokinetics of soluble, ionic ^48^V after intratracheal (IT) instillation
3. [^48^V]TiO_2_-NP accumulation and retention in secondary organs and tissues: Data evaluation and correction for the release of ionic ^48^V from [^48^V]TiO_2_-NP
4. References

## Aerosol size distribution measurements using the SMPS and spectral fitting

For each two-hour inhalation exposure, the [^48^V]TiO_2_-NP size distribution was measured continuously by SMPS resulting in about 40 spectra. The SMPS (consisting of a model 3071 differential mobility analyzer and a model 3010 CPC, TSI, Aachen, Germany) was operated at a main flow of 6 L/min and a sample flow of 0.6 L/min providing spectra in the range of 10 nm – 420 nm. Count median diameters (CMD) and geometric standard deviations (GSD) were averaged and given as mean ± SD in Table 1. In addition, the provided SMPS parameters, volume median diameters (VMD) as well as number concentrations and volume concentrations, were averaged as well. Since the measured spectra were cut below 10 nm, spectral data down to a size of 1 nm were obtained by extrapolation of the averaged spectrum of 40 measured number size distribution spectra that was fitted to a lognormal size distribution minimizing the sum of least squares for the independent variables: median diameter, geometric standard deviation and spectral peak height. Since the measured spectral data > 100 nm became noisy at the level below 10^-4^ of peak maximum, the fit was performed in the size range of 1 -100 nm. These corrections led to only minimally lower CMDs, GSD and VMDs (see Table 1).

## Lung and body retention fits

The lung retention $LR(t)$ in Figure S1A is based on the data obtained from the five dissection time points (Table 3: 2^nd^ line of “total lungs”) which were fitted to a mono exponential function using a least squares algorithm resulting in

|  | $LR(t)=0.943\times\exp\left\{-\frac{t\times\ln2}{2\text{5.4 d}}\right\}$. | (1a) |
| --- | --- | --- |

For comparison, the body retention, BR(t) of the 28d group of rats, in Figure S1B is dominated by the lung retention with minor contributions of the translocated fractions that are present in all secondary organs, tissues, and blood (see Table 3). It can be estimated from the excretion data determined between day 3 and day 28 according to:

|  |  | t ∊ [3, 28 d] | (1b) |
| --- | --- | --- | --- |

where $\bar{a}_{\text{feces,cumul}}(t)$and $\bar{a}_{\text{urine,cumul}}(t)$ denote the mean cumulative activity fractions of the 28d group of rats excreted up to time $t$ via feces and urine, respectively. A least square fit of the body retention to a mono-exponential function gives

|  | $BR(t)=0.894\times\exp\left\{-\frac{t\times\ln2}{3\text{2.9 d}}\right\}$. | t ∊ [3, 28 d] | (1c) |
| --- | --- | --- | --- |

**Figure S1**: **A:** Mean lung retained fractions (IPLD) obtained from five dissection time points (circles, data taken from Table 3); half-life is 25 d. **B:** Mean body retention of the 28 day group of rats from day 3 to day 28 according to Eqn (1b) (upright triangles) together with least squares fits using one exponential term or two terms; half-lives are 33 days and 38 d, respectively. For comparison data between day 4 and day 7 from the 7-day group of rats (crosses) are presented. (2-column fitting image)

While in Fig. S1A the fitted exponential term is essentially determined by three time points – day 1, day 7 and day 28 – the fitted half-life of 25 d can only be a rough estimate. In contrast, in Fig. S1B body retention provides more data points indicating that 12 days p.e. the data decrease more slowly than before. This is reflected in the slightly increased half-live of 38 days when fitting the data to a bi-exponential term; the fit to a single exponential term yields a half-live of only 33 days. The body retention data between day 3 and day 7 of the 28d group of rats are very well confirmed by those of the 7d group of rats.

## Intratracheal inhalation exposure to the freshly generated [^48^V]TiO_2_-NP aerosol

As described earlier [[1](#_ENREF_1), [2](#_ENREF_2)], four rats were anesthetized by an intramuscular injection of a mixture of Medetomidin (15 *μ*g/100 g body weight), Midazolam (0.2 mg/100 g body weight), and Fentanyl (0.5 *μ*g/100 g body weight). For endotracheal intubation of the healthy, female, adult rats a flexible cannula (16 G, 1.7 mm diameter, 50 mm length) was placed in the upper trachea under visual control and sealed against outside air with a modified pipette tip wedged gently into the laryngeal opening [[1-3](#_ENREF_1)]. The animals were placed on their left lateral side in one of the four plethysmographs (50 mm diameter, 20 cm length, volume 390 cm³) of the inhalation apparatus and connected with the endotracheal cannula to the aerosol line outside of the plethysmograph. Ventilation was computer controlled with a negative pressure of −1.5 kPa applied to the plethysmograph for 0.5 s of inspiration followed by 0.5 s spontaneous expiration at ambient air pressure, resulting in a breathing frequency of 60 min^−1^. This ventilation pattern caused inspiration of about 75% of total rat lung capacity (TLC), so animals were slightly hyperventilated and did not breathe spontaneously but followed the computer-controlled breathing pattern, Figure S2.

After the intratracheal inhalation, anesthesia of each rat was antagonized by subcutaneous injection of a mixture of Atipamezol (0.075 mg/100 g body weight), Flumazenil (20*μ*g/100 g body weight), and Naloxon (10 *μ*g/100 g body weight) as described earlier [[1](#_ENREF_1), [2](#_ENREF_2), [4](#_ENREF_4)].

- 1500 Pa

O

2

N

2

filter

sample

Ar

Ti

**Ti**

spark

generator

CPC

DMPS

-30 Pa

HUM.

Control

anesthetized

intubated rats

ventilated in

plethysmographs

Kr-85

600°C, tube

furnace

^48^V labeled

HEPA filtered secondary containment for radiation protection

Cu-tube

**Figure S2**: Schematics of the spark ignition aerosol generator and the aerosol inhalation apparatus taken from Supplementary Information of [[5](#_ENREF_5)]. DMPS: Differential Mobility Particle Sizer; CPC: Condensation Particle Counter; HUM: Humidifier. (2-column fitting image)

The deposition fractions obtained from the intratracheally inhaled (*i.e.* low-pressure ventilated and endotracheally intubated) rats – given in Table 2 - were compared with the estimates by the MPPD Software (vs. 3.04) [[6](#_ENREF_6)] shown in Figure S3. In Table S1 the input parameters used for the MPPD modeling are compiled. The aerosol parameters are adjusted to the exposure conditions of the present study given in Table 1.

**Figure S3**: Total and regional deposition fractions in adult rats estimated by the MPPD Software (vs. 3.04) according to endotracheal breathing. All MPPD model parameters are listed in Table S1; TB – deposited fraction in the tracheo-bronchial region, Pulm - deposited fraction in the pulmonary region, *i.e.* IPLD. (2-column fitting image)

**Table S1:** Model parameters used in the MPPD simulation.

|  | |  | **MPPD model (version 3.04)** | |  |  |
| --- | --- | --- | --- | --- | --- | --- |
| **Species & Model** | | | **Physiology parameters** | | **aerosol parameters** | |
| species | adult Long Evans rat | | TLC (cm³) | 13.7 | CMD (µm) | 0.023 |
| geometry | assym. mult. path | | FRC (cm³) | 4.0 | GSD | 1.5 |
| exposure | endotracheal | | breath frequency (min^-1^) | 60 | density (g cm^-3^) | 1.8 |
| position | left lateral | | TV (cm³) | 4.0 | aspect ratio | 1 |
|  |  | | inspir. fract. | 0.5 | aerosol concen-tration (mg m^-3^) | 0.15 |
|  |  | | pause | 0 |  |  |

## Preparation of biokinetics samples for radiometric analysis

At the chosen retention times of "immediately after inhalation" (1h), 4h, 24h, 7d or 28d after a 2-hour [^48^V]TiO_2_-NP intratracheal inhalation, rats were anesthetized (by 5 % isoflurane inhalation) and euthanized by exsanguination via the abdominal aorta as described earlier [[1](#_ENREF_1), [2](#_ENREF_2), [7-9](#_ENREF_7)]. Note the immediate time point after the 2-hour inhalation is set to be the 1h time point. Approximately 70 % of the total blood volume could be recovered. Total organs, tissues, the entire remaining carcass, and total fecal and urinary excretions were collected for radiometric analysis (see Table S2). During dissection, none of the organs were cut and all fluids were cannulated (where necessary) in order to avoid any cross contamination. In addition, broncho-alveolar lavages (BAL) were performed. Organs, tissues, carcass, BAL and excretions were collected for radio-analysis (see Table S2).

**Table S2:** Total organs, tissue and other samples prepared for radiometric analysis:

| lungs* | trachea + main bronchi, incl. hilar lymph nodes | | BAL cells* | BAL fluid* |
| --- | --- | --- | --- | --- |
| liver_(2nd)_ | spleen_(2nd)_ | | kidneys_(2nd)_ |  |
| brain_(2nd)_ | heart_(2nd)_ | | uterus_(2nd)_ |  |
| GIT: gastro-intestinal tract, comprising esophagus, stomach, small and large intestine | | | | |
| total skin_(2nd)_ | muscle sample_(2nd)_ | skinned head_(2nd)_  without brain | | |
| exsanguinated blood sample_(2nd)_ | | | | |
| bone sample_(2nd)_  (humerus or femur carefully cleaned from soft tissue) | | | | |
| carcass_(2nd)_: total remaining carcass beyond the listed organs and tissues including the skeleton and soft tissue but excluding the skin | | | | |
| Soft tissue: all tissues of the remaining carcass without skeleton | | | | |
| Secondary organs_(2nd)_: the sum of all secondary organs indexed with _(2nd)_. | | | | |
| excretion: total daily urine and faeces, collected separately; in the 28-day group integral urinary *versus* fecal excretion was collected over 3-4 days as described above | | | | |

* Lavaged lungs; broncho-alveolar lavage (BAL); separation of BAL cells from BAL fluid supernatant by centrifugation as described in [[10](#_ENREF_10)]

(2nd) The index “_(2nd)_“ indicates those secondary organs and tissues in which [^48^V]TiO_2_-NP may have accumulated after translocation across the air-blood-barrier (ABB) into blood circulation.

## Radiometric and statistical analysis

The ^48^V radioactivity of all samples was measured by γ-ray spectrometry without any further physico-chemical sample preparation in either a lead-shielded 10-mL or a lead-shielded 1-L well type NaI(Tl) scintillation detector as previously described {[[2](#_ENREF_2), [7](#_ENREF_7), [8](#_ENREF_8), [10](#_ENREF_10)]. For radiometric analysis the 511 keV γ-ray emission – resulting from electron-positron annihilation – was used; see further details in [[11](#_ENREF_11)]. The count rates were corrected for physical decay and background radiation. Additionally, count rates were calibrated to a ^48^V reference source ([^48^V]TiO_2_-NP filter sample of 2155 Bq (at reference date) whose activity was determined by an absolutely calibrated Ge/Li semiconductor detector in order to correlate ^48^V-radioactivity to the mass of the TiO_2_-NP making use of the specific ^48^V-radioactivity per Ti mass of 17.6 MBq/mg determined for the activated Ti-electrode tips; this pure Ti-tip activity corresponded to a specific ^48^V-radioactivity of 12.4 MBq/mg of the spark-ignition generated [^48^V]TiO_2_-NP taking the molecular O_2_ content into account. Samples yielding net counts (*i.e.*, background-corrected counts) in the 511 keV region-of-interest of the ^48^V γ-ray spectrum were considered below the detection limit when they were less than three standard deviations of the background count rate in this region-of-interest. The background count rate was determined without any sample in the detector well. The detection limit (DL) corresponded to 0.2 Bq.

For a complete balance of the deposited ^48^V radioactivity within the lungs of each rat, the ^48^V activities of all individual samples (including those taken at the dissection time point and those during excreta collection) were summed up for each rat and the sum was used as a denominator for the calculation of the ^48^V activity fraction in each sample per Initial Lung Dose (ILD). However, in a second step all sample ^48^V activities were additionally normalized to the Initial Peripheral Lung Dose (IPLD); *IPLD is obtained by* subtracting the ^48^V-activity deposited in the conducting airways rapidly cleared within 24h from the airways by mucociliary action (MCC)) *from* ILD - see eqns. 10, 11 below. As detailed there, MCC was determined from the measured ^48^V-activity in samples of the gastro-intestinal-tract and fecal excretaduring the first days p.e. The reason for this additional normalization step is to reduce the data scatter caused by the rather variable [^48^V]TiO_2_-NP deposition in the conducting airways which weakens the statistical power of the biokinetics data for the peripheral lungs, this being the focus of this report. Note, MCC data do not contribute to the slow, long-term TiO_2_-NP clearance processes from the peripheral lungs, like translocation across the ABB, transport towards the larynx, etc. The fractions for each rat were averaged over the four rats of each group and are reported with the standard error of the mean (SEM) as described earlier [[1](#_ENREF_1), [2](#_ENREF_2), [7](#_ENREF_7), [8](#_ENREF_8), [10](#_ENREF_10)].

All calculated significances are based on a One-Way-ANOVA test and a *post hoc* Bonferoni Test. In case of an individual two group comparison, the unpaired t-test was used. Significance was considered at p ≤ 0.05.

## Bronchoalveolar lavage (BAL)

Serial broncho-alveolar lavages (BAL) of rat lungs were performed at each time point of dissection, to assess [^48^V]TiO_2_-NP retention on the lung epithelium as free [^48^V]TiO_2_-NP suspended in the BAL-fluid, as [^48^V]TiO_2_-NP associated with freely moving lung surface macrophages or as [^48^V]TiO_2_-NP relocated from the epithelial surface either bound and/or taken up by cells of the epithelial barrier and beyond. BALs were performed by applying 6 x 5 ml of phosphate-buffered-saline solution (PBS without Ca^2+^ or Mg^2+^) under gentle massage of the thorax. The recovered BAL fluid (BALF) (about 80% of instilled PBS) was centrifuged at 500 g for 20 min at room temperature to separate the lavaged cells from the supernatant: The [^48^V]TiO_2_-NP content was determined by γ-ray-spectrometry. At each BAL the total number of lavaged cells was acquired using a hemocytometer by a dilution of the spun-down cells trypan-blue stained. For cell differentiation, cyto-centrifuged slide preparations of the lavaged cells were Wright-Giemsa stained for each sacrificed animal (see Figure S4). Immediately after intratracheal inhalation, we observed a transient neutrophilic influx of 7% in BAL which declined in the following to below 2%. During the first day p.e. lymphocytes reached percentages up to 5% and declined later to below 2%.

**Figure S4**: BAL cell differentiation at each time point of sacrifice. Mean ± SEM; n=4. (1-column fitting image)

By γ-ray-spectrometry, the fractions of free [^48^V]TiO_2_-NP in the BALF and of [^48^V]TiO_2_-NP in BAL cells and in the lavaged lungs were determined. Applying this BAL procedure we obtained about 4 • 10^6^ macrophages per BAL which is on average very similar to our previous studies [[1](#_ENREF_1), [2](#_ENREF_2), [12](#_ENREF_12)]. Normalizing the number of totally recovered macrophages of each BAL to the mean number of the total surface macrophage population of (12.5 ± 0.8) • 10^6^, previously determined in the lungs of WKY rats [[12](#_ENREF_12)], we estimated the total fraction of [^48^V]TiO_2_-NP associated with lung surface macrophages (alveolar macrophages (AM) pool).

## Parameters of inhalation and deposition

In the following we use the index i to distinguish individual rats that are subjected to inhalation exposure of [^48^V]TiO_2_-NP for a duration of two hours in a group of animals labelled by the index j that were sacrificed after a certain retention period ("immediately after inhalation" (1h), 4h, 24h, 7d, and 28d).

During each inhalation exposure of a group j of rats, a [^48^V]TiO_2_-NP aerosol sample was collected on a filter, and the [^48^V]-activity $A_{\text{filt}}^{\text{j}}$ (Bq) on the filter was determined by γ-spectrometry. The aerosol volume $V_{\text{filt}}^{\text{j}}$ (L) was measured with a gas flow meter and the specific aerosol activity $a_{\text{filt}}^{\text{j}}$ (Bq/L) was calculated as

|  | $a_{\text{filt}}^{\text{j}}=A_{\text{filt}}^{\text{j}}/V_{\text{filt}}^{\text{j}}$ |  | (2) |
| --- | --- | --- | --- |

The mean total lung capacity (TLC) in mL of healthy, female WKY-Kyoto Wistar rats was determined by [[13](#_ENREF_13)] to be$TLC=0.047\cdot BW$ (*BW*= body weight in gram). At a ventilation-pressure of -1.5 kPa the tidal volume $TV_{\text{i}}^{\text{j}}$ of each intubated rat i of group j was set at 75% *TLC*, which gives

|  | $TV_{\text{i}}^{\text{j}}=0.75\cdot TLC_{\text{i}}^{\text{j}}=0.75\cdot0.047\cdot BW_{\text{i}}^{\text{j}}$ | $i=1,2,3,4 rats; j=1..5 group$ | (3) |
| --- | --- | --- | --- |

The rats were ventilated for a ventilation time $t_{\text{inhal}}$ of two hours at a ventilation rate $f_{\text{i}}^{\text{j}}$ of 60 min^-1^ set by the computer-controlled inhalation apparatus. The inhaled aerosol volume $V_{inhal,i}^{j}$ of each rat i of group j was calculated as

|  | $V_{\text{inhal,i}}^{\text{j}}=f_{\text{i}}^{\text{j}}\cdot TV_{\text{i}}^{\text{j}}\cdot t_{\text{inhal}}$ | $i=1,2,3,4 rats; j=1..5 group$ | (4) |
| --- | --- | --- | --- |

1. **Total [^48^V]TiO_2_-NP** **deposition in each rat determined by the balanced ^48^V activities of the entire dissected rat including its total excretion**

The inhaled aerosol activity$A_{\text{inhal,i}}^{\text{j}}$ is calculated from the product of the total inhaled volume $V_{\text{inhal,i}}^{\text{j}}$ determined from Eqn (4) and the specific ^48^V aerosol activity $a_{\text{filt}}^{\text{j}}$ ((Bq/L) (Eqn (2)) as

|  | $A_{\text{inhal,i}}^{\text{j}}=V_{\text{inhal,i}}^{\text{j}}\cdot a_{\text{filt}}^{\text{j}}$ | $i=1,2,3,4 rats; j=1..5 group$ | (5) |
| --- | --- | --- | --- |

In order to determine the initially deposited [^48^V]TiO_2_-NP activity $A\text{(ILD)}_{\text{i}}^{\text{j}}$ (Bq) in the lungs of each rat (*i.e.* ILD), which is smaller than the inhaled dose because a significant amount of nanoparticles are exhaled again, all γ-spectrometrically measured sample activities $A_{\text{sample k,i}}^{\text{j}}$were summed up, where the index k identifies all specimens (all samples of organs, tissues, total excretion of rat (i,j)) collected from a single animal i of group j according to

|  | $A(\text{ILD})_{i}^{j}=\sum_{\text{k}=1}^{\text{n}} A_{\text{sample k,i}}^{\text{j}}$ | k = 1..n; all samples of organs, tissues, total excretion of rat (i,j) $i=1,2,3,4 rats; j=1..5 group$ | (6) |
| --- | --- | --- | --- |

For the total deposited TiO_2_-NP mass $m\text{(ILD)}_{\text{i}}^{\text{j}}$ (µg) and TiO_2_-NP number $N\text{(ILD)}_{\text{i}}^{\text{j}}$ the total deposited [^48^V]TiO_2_-NP activity $A\text{(ILD)}_{\text{i}}^{\text{j}}$ of each rat was divided by the specific ^48^V activity concentration per [^48^V]TiO_2_mass (17.6 kBq/µg) or divided by the quotient of the specific aerosol activity $a_{\text{filt}}^{\text{j}}$(Eqn. 2) and the averaged aerosol number concentration $N\text{(Aer)}_{\text{i}}^{\text{j}}$ (Table 1) determined by the CPC 3022A, respectively.

|  | $m(\text{ILD)}_{\text{i}}^{\text{j}}=A(\text{ILD)}_{\text{i}}^{\text{j}}/(17.6\text{ MBq/mg})$ $N(\text{ILD})_{\text{i}}^{\text{j}}=N(\text{Aer})_{\text{i}}^{\text{j}}\frac{A(\text{ILD)}_{\text{i}}^{\text{j}}}{a_{\text{filt, i}}^{\text{j}}}$ | $i=1,2,3,4 rats; j=1..5 group$ | (7a)  (7b) |
| --- | --- | --- | --- |

In addition, the deposited [^48^V]TiO_2_-NP activity fraction was determined relative to the total inhaled aerosol activity of each rat according to Eqn (5).

|  | $a\text{(ILD)}_{\text{i}}^{\text{j}}=\frac{A\text{(ILD)}_{\text{i}}^{\text{j}}}{V_{\text{inhal,i}}^{\text{j}}\cdot a_{\text{filt}}^{\text{j}}}$ | $i=1,2,3,4 rats; j=1..5 group$ | (8) |
| --- | --- | --- | --- |

In the next step [^48^V]TiO_2_-NP activity fractions in organs and tissues (index k) $a\text{(ILD)}_{\text{sample k,i}}^{\text{j}}$with respect to the deposited dose in the lungs were calculated by normalizing all measured sample activities $A_{\text{sample k,i}}^{\text{j}}$ (Bq) to the total deposited ^48^V activity $A\text{(ILD)}_{\text{sample k,i}}^{\text{j}}$ (Bq) of each rat (i,j) according to

|  | $a(\text{ILD)}_{\text{sample k,i}}^{\text{j}}=\frac{A_{\text{sample k,i}}^{\text{j}}}{A\text{(ILD)}_{\text{i}}^{\text{j}}}$ | $i=1,2,3,4 rats; j=1..5 group$ k = 1..n; all samples of organs, tissues, total excretion of rat (i,j) | (9) |
| --- | --- | --- | --- |

As mentioned above, mucociliary clearance (MCC) of [^48^V]TiO_2_-NP from thoracic airways after intratracheal inhalation was considered to contribute negligibly to the translocation across the air-blood-barrier (ABB). Therefore, for calculations of [^48^V]TiO_2_-NP translocation across the ABB, MCC was excluded from the complete balance by subtracting the ^48^V-radioactivity contributions of the head (without brain), trachea, gastro-intestinal tract (GIT), and feces obtained during the first 2-3 days p.e. from the overall radioactivity balance of each animal and normalizing the activities determined in all other organs and tissues to the new (reduced) balance. Thus, the amount of intratracheally inhaled material at the time $t=0$ that is available for translocation through the ABB is reduced due to MCC within the first 48h after instillation by

|  | $A\text{(ILD)}_{\text{MCC,i}}^{\text{j}}=A\text{(ILD)}_{\text{head}-\text{brain,i}}^{\text{j}}+A\text{(ILD)}_{\text{trachea,i}}^{\text{j}}+A\text{(ILD)}_{\text{GIT,i}}^{\text{j}}+A\text{(ILD)}_{\text{feces,i}}^{\text{j}}$ | | (10) | |
| --- | --- | --- | --- | --- |
|  | $a\text{(ILD)}_{\text{MCC,i}}^{\text{j}}=\frac{A\text{(ILD)}_{\text{MCC,i}}^{\text{j}}}{A\text{(ILD)}_{\text{i}}^{\text{j}}}$ | $i=1,2,3,4 rats; j=1..5 group$ | | (11) |

Where $A\text{(ILD)}_{\text{head}-\text{brain,i}}^{\text{j}}$ $(t;t\leq24\text{h})$ denotes the ^48^V-activity determined for the head without the brain, $A\text{(ILD)}_{\text{trachea,i}}^{\text{j}}$ $(t;t\leq24\text{h})$the ^48^V-activity in the trachea, $A\text{(ILD)}_{\text{GIT,i}}^{\text{j}}$ $(t;t\leq48\text{h})$the one in the gastro-intestinal tract and $A\text{(ILD)}_{\text{feces,i}}^{\text{j}}$ $(t;t\leq48\text{h})$ the activity of the feces collected during the first 48h p.e.

For the estimation of the [^48^V]TiO_2_-NP activity deposited on the alveolar epithelium $A\text{(IPLD)}_{\text{i}}^{\text{j}}$, the fast cleared MCC activity $A\text{(ILD)}_{\text{MCC,i}}^{\text{j}}$ is subtracted from the total deposited [^48^V]TiO_2_-NP activity $A\text{(ILD)}_{\text{i}}^{\text{j}}$determined according to Eqn (6). Thus, the activity of the initial peripheral lung dose $A\text{(ILPD)}_{\text{i}}^{\text{j}}$and the corresponding activity fraction $a\text{(IPLD)}_{\text{i}}^{\text{j}}$ normalized to the total initially deposited activity $A\text{(ILD)}_{\text{i}}^{\text{j}}$are

|  | $A\text{(IPLD)}_{\text{i}}^{\text{j}}=A\text{(ILD)}_{\text{i}}^{\text{j}}-A\text{(ILD)}_{\text{MCC,i}}^{\text{j}}$ $a\text{(IPLD)}_{\text{i}}^{\text{j}}=\frac{A\text{(ILD)}_{\text{i}}^{\text{j}}-A\text{(ILD)}_{\text{MCC,i}}^{\text{j}}}{A\text{(ILD)}_{\text{i}}^{\text{j}}}$ | $i=1,2,3,4 rats; j=1..5 group$ | (12) |
| --- | --- | --- | --- |

For any calculations of translocated fractions across the ABB, all sample fractions $\tilde{a}\text{(IPLD)}_{\text{sample k,i}}^{\text{j}}$are normalized to $A\text{(IPLD)}_{\text{i}}^{\text{j}}$ according to

|  | $\tilde{a}\text{(IPLD)}_{\text{sample k,i}}^{\text{j}}=\frac{A_{\text{sample k,i}}^{\text{j}}}{A\text{(IPLD)}_{\text{i}}^{\text{j}}}$ | $i=1,2,3,4 rats; j=1..5 group$ k = 1..n; all samples of organs, tissues, total excretion of rat (i,j) | (13) |
| --- | --- | --- | --- |

where the symbol $\tilde{a}$is used to emphasis that the normalization is done to a reduced balance $A\text{(IPLD)}_{\text{i}}^{\text{j}}$as the activity which is cleared by fast mucociliary clearance $A\text{(ILD)}_{\text{MCC,i}}^{\text{j}}$ is no longer available for translocation through the ABB.

1. **Long-term macrophage-mediated [^48^V]TiO_2_-NP clearance**

To estimate the long-term cleared [^48^V]TiO_2_-NP fraction cleared by macrophage-mediated transport LT-MC from the alveolar epithelium to the mucociliary escalator of the conducting airways, the total fecal excretion after day 2 is calculated by summing up all fecal samples from day 3 up to day m (d_m_).

|  | $\tilde{a}_{\text{LT}-\text{MC,i}}^{\text{j}}=\sum_{\text{l}=3}^{m} a_{\text{feces,l,i}}^{\text{j}}$ | l = 3,…m; d_m_ ∈ [3,…28 day] $i=1,2,3,4 rats; j=1..5 group$ | (14) |
| --- | --- | --- | --- |

## ^48^V-activity determination of skeleton and soft tissue

The ^48^V-activity in the whole skeleton of each rat $A_{\text{skeleton, i}}^{\text{j}}$was extrapolated from the activity of a bone sample $A_{\text{bone sample, i}}^{\text{j}}$ and its mass $m_{\text{bone sample, i}}^{\text{j}}$assuming the estimated weight of the skeleton to be 10% [[14](#_ENREF_14)] of the total body weight $m_{\text{tot body, i}}^{\text{j}}$

|  | $A_{\text{skeleton, i}}^{j}=A_{\text{bone sample, i}}^{j}\cdot\frac{m_{\text{tot body, i}}}{m_{\text{bone sample, i}}}\cdot0.1$ | $i=1,2,3,4 rats; j=1..5 group$ | (15) |
| --- | --- | --- | --- |

For this purpose, the bone sample was carefully cleaned from other tissue.

The ^48^V-activity to be assigned to the soft tissue $A_{\text{soft-tissue, i}}^{j}$ of each rat was calculated from the difference of the ^48^V-radioactivity content of the total remaining carcass $A_{\text{carcass, i}}$ (including soft tissue, muscle sample, skeleton, bone sample) and the activity in the skeleton as determined in Eqn (15) as

|  | $A_{\text{soft-tissue, i}}^{\text{j}}=A_{\text{carcass, i}}^{\text{j}}-A_{\text{skeleton, i}}^{\text{j}}$ | $i=1,2,3,4 rats; j=1..5 group$ | (16) |
| --- | --- | --- | --- |

## Blood correction and total blood volume

In order to obtain the true value of ^48^V-activity in the organs and tissues of interest, the radioactivity contributed by the residual blood retained after exsanguination had to be subtracted.

**Table S3:** Organ specific weight factors $f_{\text{organ,} \text{i}}^{\text{Oeff}}$ for the residual blood in the organ tissue after exsanguinations, given as residual blood weight per organ weight according to [[15](#_ENREF_15)].

| Lung (g•g^-1^) | liver (g•g^-1^) | spleen (g•g^-1^) | kidney (g•g^-1^) | brain (g•g^-1^) |
| --- | --- | --- | --- | --- |
| 0.28 ± 0.10) | 0.14 ± 0.03 | 0.16 ± 0.04 | 0.22 ± 0.04 | 0.018 ± 0.001 |
| heart (g•g^-1^) | GIT^§^  (g•g^-1^) | muscle (g•g^-1^) | fat (g•g^-1^) | thyroid* g/organ |
| 0.15 ± 0.02 | 0.020 ± 0.006 | 0.016 ± 0.002 | 0.012 ± 0.002 | 0.008 ± 0.001 |

* thyroid given for complete organ; ^§^ gastro-intestinal tract

The blood contents of organs and tissues were calculated according to the findings of Oeff and Konig [[15](#_ENREF_15)] shown in Table S3 and the ^48^V-radioactivities of the organs were corrected for these values as

|  | $A_{\text{organ}_{\text{k}}\text{, i}}^{\text{j}}=A_{\text{organ}_{\text{k}}\text{, i}}^{\text{measured, j}}-A_{\text{organ}_{\text{k}}\text{, i}}^{\text{blood} \text{correction, j}}$ | $i=1,2,3,4 rats; j=1..5 group$ | (17) |
| --- | --- | --- | --- |

where $A_{\text{organ}_{\text{k}}\text{,i}}^{\text{measured}}$denotes the ^48^V-activity measured in the organ "k" of rat _i,j_ ($i=1,2,3,4 rats; j=1...5\mathrm{group}$ for retention times of 1h, 4h, 24h, 7d, and 28d) in (Bq) which is corrected for the residual blood content by subtracting $A_{\text{organ}_{\text{k}}\text{, i}}^{\text{blood} \text{correction, j}}$calculated according to

|  | $A_{\text{organ}_{\text{k}}\text{,i}}^{\text{blood} \text{correction, j}}=\frac{A_{\text{blood} \text{sample, i}}^{\text{measured, j}}\cdot m_{\text{organ}_{\text{k}}\text{,i}}^{\text{measured, j}}\cdot f_{\text{organ}_{\text{k}}}^{\text{Oeff}}}{m_{\text{blood} \text{sample, i}}^{\text{measured, j}}}$ | $i=1,2,3,4 rats;$ $j=1..5 group$ | (18) |
| --- | --- | --- | --- |

making use of the mass $m_{\text{blood} \text{sample, i}}^{\text{measured, j}}$and the activity $A_{\text{blood} \text{sample, i}}^{\text{measured, j}}$measured for the blood recovered from exsanguination, the mass of the organ $m_{\text{organ}_{\text{k}},i}^{\text{measured, j}}$ and the organ specific weight factor $f_{\text{organ}_{\text{k}}}^{\text{Oeff}}$ for the residual blood in the organ tissue according to Oeff and Koenig (1955)[[15](#_ENREF_15)]. The total blood volume *BV* in (mL) was estimated to be

|  | $BV=0.06\cdot BW+0.77$ |  | (19) |
| --- | --- | --- | --- |

according to the work of Lee and Blaufox [[16](#_ENREF_16)], where *BW* denotes the body weight in (g).

To determine the ^48^V-activity in the residual blood of the remaining carcass or skeleton for each rat the following procedure was applied. Firstly, the mass of the residual blood volume in the carcass or skeleton $m_{\text{tiss}_{\text{k}}\text{, i}}^{\text{res.-blood, j}}$(tiss_k_ ∊ [carcass, skeleton]) was calculated by subtracting from the mass of the total blood volume $m_{BV}$ the mass of the sampled blood volume $m_{\text{meas.-blood sample, i}}^{\text{ j}}$ and the sum of the masses of the residual blood volumes of all organs $m_{\text{organ}_{\text{k}}\text{, i}}^{\text{res.-blood, j}}$ according to [[15](#_ENREF_15)]

|  | $m_{\text{tiss}_{\text{k}}\text{, i}}^{\text{res.-blood, j}}=m_{BV}-m_{\text{meas.-blood sample, i}}^{\text{j}}-\sum_{\text{l}\neq\text{k}} m_{\text{organ}_{\text{l}}\text{,i}}\cdot f_{\text{organ}_{\text{l}}\text{,i}}^{\text{Oeff}}$ | $i=1,2,3,4 rats;$ $j=1..5 group$ | (20) |
| --- | --- | --- | --- |

For each rat, the ^48^V-activity in the residual blood of the remaining carcass or skeleton $A_{\text{ts}_{\text{k}}}^{\text{blood correction}}(i,j)$( ts_k_ ∊ [carcass, skeleton]) is then given by

|  | $A_{\text{ts}_{\text{k}}\text{, i}}^{\text{res.-blood, j }}=\frac{A_{\text{meas.-blood sample, i}}^{\text{j}}}{m_{\text{meas.-blood sample, i}}^{\text{j}}}\cdot m_{\text{ts}_{\text{k}}\text{, i}}^{\text{res.-blood, j}}\cdot\frac{m_{\text{ts}_{\text{k}}\text{, i}}}{m_{\text{BW, i}}^{j}}$ | $i=1,2,3,4 rats;$ $j=1..5 group$ | (21) |
| --- | --- | --- | --- |

as the ^48^V-activity concentration determined from the blood sample taken times the mass of the residual blood in carcass or skeleton times the mass fraction of carcass or skeleton with respect to the rat’s body weight. This estimate assumes that the residual blood volume is proportional to the mass of either the carcass or skeleton. Since the remaining carcass consists of the skeleton and soft tissue, the ^48^V-activity in the residual blood of the soft tissue is the difference between ^48^V-activities of residual blood of carcass minus that of skeleton given by

|  | $A_{\text{soft-tissue, i}}^{\text{res.-blood, j}}=A_{\text{carcass, i}}^{\text{res.-blood, j}}-A_{\text{skeleton, i}}^{\text{res.-blood, j}}$ | $i=1,2,3,4 rats; j=1..5 group$ | (22) |
| --- | --- | --- | --- |

To determine the contribution of the ^48^V-activity in the residual blood to the total ^48^V-activity retained in all organs and tissues, the ratio $RB_{\text{organ(k), i}}^{\text{blood correction, j}}$

|  | $RB_{\text{organ}_{\text{k}}\text{, i}}^{\text{blood correction, j}}=\frac{A_{\text{organ}_{\text{k}}\text{, i}}^{\text{blood correction, j}}}{A_{\text{meas.-organ}_{\text{k}}\text{, i}}^{\text{j}}}$ | $i=1,2,3,4 rats; j=1..5 group$ | (23) |
| --- | --- | --- | --- |

is defined, where the ^48^V-activity of the residual blood retained in each organ,$A_{\text{organ(k), i}}^{\text{blood correction, j}}$, is calculated according to Eqn (18). As the retention of ^48^V-activity in blood, organs and tissues depends on time and follows different patterns the ratio $RB_{\text{organ(k), i}}^{\text{blood correction, j}}$ itself depends on time. This is shown in Fig. S5 for all organs, remaining carcass, skeleton, and soft tissue.

The ratios in Fig. S5 of are larger than 0.1 during the first 24-hours p.e. for most of the secondary organs and somewhat smaller in the carcass comprising soft tissue and skeleton. This may have resulted from both, the early ionic release of the ^48^V radiolabel and/or from initially rather high amounts of translocated and still circulating [^48^V]TiO_2_-NP in blood and retarded accumulations in the secondary organs, soft tissue and skeleton. But corrections are very small in the (lavaged) lungs since the dominant part of the [^48^V]TiO_2_-NP was retained in the lungs and the ^48^V activity concentration in blood was very low.

**Figure S5**: Ratio $RB_{\text{organ, i}}^{\text{blood correction}}$ of the ^48^V-activity in the residual blood over the measured organ or tissue activity. **A:** lavaged lungs, liver, spleen; **B:** kidneys, heart, brain, uterus; **C:** carcass, skeleton, soft tissue. Mean ± SEM, n ≥ 4 rats per time point. (1-column fitting image)

## [^48^V]TiO_2_-NP accumulation and retention in secondary organs and tissues relative to translocated [^48^V]TiO_2_-NP across the ABB

In order to determine the amount of [^48^V]TiO_2_-NP in secondary organs and tissues as a fraction of [^48^V]TiO_2_-NP that crossed the ABB, we have to normalize all activity values measured for all secondary organs and tissues $A_{\text{2nd organ, i }}^{\text{ j}}(t)$, all urine samples $A_{\text{urine, i }}^{\text{ j}}(t)$and all blood samples $A_{\text{blood} \text{sample, i}}^{\text{ j}}$to a value which is smaller than the value $A(t=0)=A_{0}$ that has been deposited after the 2-hour intratracheal inhalation. The normalization activity $A_{\text{t-loc,i}}^{\text{j}}$ is the sum of all$A_{\text{2nd organ, i }}^{\text{ j}}(t)$, $A_{\text{u}rine,\text{ i }}^{\text{ j}}(t)$,$A_{\text{blood} \text{sample, i }}^{\text{ j}}(t)$ . Therefore,

|  | $A_{\text{t-loc, i}}^{\text{j}}=\sum_{\text{k}=1}^{\text{n}} A_{\text{2nd organ, i}}^{\text{j,k}}+\sum_{\text{l}=1}^{\text{o}} A_{\text{blood sample, i}}^{\text{j,l}}+\sum_{\text{m}=1}^{\text{p}} A_{\text{urine, i}}^{\text{j, m}}$ | $i=1,2,3,4 rats;$ $j=1..5 group$ k=1,2..n; 2^nd^ organs  l=1,2..o; blood sample  m=1,2..p; urine sample | (24) |
| --- | --- | --- | --- |

holds for all rats i in each group j, which will be referred to as the translocated dose, which is a specific value for each rat used in the five retention time groups. The fractions of [^48^V]TiO_2_-NP that has been accumulated in organs and tissues k, blood l, and urine m after passing the ABB can now be determined for each rat by normalizing the activities $A_{\text{organ}_{\text{k}}\text{, i}}^{j}$ according to

|  | $\hat{a}_{\text{organ}_{\text{k}}\text{,i}}^{j}=\frac{A_{\text{organ}_{\text{k}}\text{,i}}^{j}}{A_{\text{t-loc,i}}^{\text{j}}}$ $\hat{a}_{\text{blood}_{\text{l}}\text{,i}}^{j}=\frac{A_{\text{blood}_{\text{l}}\text{,i}}^{j}}{A_{\text{t-loc,i}}^{\text{j}}}$ $\hat{a}_{\text{urine}_{\text{m}}\text{,i}}^{j}=\frac{A_{\text{urine}_{\text{m}}\text{,i}}^{j}}{A_{\text{t-loc,i}}^{\text{j}}}$ | $i=1,2,3,4 rats; j=1..5 group$ k=1,2..n; 2^nd^ organs  l=1,2..o; blood sample  m=1,2..p; urine sample | (25) |
| --- | --- | --- | --- |

Note, all urinary samples and the slow macrophage-mediated [^48^V]TiO_2_-NP fractions from days > 2 during the remaining retention time are included in the sum for normalization since both are considered to be part of the clearance and translocation.

1. **Inhaled or IT-instilled [^48^V]TiO_2_-NP fractions in secondary organs and tissues relative to those [^48^V]TiO_2_-NP which had crossed the ABB – a comparison to the fate of IV-injected [^48^V]TiO_2_-NP**

The data sets obtained after inhalation or intratracheal instillation [[17](#_ENREF_17)] in Fig. S6 are strikingly different from those after IV-injection of [^48^V]TiO_2_-NP [[18](#_ENREF_18)]. The lung-administered [^48^V]TiO_2_-NP fractions in Fig. S6 are normalized to the [^48^V]TiO_2_-NP fraction that translocated across the ABB, in order to directly compare organ and tissue accumulation with [^48^V]TiO_2_-NP in the circulation after IV-injection. However, note that translocation into blood is a process that extends over some time while a bolus of suspended [^48^V]TiO_2_-NP is instantaneously applied to the circulation during IV-injection. In our studies, IV-injection yields the highest initial [^48^V]TiO_2_-NP concentration in blood but this declines 500-fold during the first hour (Fig. S6C) since the MPS of the liver demonstrates its high capacity of effectively scavenging most of the IV-injected [^48^V]TiO_2_-NP and keeping them retained (Fig. S6A). Thereafter IV-injected [^48^V]TiO_2_-NP in blood are 100-fold and 30-fold lower than the translocated fractions of circulating [^48^V]TiO_2_-NP after IT-instillation or after [^48^V]TiO_2_-NP inhalation, respectively (Fig. S6C). Surprisingly, almost all translocated [^48^V]TiO_2_-NP after both modes of lung-administration are observed in the carcass, *i.e.* predominantly in soft tissue (Fig. S6D), and the translocated fractions retained in the liver are more than tenfold lower than after IV-injection (Fig. S6A). The biokinetics patterns in liver, spleen, and kidneys are very similar for both lung-administered [^48^V]TiO_2_-NP (Fig. S6A+C) although in both applications the TiO_2_-NP are chain-aggregated consisting of <10 nm primary particles but different in size: 20 nm inhaled TiO_2_-NP *versus* 50 nm instilled TiO_2_-NP [[17](#_ENREF_17)]. Throughout the entire retention time of 28d, the IV-injected [^48^V]TiO_2_-NP in the spleen are tenfold higher than those translocated fractions of the lung-administered [^48^V]TiO_2_-NP (Fig. S6A). Lung-administered translocated [^48^V]TiO_2_-NP fractions in kidneys are 50-fold higher during the first 24-hours p.e. than those of IV-injected [^48^V]TiO_2_-NP (Fig. S6C) and still tenfold higher after 28-days p.e.

During the first hours past injection, free IV-injected [^48^V]TiO_2_-NP will most likely have bound to highly abundant blood proteins/biomolecules such as albumin, *etc*. depending on the surface chemistry and structure of the [^48^V]TiO_2_-NP. This may modulate the interactions between [^48^V]TiO_2_-NP and membrane receptors of the MPS cells of a given organ or tissue. The MPS cells of blood - dominated by monocytes – are floating and differ consistently from the resident MPS cells of secondary organs. Therefore, only a small [^48^V]TiO_2_-NP fraction may have been taken up in blood MPS cells. Yet, [^48^V]TiO_2_-NP uptake from circulating blood is governed by the cells of the MPS in each of the various organs and tissues [[17](#_ENREF_17)]. More surprising is that inhaled as well as IT-instilled [^48^V]TiO_2_-NP, which only gradually translocated across the ABB into blood resulting in low blood concentrations, cause only low liver accumulation but highest accumulation in soft tissue in terms of the total amount of [^48^V]TiO_2_-NP per organ or tissue[[1]](#footnote-1). This indicates that the scavenging MPS cells of the liver that recognize IV-injected [^48^V]TiO_2_-NP with high efficiency do almost not recognize the [^48^V]TiO_2_-NP that reached the blood after crossing the ABB. In contrast, the MPS cells and maybe other cells of the soft tissue (which includes the vasculature of blood and lymphatic drainage) do recognize the lung-applied and translocated [^48^V]TiO_2_-NP. In other words, the lung-applied [^48^V]TiO_2_-NP which translocated across the ABB were either (a) surface-modified by biomolecules immediately in the lungs and/or (b) in the blood after passage through the ABB. Alternatively, it may be conceivable that (c) the AuNP were transported by blood cells *e.g.* phagocytes, monocytes, lymphocytes, thrombocytes after endocytosis or even by erythrocytes after adhesion to their surface membrane. While there is sufficient literature providing a phenomenological description of NP interactions with these cell types until now no quantitative assessment is available. The complexity of the plethora of biomolecules potentially involved in corona formation as well as the manifold of NP-cell-receptor interactions hampers a better understanding which is urgently needed for any NP application in nanomedicine and drug delivery. In fact, this lack of knowledge may be one of the underlying reasons for the limited successes of nanomedicinal applications of nanoparticles so far. Our biokinetics data also clearly demonstrate the invalidity and impracticability of IV-injection studies using suspended NP as surrogate approaches to study the biokinetics and toxic responses of inhaled or IT-instilled NP. The same conclusion was drawn from our recent biokinetics studies after IT-instillation *versus* IV-injection of 70 nm sized TiO_2_-NP[[17](#_ENREF_17), [18](#_ENREF_18)]. After 24h until 28d p.e. translocated fractions of inhaled [^48^V]TiO_2_-NP in liver, spleen, and kidneys increase about tenfold (Table 3, respective lines 6) while the translocated fractions in the carcass (*i.e.* predominantly in soft tissue), and also in heart and brain decline by an order of magnitude. These changes indicate ongoing redistribution from the latter organs/tissues to the former organs - besides urinary excretion. Only in the skeleton and the uterus fractions remain constant over time suggesting little redistribution.

**Figure S6:** [^48^V]TiO_2_-NP accumulation in secondary organs and tissues from 1h to 28d after intratracheal inhalation relative to the total of translocated [^48^V]TiO_2_-NP across ABB. **A:** liver and spleen; **B:** heart, brain, uterus; **C:** kidneys, blood; **D:** carcass, soft tissue, skeleton. Data are corrected for [^48^V]TiO_2_-NP retained in the residual blood volume of organs and tissues. Mean ± SEM, n=4 rats per time point. Two-way ANOVA statistical analyses between each secondary organ or tissue or blood after intratracheal inhalation or IT instillation *versus* IV-injection revealed highly statistical differences, p < 0.001; in contrast, no statistical differences were found after intratracheal inhalation *versus* IT instillation for each secondary organ or tissue or blood. (2-column fitting image)

1. **Biokinetics of soluble, ionic ^48^V after intratracheal (IT) instillation**

Ideally, the determined activity of ^48^V is directly proportional to the mass of the [^48^V]TiO_2_-NP. However, ^48^V ions that might become detached from the [^48^V]Ti O_2_-NP could compromise the accuracy of the determined NP biodistribution. Therefore, additional experiments had been performed earlier to investigate the translocation and biodistribution of soluble, ionic ^48^V at 24 hours and 7 days after IT-instillation [[18](#_ENREF_18)]. These data were used for correction of ionic ^48^V-release from the [^48^V]Ti O_2_-NP when exposed to body fluids. In order to mimic ^48^V release from the TiO_2_-NP we added to the carrier-free ionic ^48^V, 0.33 µg•µL^-1^ ionic Ti(NO_3_)_4_ in 60 µL of distilled water to obtain a nitrate solution of sufficient ionic strength stably maintaining the ^48^V-ions and adjusted the pH value to 5. Hence, 27 kBq of ionic ^48^V and 20 µg of ionic Ti were instilled during inspiration into the trachea of each rat followed by 300 – 400 µL of air. No losses of ionic ^48^V were found in the syringe and flexible cannula used for IT-instillation. The biokinetics was measured after 24h and 7d and the corresponding biodistributions are presented in Figure S7.

**Figure S7:** Biokinetics of soluble ionic ^48^V-radioisotope at 24 hours and 7 days after IT-instillation of a volume 60 µL of carrier free ^48^V in 0.33 µg•µL^-1^ ionic Ti(NO_3_)_4_ aqueous solution. Mean ± SEM, n = 4. Levels of significances: ** p<0.01; *** p<0.001. Taken from Supporting information of [[18](#_ENREF_18)]. (1.5-column fitting image)

The biodistribution after both time points was generally very similar to that found after IV injection with the highest amounts in urinary excretion followed by retention in the remaining carcass (including skeleton and soft tissues). However, in contrast to IV-injection [[17](#_ENREF_17)], retention in the lungs (7.8% and 3.3% after 24h and 7d, respectively) is higher than in the liver (2.8% and 2.9% after 24h and 7d, respectively), while blood levels are very similar after both routes of application including the decline from day 1 to day 7.

^48^V found in the GIT and feces may have been originated from both, mucociliary action towards the larynx and subsequent swallowing (fast lung clearance, MCC) and hepatobiliary clearance originating from the liver. Therefore, ^48^V-activities found in GIT and cumulative fecal excretion during the first 48 hours were excluded from the normalization and the analysis, as it was considered not to be available for translocation through the air-blood-barrier (ABB).

As already published in the Supplementary Information of [[18](#_ENREF_18)] here is a summary of the estimated corrections for ionic ^48^V-release from [^48^V]TiO_2_-NP. For the auxiliary biokinetics study using soluble, ionic ^48^V, the remaining fraction of ionic ^48^V^+^ in the body $a_{\text{ion, body}}^{\text{AUX}}(t)$ at any time $t$ can be written as

|  | $a_{\text{ion,body}}^{\text{AUX}}(t)=1-a_{\text{ion,GIT}}^{\text{AUX}}(t)-a_{\text{ion, feces}}^{\text{AUX}}(t)-a_{\text{ion, urine}}^{\text{AUX}}(t)$, | (26) |
| --- | --- | --- |

where the ^48^V-activity fraction measured in the GIT $a_{\text{ion, GIT}}^{\text{AUX}}(t)$comprises the activity due to ^48^V-ions in the stomach, the small and large intestine, and the oesophagus, while $a_{\text{ion, } \text{feces}}^{\text{AUX}}(t)$and $a_{\text{ion, } \text{urine}}^{\text{AUX}}(t)$ denote the fractions of applied activity that has been accumulated in fecal and urinary excretion, respectively, up to the retention time $t$.

After having determined $a_{\text{ion, body}}^{\text{AUX}}(t)$ and the cumulative urinary excretions in the auxiliary study and the main study, $a_{\text{ion, urine}}^{\text{AUX}}(t)$ and $a_{\text{ion, } \text{urine}}^{\text{MAIN}}(t)$, the fractional ion content in the body of the rats in the main study $a_{\text{ion, body}}^{\text{MAIN}}(t)$ can be estimated from

|  |  | (27) |
| --- | --- | --- |

and for each organ_i_:

|  | . | (28) |
| --- | --- | --- |

under the assumption that the urinary excretion of activity is entirely due to ^48^V-ions. While this calculation is done for each rat of the main study, $\bar{a}_{\text{ion, body}}^{\text{AUX}}(t)$ and $\bar{a}_{\text{ion, urine}}^{\text{AUX}}(t)$ indicate that the mean values are used that are obtained from four rats for a given retention time in the auxiliary study.

In order to determine the particulate [^48^V]TiO_2_NP fraction for each organ in the main study, the ionic ^48^V-fraction in organ “i” $a_{\text{ion, organ, i}}^{\text{MAIN}}(t)$ is subtracted from the total measured ^48^V-activity of that organ $a_{\mathrm{organj}}^{tot, main}\left( t \right)$ at each time point $t$as

|  | $a_{\text{NP,organ, i}}^{\text{MAIN}}(t)=a_{\text{tot, organ, i}}^{\text{MAIN}}(t)-a_{\text{ion,organ, i}}^{\text{MAIN}}(t)$ | (29) |
| --- | --- | --- |

1. **[^48^V]TiO_2_-NP accumulation and retention in secondary organs and tissues: Data evaluation and correction for the release of ionic ^48^V from [^48^V]TiO_2_-NP**

[^48^V]TiO_2_-NP cleared from the thoracic airways *via* mucociliary clearance (MCC) will not be available for the translocation across the ABB. Therefore, for calculations of [^48^V]TiO_2_-NP translocation across the ABB, the fraction cleared by MCC was excluded from the complete balance by subtracting the ^48^V-radioactivity contributions of the head (without brain), trachea, gastro-intestinal tract (GIT), and feces obtained during the first two days after IT-instillation from the overall radioactivity balance of each animal and normalizing the activities determined in all other organs and tissues to the new (reduced) balance. Thus, the amount of IT-instilled material at the time $t=0$ that is available for translocation through the ABB is reduced due to MCC within the first 48h after IT-instillation by

|  | $A_{\text{MCC}}(t)=A_{\text{head}-\text{brain}}(t)+A_{\text{trachea}}(t)+A_{\text{GIT}}(t)+A_{\text{feces}}(t)$ | (30) |
| --- | --- | --- |

analogous to Eqn (10), where $A_{\text{head}-\text{brain}}(t;t\leq24h)$ denotes the ^48^V-activity determined for the head without the brain, $A_{\text{trachea}}(t;t\leq24h)$ the ^48^V-activity in the trachea, $A_{\text{GIT}}(t)$ the one in the gastro-intestinal tract and $A_{\text{feces}}(t;t\leq48h)$ the activity of the feces collected during the first 48h. In order to determine the amount of [^48^V]TiO_2_-NP that is translocated through the ABB as a fraction of [^48^V]TiO_2_-NP that are available for translocation, we have to normalize all activity values measured for all organs and tissues ($A_{\text{organ, i}}(t)$) to a value which is smaller than the value $A(t=0)=A_{0}$ (also defined as initially instilled dose, ID) that has been administered by IT-instillation at time $t=0$. Thus, the normalization is done with this reduced activity, defined as the *initial peripheral lung dose* (IPLD), $A_{\text{IPLD}}$, calculated as

|  | $A_{\text{IPLD}}=A_{0}-A_{\text{MCC}}$ . | (31) |
| --- | --- | --- |

The IPLD is a specific value for each rat used in the two retention time groups. The fraction of material accumulated in organs and tissues after passing the ABB can now be determined for each rat by normalizing the activities $A_{\text{organ,i}}(t)$ according to

|  | $a_{\text{organ,i}}(t)=\frac{A_{\text{organ,i}}(t)}{A_{\text{IPLD}}}$ | (32) |
| --- | --- | --- |

All urinary samples and the slow long-term, macrophage-mediated [^48^V]TiO_2_-NP clearance (LT-MC) fractions from days > 2 during the remaining retention time are considered as a part of the initial peripheral lung dose, IPLD, since all ^48^V-activity in urinary excretion must have passed the ABB and the activity included in the long-term macrophage-mediated clearance (LT-MC) was present for sufficient time in the lungs that it could have contributed to translocation through the ABB.

However, when the fast mucociliary [^48^V]TiO_2_-NP fraction (MCC) cleared from the conducting airways was determined in order to determine the absorbed [^48^V]TiO_2_-NP through the gut wall, MCC data are given as fractions $\bar{a}_{\text{MCC}}(t)$ of the IT-instilled dose ID of ionic ^48^V or [^48^V]TiO_2_-NP-radioactivity in the entire rat ($A(t=0)=A_{0}$)

|  | $\bar{a}_{\text{MCC}}=\frac{A_{\text{MCC}}}{A_{0}}$ | (33) |
| --- | --- | --- |

Thus, in this case, the normalization is done for each rat to $A_{0}$ and not to the IPLD value determined in Eqn. (28). A similar equation holds for the auxiliary study. In order to correct the determined activity data in the main study for contributions of free ^48^V-ions, we make the $\bar{a}_{\mathrm{organ}_{i}}^{\mathrm{main}}(t) = \frac{{A\left( \mathrm{Bq} \right)}_{\mathrm{organ}_{i}}^{\mathrm{main}}(t)}{\sum_{1}^{n} {A(Bq)}_{\mathrm{organ}_{i}}^{\mathrm{main}} (t)}$conservative assumption that all urinary ^48^V-excretion at any time is only of ionic origin and no [^48^V]TiO_2_-NP were excreted in urine.

For the auxiliary biokinetics study using soluble, ionic ^48^V, the remaining fraction of ionic ^48^V^+^ in the body $a_{\text{ion, body}}^{\text{AUX}}(t)$ at any time $t$ can be written as

|  | $a_{\text{ion,body}}^{\text{AUX}}(t)=1-a_{\text{ion,GIT}}^{\text{AUX}}(t)-a_{\text{ion, feces}}^{\text{AUX}}(t)-a_{\text{ion, urine}}^{\text{AUX}}(t)$, | (34) |
| --- | --- | --- |

where the ^48^V-activity fraction measured in the GIT $a_{\text{ion, GIT}}^{\text{AUX}}(t)$comprises the activity due to ^48^V-ions in the stomach, the small and large intestine, and the oesophagus, while $a_{\text{ion, } \text{feces}}^{\text{AUX}}(t)$and $a_{\text{ion, } \text{urine}}^{\text{AUX}}(t)$ denote the fractions of applied activity that has been accumulated in fecal and urinary excretion, respectively, up to the retention time $t$.

After having determined $a_{\text{ion, body}}^{\text{AUX}}(t)$ and the cumulative urinary excretions in the auxiliary and the main study, $a_{\text{ion, urine}}^{\text{AUX}}(t)$ and $a_{\text{ion, } \text{urine}}^{\text{MAIN}}(t)$, the ion content in the body of the rats in the main study $a_{\text{ion, body}}^{\text{MAIN}}(t)$ can be estimated from

|  |  | (35) |
| --- | --- | --- |

under the assumption that the urinary excretion of activity is entirely due to ^48^V-ions. While this calculation is done for each rat of the main study, $\bar{a}_{\text{ion, body}}^{\text{AUX}}(t)$ and $\bar{a}_{\text{ion, urine}}^{\text{AUX}}(t)$ indicate that the mean values are used that are obtained from four rats for a given retention time in the auxiliary study.

In order to be able to estimate $a_{\text{ion, body}}^{\text{MAIN}}(t)$ data throughout the study period of 28 days, the data up to 7 days from the auxiliary study were extrapolated by a least square fit with two exponential terms up to 28 days as shown in Figure S8.

**Figure S8:** (A) Experimentally determined cumulative urinary excretion data up to time $t$ in the auxiliary study ($a_{\text{ion, } \text{urine}}^{\text{AUX}}(t)$) and main study ($a_{\text{ion, } \text{urine}}^{\text{MAIN}}(t)$). (B) ^48^V-ion content $a_{\text{ion, body}}^{\text{AUX}}(t)$ in the body experimentally determined in the auxiliary study and the total ^48^V-activity fraction $a_{\text{tot, body}}^{\text{MAIN}}(t)$ determined in the main study; *i.e.* the sum of ^48^V-ions and [^48^V]TiO_2_-NP activities beyond the lungs. (C) With the data from (A) and (B) the fraction of ions in the body $a_{\text{ion, body}}^{\text{MAIN}}(t)$can be calculated applying Eqn (32); subsequently the $a_{\text{NP, body}}^{\text{MAIN}}(t)$ beyond lungs can be calculated by subtracting the ^48^V-ion contribution from the total activity. The extrapolations of the data from the auxiliary study beyond day 7 were done using least square fits with two exponential terms. For this purpose normalisation was done to the total applied dose ID. Data are mean $\pm$ SEM, n = 4. (2-column fitting image)

The total fraction of ^48^V-activity in the organism at retention time $t$ is determined using cumulative fractions ɑ_feces_(t) and ɑ_urine_(t) (without feces collected during the first 48h) as

|  | $a_{\text{tot, body}}(t)=1-a_{\text{feces}}(t,t>48\text{h})-a_{\text{urine}}(t)$ . | (36) |
| --- | --- | --- |

This equation holds for the main and the auxiliary study. The fraction of [^48^V]TiO_2_-NP in the body beyond the lungs is calculated by the difference between $a_{\text{tot, body}}^{\text{MAIN}}(t)$and $a_{\text{ion, body}}^{\text{MAIN}}(t)$ and the contemporary total lung dose ${}{a_{lungs, fit}^{\mathrm{main}}\left( t \right)}$ at any time $t$according to

|  | $a_{\text{NP, body}}^{\text{MAIN}}(t)=a_{\text{tot, body}}^{\text{MAIN}}(t)-a_{\text{ion,body}}^{\text{MAIN}}(t)-a_{\text{fit, lungs}}^{\text{MAIN}}(t)$ $- {}{a_{lungs, fit}^{\mathrm{main}}\left( t \right)}$ | (37) |
| --- | --- | --- |

An analogous equation holds for the auxiliary study. The contemporary lung dose ${}{a_{lungs, fit}^{NP, main}\left( t \right)}$ is derived from an exponential fit (least square method; Solver, Frontline Systems, Inc., Incline Village, USA) using two term

|  | $a_{\text{fit,lungs}}^{\text{MAIN}}(t)=b_{1}\cdot\exp\left(-\frac{t\cdot\ln2}{t_{1}}\right)+b_{1}\cdot\exp\left(-\frac{t\cdot\ln2}{t_{2}}\right)$ $- {}{a_{lungs, fit}^{\mathrm{main}}\left( t \right)}$ | (38) |
| --- | --- | --- |

as shown in Figure S9.

**Figure S9:** Retention of total lungs (contemporary total lung dose, including BAL) and a mono exponential fit (least square method); fit parameters are given (least square sum is 0.00369). (1-column fitting image)

Having derived an estimate for the fraction of ^48^V-ions in the body we can further estimate the distribution of these ions among the various organs with the help of the data obtained in the auxiliary study, which yields

|  | . | (39) |
| --- | --- | --- |

In order to determine the [^48^V]TiO_2_-NP fraction for each organ in the main study, the ionic ^48^V-fraction in organ “i” $a_{\text{ion, organ, i}}^{\text{MAIN}}(t)$ is subtracted from the total measured ^48^V-activity of that organ $a_{\mathrm{organj}}^{tot, main}\left( t \right)$ at each time point $t$as

|  | $a_{\text{NP,organ, i}}^{\text{MAIN}}(t)=a_{\text{tot, organ, i}}^{\text{MAIN}}(t)-a_{\text{ion,organ, i}}^{\text{MAIN}}(t)$ | (40) |
| --- | --- | --- |

The [^48^V]TiO_2_-NP data at the retention time points 1h, 4h and 24h were corrected with the 24h data of the auxiliary study, and those at 7d and 28d were corrected with the auxiliary data from day 7. For the correction of the ^48^V-ion contribution beyond day 7, the data from the auxiliary study were extrapolated as shown in Figure S5. The effect of the corrections can be assessed from the tabulated data (Table 3, main document).

1. **References**

1. Kreyling WG, Semmler M, Erbe F, Mayer P, Takenaka S, Schulz H, Oberdörster G, Ziesenis A: **Translocation of ultrafine insoluble iridium particles from lung epithelium to extrapulmonary organs is size dependent but very low.** *Journal of Toxicology and Environmental Health-Part A* 2002, **65:**1513-1530.

2. Semmler M, Seitz J, Erbe F, Mayer P, Heyder J, Oberdorster G, Kreyling WG: **Long-term clearance kinetics of inhaled ultrafine insoluble iridium particles from the rat lung, including transient translocation into secondary organs.** *Inhalation Toxicology* 2004, **16:**453-459.

3. Osier M, Oberdorster G: **Intratracheal inhalation vs intratracheal instillation: differences in particle effects.** *Fundamental and Applied Toxicology* 1997, **40:**220-227.

4. Kreyling WG, Möller W, Holzwarth U, Hirn S, Wenk A, Schleh C, Schäffler M, Haberl N, Gibson N, Schittny JC: **Age-dependent deposition patterns and quantitative bio-kinetics of inhaled 20nm gold nanoparticles in rat lungs.** *ACS Nano* submitted**:**(submitted).

5. Kreyling WG, Möller W, Holzwarth U, Hirn S, Wenk A, Schleh C, Schäffler M, Haberl N, Gibson N, Schittny JC: **Age-Dependent Rat Lung Deposition Patterns of Inhaled 20 Nanometer Gold Nanoparticles and their Quantitative Biokinetics in Adult Rats.** *ACS Nano* 2018, **12(8):**7771-7790.

6. (A.R.A.) ARA: **Multiple-path particle dosimetry model (MPPD version 3.0).** 2009.

7. Hirn S, Semmler-Behnke M, Schleh C, Wenk A, Lipka J, Schaffler M, Takenaka S, Moller W, Schmid G, Simon U, Kreyling WG: **Particle size-dependent and surface charge-dependent biodistribution of gold nanoparticles after intravenous administration.** *European Journal of Pharmaceutics and Biopharmaceutics* 2011, **77:**407-416.

8. Schleh C, Semmler-Behnke M, Lipka J, Wenk A, Hirn S, Schaffler M, Schmid G, Simon U, Kreyling WG: **Size and surface charge of gold nanoparticles determine absorption across intestinal barriers and accumulation in secondary target organs after oral administration.** *Nanotoxicology* 2012, **6:**36-46.

9. Kreyling WG, Hirn S, Moller W, Schleh C, Wenk A, Celik G, Lipka J, Schaffler M, Haberl N, Johnston BD, et al: **Air-blood barrier translocation of tracheally instilled gold nanoparticles inversely depends on particle size.** *ACS Nano* 2014, **8:**222-233.

10. Kreyling WG, Hirn S, Möller W, Schleh C, Wenk A, Celik G, Lipka J, Schäffler M, Haberl N, Johnston BD, et al: **Air–Blood Barrier Translocation of Tracheally Instilled Gold Nanoparticles Inversely Depends on Particle Size.** *Acs Nano* 2014, **8:**222 - 223.

11. Kreyling WG, Biswas P, Messing ME, Gibson N, Geiser M, Wenk A, Sahu M, Deppert K, Cydzik I, Wigge C, et al: **Generation and characterization of stable, highly concentrated titanium dioxide nanoparticle aerosols for rodent inhalation studies.** *Journal of Nanoparticle Research* 2011, **13:**511–524.

12. Semmler-Behnke M, Takenaka S, Fertsch S, Wenk A, Seitz J, Mayer P, Oberdorster G, Kreyling WG: **Efficient elimination of inhaled nanoparticles from the alveolar region: evidence for interstitial uptake and subsequent reentrainment onto airways epithelium.** *Environmental Health Perspectives* 2007, **115:**728-733.

13. Bolle I, Eder G, Takenaka S, Ganguly K, Karrasch S, Zeller C, Neuner M, Kreyling WG, Tsuda A, Schulz H: **Postnatal lung function in the developing rat.** *Journal of Applied Physiology* 2008, **104:**1167-1176.

14. Charkes ND, Brookes M, Makler PT, Jr.: **Studies of skeletal tracer kinetics: II. evaluation of a five-compartment model of [18F]fluoride kinetics in rats.** *J Nucl Med* 1979, **20:**1150-1157.

15. Oeff K, Konig A: **[Blood volume of rat organs and residual amount of blood after blood letting or irrigation; determination with radiophosphorus-labeled erythrocytes.].** *Naunyn Schmiedebergs Arch Exp Pathol Pharmakol* 1955, **226:**98-102.

16. Lee HB, Blaufox MD: **Blood volume in the rat.** *J Nucl Med* 1985, **26:**72-76.

17. Kreyling WG, Holzwarth U, Haberl N, Kozempel J, Hirn S, Wenk A, Schleh C, Schäffler M, Lipka J, Semmler-Behnke M, Gibson N: **Quantitative biokinetics of titanium dioxide nanoparticles after intravenous injection in rats: Part 1.** *Nanotoxicology* 2017, **11:**434-442.

18. Kreyling WG, Holzwarth U, Haberl N, Kozempel J, Wenk A, Hirn S, Schleh C, Schäffler M, Lipka J, Semmler-Behnke M, Gibson N: **Quantitative biokinetics of titanium dioxide nanoparticles after intratracheal instillation in rats: Part 3.** *Nanotoxicology* 2017, **11:**454-464.

1. ^1^Note, based on [^198^Au]AuNP per gram of organ or tissue the concentration fractions of soft tissue are certainly much lower as shown in Table 4. [↑](#footnote-ref-1)
